# Factors associated with mental health resilience in the child, adolescent and adult offspring of depressed parents: A systematic literature review

**DOI:** 10.1016/j.jadr.2025.100983

**Published:** 2025-12

**Authors:** Eglė Padaigaitė-Gulbinienė, Jessica Mayumi Maruyama, Gemma Hammerton, Frances Rice, Stephan Collishaw

**Affiliations:** a Wolfson Centre for Young People’s Mental Health, Section of Child and Adolescent Psychiatry, Division of Psychological Medicine and Clinical Neurosciences, Cardiff University, Cardiff, UK; b Centre for Neuropsychiatric Genetics and Genomics, School of Medicine, Cardiff University, Cardiff, UK; c Departamento de Medicina Preventiva, Faculdade de Medicina FMUSP, Universidade de São Paulo, São Paulo, Brasil; d Centre for Academic Mental Health, Population Health Sciences, Bristol Medical School, University of Bristol, Bristol, UK; f Medical Research Council Integrative Epidemiology Unit at the University of Bristol, Population Health Sciences, Bristol Medical School, University of Bristol, Bristol, UK

**Keywords:** Mental health, Resilience, Protective factors, Parental depression

## Abstract

Offspring of depressed parents are at high risk for mental health problems. Nevertheless, some of them do not develop mental health difficulties or do so only temporarily, implying that certain modifiable protective factors could buffer parental-depression-related effects. This systematic review aimed to 1) review the evidence for protective factors enhancing mental health resilience in the offspring of depressed parents; 2) identify outcome-, developmental-stage, and sex-specific protective factors; and 3) summarise conceptual and operational definitions of mental health resilience. We searched PsycINFO, Embase, MEDLINE, Web of Science Core Collection, and Cochrane Library in March 2021. Two reviewers blinded to each other’s decisions independently screened abstracts and full texts against pre-determined eligibility criteria, extracted data, and performed risk of bias assessments. Sixty studies (*N* = 52,966 offspring) examining 70 protective factors were included. Most studies were from high-earning countries (97 %), defined resilience as the absence of psychopathology (90 %), and considered protective factors before young adulthood (97 %) - the peak age for common mental health problems. Most protective factors were examined in only one study (56 %). We observed limited evidence for 10 demographic, parenting, individual, and social protective factors, of which parent-child relationships, co-parent support, and parental positivity were supported across mental health outcomes, and parental positivity was supported across developmental stages. Findings for sex-specific protective factors were inconsistent. Future studies should build further evidence for the protective factors examined and investigate if these associations are causal.

## 1 Introduction

Depression is the leading cause of years lived with disability (James et al., 2018). The peak onset period for mood disorders is in young adulthood (Solmi et al., 2022), which often co-occurs with parenthood. Data from routine treatment records shows that approximately 20 % of children aged 0–16 years in the UK live with a depressed mother (Abel et al., 2019), with this figure rising to over 30 % when adult children are included (age 0–30) (Brophy et al., 2021). Offspring of depressed parents are almost four times more likely to develop depression and other mental health conditions such as anxiety, substance use, conduct disorders, and ADHD (Uher et al., 2023). Both genetic and environmental processes may be involved in the intergenerational transmission of psychopathology, although genetically informed studies suggest that it is predominantly driven by environmental factors (Natsuaki et al., 2014).

Although offspring of depressed parents are at increased risk for psychopathology, a significant minority do not experience mental health difficulties or do so only temporarily (Rutter, 2006; Rutter and Quinton, 1984). The mental health resilience – relative resistance to psychopathology despite risk exposure (Rutter, 2006) – observed in these individuals indicates that certain protective factors may buffer parental depression-related risk effects. Identification of these protective factors and understanding of the processes through which individuals exposed to parental depression overcome experienced adversities would help to identify targets for evidence-based prevention and intervention strategies for those at risk.

Despite resilience being previously identified as a critical pillar of public health and wellbeing by scientific and policy reports (National Research Council, 2009), evidence of which protective factors explain variation in mental health outcomes in the offspring of depressed parents is limited. Two previous narrative reviews summarised a wide range of individual, social, and familial factors that could enhance resilience in offspring of depressed parents (Chen and Kovacs, 2013; Hammen, 2003). Individual-level protective factors that could potentially enhance resilience were intelligence/cognitive skills, an active problem-solving approach (i.e. coping style), ‘high self-understanding’ and positive self-concept, easy-going temperament, and self-efficacy-related factors like secure attachment and feelings of accomplishment (Chen and Kovacs, 2013; Hammen, 2003). Social factors identified as important included positive peer relationships, social competence, social support from extended family members and other non-parental adults, and interactions with prosocial peers. Finally, family factors, such as low current maternal depression, absence of paternal diagnosis, family stress, paternal presence at home, positive perception of mother’s parenting behaviours, maternal social competence, effective communication, consistent parental monitoring and flexibility were also considered as important (Chen and Kovacs, 2013; Hammen, 2003).

However, previous attempts at reviewing the evidence were either non-systematic or did not consider if these factors were sex-, outcome- or developmental-stage specific, were limited to specific offspring ages or focused on interventions only (Beardslee et al., 2011; Chen and Kovacs, 2013; Downey and Coyne, 1990; Hammen, 2003; Loechner et al., 2018a; Reuben and Shaw, 2015; Van Schoors et al., 2023). Therefore, this systematic review aimed to systematically synthesise studies examining protective factors associated with mental health resilience in offspring of depressed parents. Protective factors were defined as those that could reduce or buffer risk associations between parental depression and offspring mental health outcomes (Wright et al., 2013). Additionally, we evaluated the strength of evidence for different outcomes (emotional, behavioural and general psychopathology/mental health resilience), developmental stages (toddlerhood, childhood, adolescence, young adulthood, and adulthood), and sex-specific protective factors, since research suggests that protective factors might differ by mental health outcome, sex, or be developmental stage specific (Collishaw et al., 2016; Kendler and Gardner, 2014; Padaigaitė-Gulbinienė et al., 2024). We also provided an overview of different conceptual and operational definitions of mental health resilience used in previous studies.

## 2 Methods

The protocol for this systematic review (Padaigaitė et al., 2022) was developed using the Preferred Reporting Items for Systematic Review and Meta-Analyses Protocols (PRISMA-P) guidelines (Page et al., 2021). It has been registered in the International Prospective Register of Systematic Reviews (PROSPERO) database (www.crd.york.ac.uk/PROSPERO, CRD42021229955), peer-reviewed, and published (Padaigaitė et al., 2022).

### 2.1 Search strategy and selection criteria

We searched PsycINFO, Embase, MEDLINE, Web of Science Core Collection, and Cochrane Library up to March 15, 2021, with no date restrictions and filters applied. Search terms and index words were grouped into five categories: parents/caregivers, depression, offspring, protective factors/mental health resilience, and exclusion terms. All retrieved records were imported to the EndNote™ library and automatically deduplicated. Titles and abstracts were screened using the systematic literature review software Rayyan (Ouzzani et al., 2016), while full-text screening was performed and documented in pre-specified Excel sheets. Study selection, data extraction, and risk of bias assessments were independently performed by two reviewers (EPG and JMM). Inter-rater agreement at the full-text screening stage was evaluated by calculating Cohen’s Kappa coefficient. Reviewer discrepancies were resolved during consensus meetings with a senior researcher (SC).

All studies were assessed against the following inclusion criteria: 1) written in English and published in a peer-reviewed journal; 2) observational study (prospective and retrospective cohort, case-control or cross-sectional); 3) examined protective factors at any developmental stage (from childbirth to adulthood); both high-risk studies examining the main effects of protective factors and population cohorts examining moderators of the association between parental depression and offspring mental health outcomes were eligible for inclusion; 4) at least one of the child’s parents/caregivers in the study met clinical or research International Classification of Diseases (ICD) or Diagnostic and Statistical Manual of Mental Disorders (DSM) criteria for a depressive disorder for high-risk studies, or reported depressive symptoms for population studies; 5) reported effect sizes of protective factors; 6) reported common mental health problems in offspring (diagnosis or symptoms of an emotional or behavioural disorder or where mental health resilience was operationalised as an outcome using composite measures of mental health). No restrictions were applied for the definition of mental health resilience. We deviated from the published protocol by excluding randomised controlled trials (RCTs) from this systematic review. This was done because a large number of studies were identified of which RCTs were a minority, and a systematic review and meta-analysis of RCTs in offspring of depressed parents was published previously (Loechner et al., 2018).

### 2.2 Data extraction and analysis

For each retained study, two reviewers extracted key study characteristics described elsewhere (Padaigaitė et al., 2022). If multiple models and effect sizes were presented in the study, the univariable model adjusted for confounders with standardised effect sizes (e.g. β) was prioritised. The risk of bias was assessed using Joanna Briggs Institute (JBI) critical appraisal checklists for cohort and cross-sectional studies (Moola et al., 2020). Every study was evaluated against every item and scored as yes (1), no (0), unclear (0), or not applicable (N/A). Consistent with prior research, studies with < 50 % of ‘yes’ answers were considered at a high risk of bias, 50 – 69 % at a moderate risk, while studies scoring 70 % or more were considered at a low risk of bias (Franco et al., 2020).

Due to high heterogeneity across and within studies, meta-analysis was not attempted. Instead, this systematic review used a vote-counting approach (De Brier et al., 2020). In this systematic review, the strength of evidence for each protective factor was evaluated by 1) considering the number of studies that examined a specific factor and 2) the percentage of models within these studies that found evidence for association (Cortese et al., 2023). This approach was taken as studies usually examined the same protective factor in multiple models (e.g. in relation to multiple outcomes or used multiple informants). Considering the large number of protective factors identified and limited evidence for most of them, this systematic review primarily focused on protective factors that were successfully replicated at least once (i.e. examined in at least two independent studies and replicated in >50 % of the models (based on the direction of the association and p-value)).

First, we described main study characteristics, conceptual and operational definitions of mental health resilience used, and the most and least examined protective factor domains (demographic, family, parenting, childcare, individual, social, lifestyle, and school) in relation to different mental health outcome categories (emotional, behavioural, and composite measures of mental health/resilience). We decided to use the original names of protective factors used by authors. However, there is some uncertainty regarding how domains are labelled since authors often used different names for similar constructs (e.g. parental warmth or positivity in the parenting domain). Then, using the criteria above, we summarised the evidence for each protective factor by identifying the most and least supported protective factors across all studies included in the systematic review (irrespective of mental health outcomes or developmental stage) and by identifying outcome-, developmental stage-, and sex-specific protective factors. For developmental-stage specific protective factors, studies were categorised into toddlerhood (ages 1 to 3), childhood (ages 4 to 10), adolescence (ages 11 to 17), young adulthood (ages 18 to 25), and adulthood (older than 25 years old) based on the offspring age at the time of mental health outcome assessment. Studies assessing mental health outcome trajectories were assigned to the developmental stage based on the latest/oldest age at assessment, while studies examining outcomes at more than one developmental stage were summarised twice.

## 3 Results

**Fig. 1:**
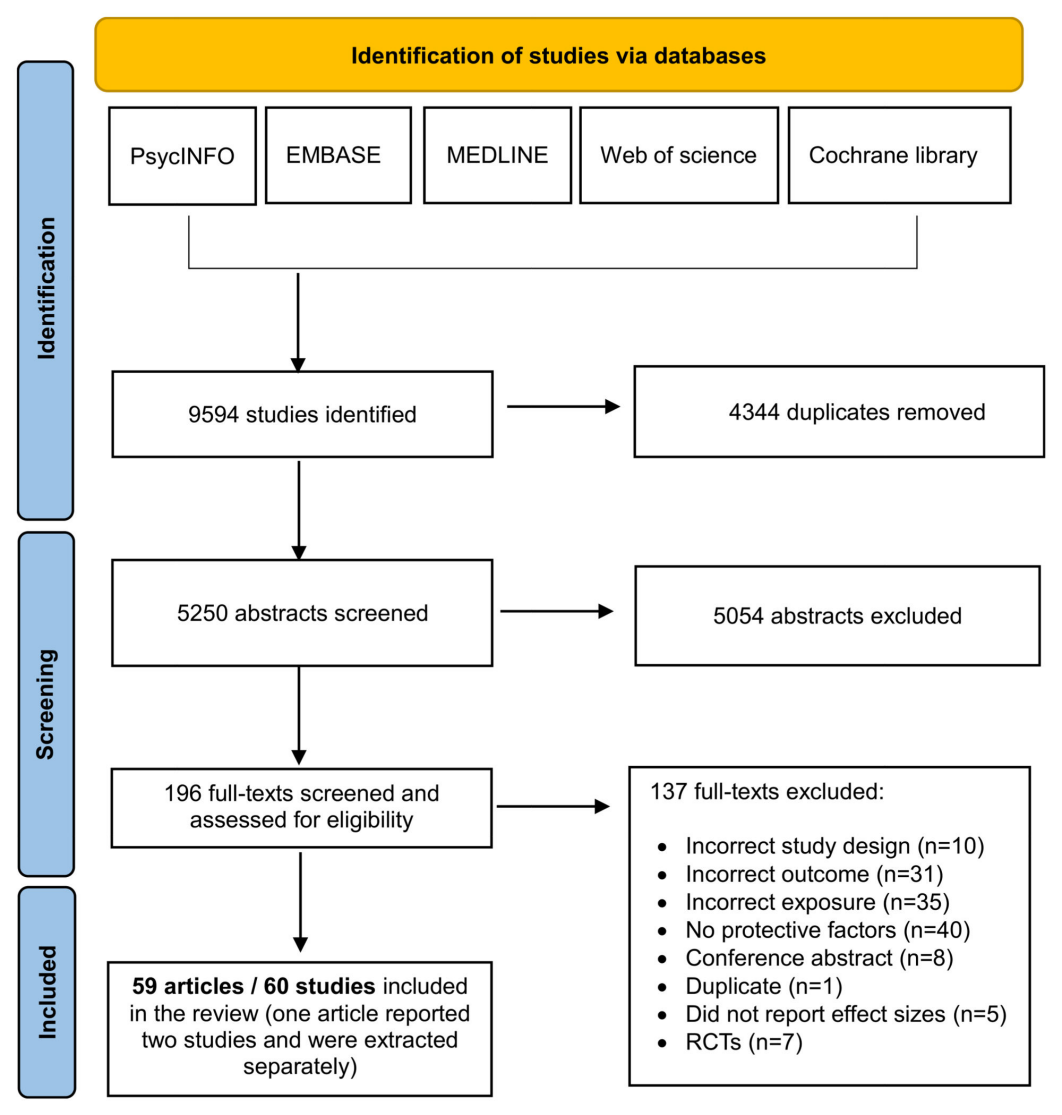
Selection of studies eligible for inclusion.

The database search identified 9594 studies, of which 4344 were duplicates, leaving 5250 studies. We screened 5250 abstracts and 196 full texts and identified 59 articles describing 60 studies (*N* = 52,966 offspring of depressed parents) eligible for inclusion (see Fig. 1). At the full-text screening stage, the raters had a substantial agreement (84.2 %; Cohen’s *k* = 0.67). All initial disagreements were resolved during discussions or by consulting a senior researcher. Studies that did not meet inclusion criteria at the full-text screening stage and reasons for exclusion are described in Table S1.

### 3.1 Characteristics of included studies

All included studies are described in Table 1, while study characteristics are summarised in Table S2. Most studies (97 %) were under-taken in high-earning countries: USA (*N* = 40; 66.7 %), UK (*N* = 6; 10.0 %), Canada (*N* = 4; 6.7 %), Australia (*N* = 3; 5 %), Israel (*N* = 2; 3.3 %), China (*N* = 1; 1.7 %), Germany (*N* = 1; 1.7 %), Netherlands (*N* = 1; 1.7 %), Norway (*N* = 1; 1.7 %), Taiwan (*N* = 1; 1.7 %). Sample sizes ranged from 51 to 11,286, with a median of 187. Eight (13.3 %) studies did not report offspring sex, but of those that did, the median percentage of females was 51 %. Of those included, 17 (28.3 %) studies examined the main effects of protective factors on offspring mental health outcomes in high-risk cohorts, and 43 (71.7 %) examined the moderating role of protective factors on the relationship between parental depressive symptoms and offspring outcomes in population cohorts. More studies were prospective cohorts (*N* = 35; 58.3 %) than cross-sectional studies (*N* = 25; 41.7 %). The time lag between the protective factor and outcome assessment in longitudinal studies ranged between 1 and 20 years, with a median time lag of 3 years. Most studies examined maternal depression only (*N* = 37; 61.7 %), 1 study (1.7 %) examined paternal depression only, 6 studies (10.0 %) examined maternal and paternal depression separately, while the remaining studies examined the role of parental depression (*N* = 16; 26.7 %). Studies assessed parental depression using self-reported depressive symptoms questionnaires (*N* = 31; 50.8 %), clinical diagnoses (*N* = 28; 45.9 %) or both (*N* = 2; 3.3 %). The majority of studies examined mental health outcomes before young adulthood (88 %): infancy (up to age 1; *N* = 0; 0 %), toddlerhood (*N* = 5; 8.2 %), childhood (*N* = 26; 42.6 %), adolescence (*N* = 23; 37.7 %), young adulthood (*N* = 2; 3.3 %), and adulthood (*N* = 5; 8.2 %). As summarised in Tables S3 and **S4**, most studies (88 %) were at low risk of bias.

### 3.2 Mental health outcomes and definitions of mental health resilience

Included studies examined a range of mental health outcomes that were categorised into 3 broad categories: emotional (depression, anxiety, internalising problems), behavioural (conduct disorder (CD), oppositional defiant disorder (ODD), externalising problems), and general mental health outcomes (composite of different disorders/symptoms). Considering the conceptual and operational definitions of mental health resilience, most studies (90 %) defined it as the absence of psychopathology and examined factors that reduce the likelihood of one or several specific mental health outcomes. Three studies (Collishaw et al., 2016; Lewandowski et al., 2014; Mahedy et al., 2018) used multiple definitions of mental health resilience. In addition to the absence of psychopathology/sustained good mental health across development, they also examined resilience as better-than-expected mental health outcomes (Collishaw et al., 2016; Mahedy et al., 2018) or high functioning (Lewandowski et al., 2014). Two studies (Brennan et al., 2003; Pargas et al., 2010) derived composite scores for resilience capturing the absence of psychopathology and good social or academic functioning, while one study (Giallo et al., 2018) identified resilient individuals as those that scored within the normal range for emotional and behavioural problems despite being exposed to high or moderate maternal depression. One study (Sun et al., 2015) defined resilience as a trait and examined its buffering role on internalising and externalising symptoms.

### 3.3 Most and least studied protective factor domains

**Fig. 2:**
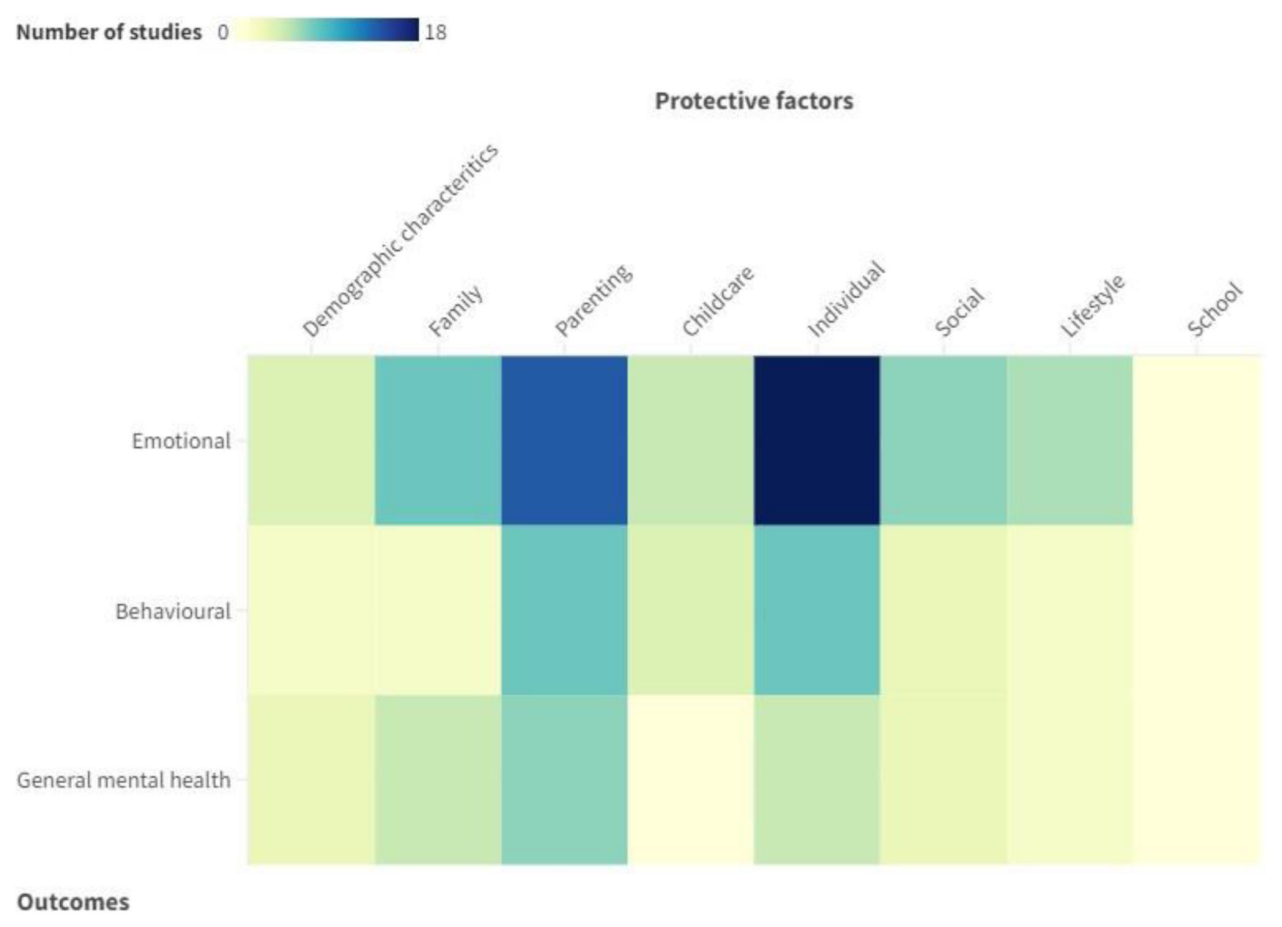
Number of studies presented by protective factor category and mental health outcome. A darker colour denotes a larger number of studies examining a particular protective factor in relation to the outcome.

Given the breadth of predictors examined and the differing labels used by authors, protective factors were categorised into demographic (e.g. income, maternal age), family (e.g. grandmother living in the household, family functioning), parenting (e.g. parenting skills, attachment quality), childcare (e.g. quality, childcare by partner or relative), individual (e.g. self-esteem, physiological reactivity), social (e.g. social skills, prosocial friends), lifestyle and beliefs (e.g. religiosity, exercise), and school (e.g. teacher support, academic performance) factors. As summarised in Fig. 2, most studies examined the protective role of individual, parenting, family, and social factors in relation to emotional problems, while school, childcare, demographic, and lifestyle factors were examined the least across mental health outcomes.

### 3.4 Most and least supported protective factors across all studies

**Fig. 3:**
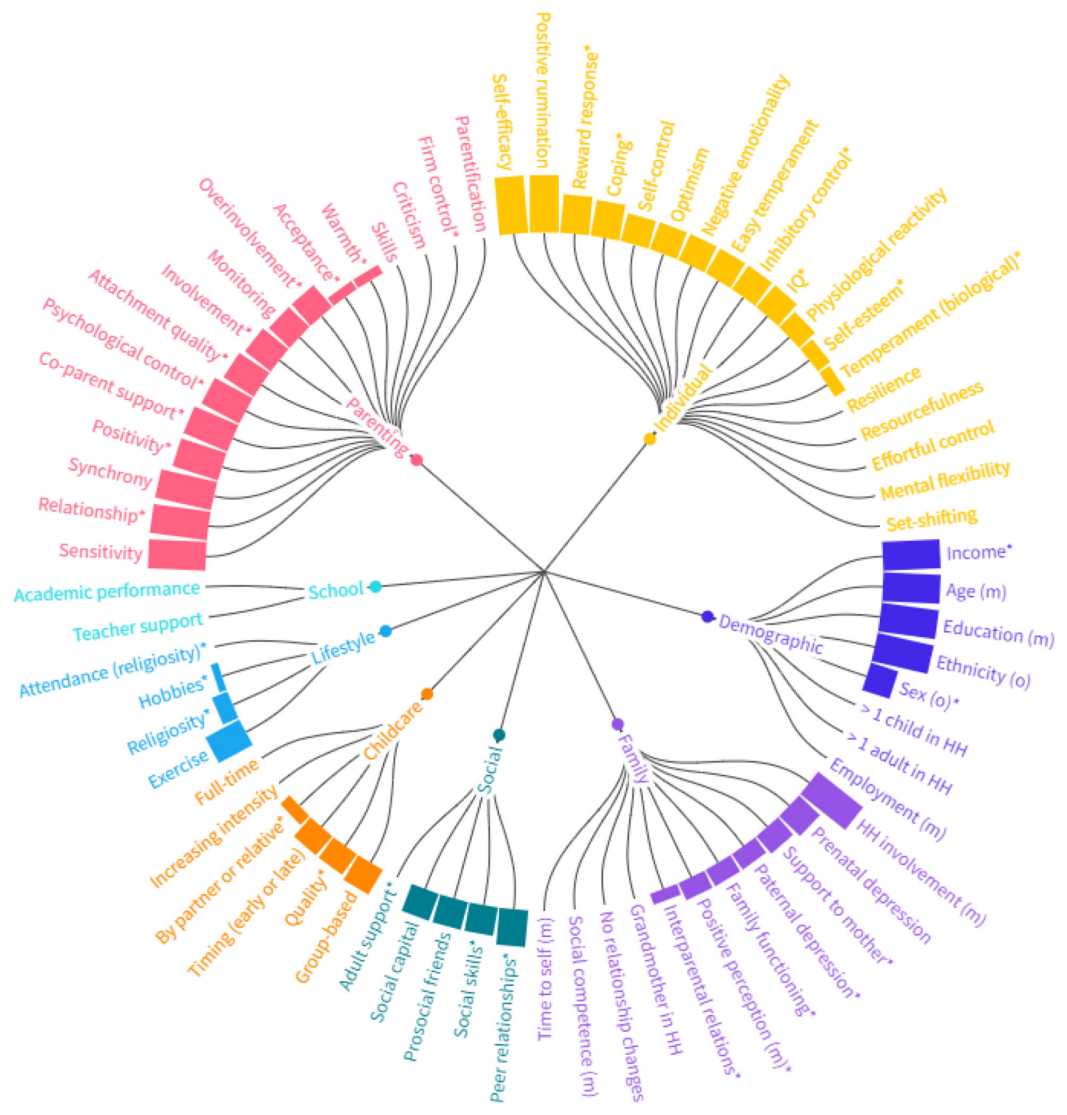
The overall strength of evidence for each protective factor was summarised as a percentage of models that found an association with common mental health outcomes in the offspring of depressed parents. An asterisk marks protective factors examined by at least 2 studies. M – mother/maternal; O – offspring; HH – household; IQ – intelligence quotient.

Table 2 summarises the evidence for all 70 protective factors examined in the included studies, including the number of studies that examined each protective factor (in relation to the developmental stage, outcome and study design) and the number and percentage of the statistical models where evidence for association was observed (using *p* < 0.05 threshold). These findings are further summarised visually in Fig. 3. Despite the relatively large number of included studies, the majority (56 %) of protective factors were examined only by one study. Only 10 demographic, parenting, childcare, individual, and social factors were examined in >2 studies, and the protective role of the factor was observed in >50 % of the models. Higher household income (percentage of models that found evidence for association across all studies: 100 %) (Giallo et al., 2018; Graham and Easterbrooks, 2000) was associated with emotional-behavioural functioning and depressive symptoms in childhood. Most strongly supported parenting factors were high-quality parent-child relationships (100 %) (Malmberg and Flouri, 2011; Manczak et al., 2018), expressed positive emotions from parents (83 %) (Collishaw et al., 2016; Goodlett et al., 2017; West et al., 2020), co-parent support (83 %) (Collishaw et al., 2016; Mahedy et al., 2018), low parental psychological control (75 %) (Brennan et al., 2003; Pargas et al., 2010), attachment quality (63 %) (Carlone and Milan, 2021; Fox and Borelli, 2015; Graham and Easterbrooks, 2000; Milan et al., 2009; Woodhouse et al., 2010), and parental involvement (57 %) (Chang et al., 2007; Harold et al., 2014; West et al., 2020). Most strongly supported individual factors were reward response (67 %) (Kujawa et al., 2019; Silk et al., 2006) and stress-coping skills (63 %) (Monti and Rudolph, 2017; Vreeland et al., 2019), while three studies found evidence for the protective role of high-quality peer relationships (64 %) (Collishaw et al., 2016; Conrad and Hammen, 1993; Pargas et al., 2010).

Based on the same criteria, least supported family and parenting factors were partner or family support to mother (40 %) (Boyd and Waanders, 2013; Giallo et al., 2018; Lee et al., 2006), family functioning (33 %) (Havinga et al., 2017; Lewandowski et al., 2014), child’s positive perception of a mother (33 %) (Andreas et al., 2017; Conrad and Hammen, 1993), paternal depression status (33 %) (Brennan et al., 2003; Conrad and Hammen, 1993; Gere et al., 2013; Malmberg and Flouri, 2011), interparental relationship quality (20 %) (Giallo et al., 2018; Lewandowski et al., 2014; Taraban et al., 2020), parental acceptance (17 %) (Brennan et al., 2003; Owens and Shaw, 2003; Pargas et al., 2010), parental or sibling warmth (17 %) (Brennan et al., 2003; Chen, 2013; Collishaw et al., 2016; Lewandowski et al., 2014; Pargas et al., 2010), and parental firm control (0 %) (Brennan et al., 2003; Pargas et al., 2010). Least supported childcare factors were childcare quality (44 %) (Charrois et al., 2017; Goelman et al., 2014) and childcare by partner or relative (22 %) (Smith et al., 2013; Giallo et al., 2018; Herba et al., 2013; Lee et al., 2006). Least supported individual and lifestyle factors were self-esteem (27 %) (Abela et al., 2012; Abela and Skitch, 2007; Chang and Fu, 2020; Conrad and Hammen, 1993; Lewandowski et al., 2014; Pargas et al., 2010), biological markers of temperament (22 %) (Davis et al., 2016; Shannon et al., 2007), out-of-school activities (13 %) (Bohnert and Garber, 2007; Collishaw et al., 2016), religiosity (29 %) (Jacobs et al., 2012; Kasen et al., 2012; Miller et al., 2012), and attendance at religious services (0 %) (Barton et al., 2013; Jacobs et al., 2012; Kasen et al., 2012; Miller et al., 2012).

### 3.5 Most and least supported protective factors across mental health outcomes

Protective factors by mental health outcomes are summarised in Table S5. Only 3 parenting factors were protective across at least 2 mental health outcomes. Parent-expressed positive emotion was associated with general mental health/resilience (100 %) (Collishaw et al., 2016; West et al., 2020), as well as emotional (67 %) (Collishaw et al., 2016; Goodlett et al., 2017) and behavioural (100 %) (Collishaw et al., 2016) outcomes. Co-parent support was associated with emotional (100 %) (Collishaw et al., 2016; Mahedy et al., 2018) and general mental health/resilience (100 %) (Collishaw et al., 2016; Mahedy et al., 2018), but its association with behavioural outcomes was inconsistent (50 %) (Collishaw et al., 2016; Mahedy et al., 2018). Parent-child relationship quality was protective for both emotional (100 %) (Malmberg and Flouri, 2011; Manczak et al., 2018) and behavioural (100 %) (Manczak et al., 2018) outcomes, but no studies examined its association with general mental health/resilience. Inconsistent findings were observed for offspring sex, parental involvement, childcare quality, and self-esteem. Parental or sibling warmth, childcare by partner or relative, and out-of-school activities were least supported across all mental health outcomes.

### 3.6 Most and least supported protective factors across developmental stages

Developmental stage-specific protective factors are summarised in Table S6. As might be expected, different protective factors were examined at each developmental stage. None of the protective factors were examined across all developmental stages. Seven demographic, family, parenting, and individual protective factors were examined across 3 or more developmental stages, of which the protective role of parent-expressed positive emotion was most strongly supported: associations with mental health outcomes were observed across toddlerhood (100 %) (Goodlett et al., 2017), childhood (100 %) (Goodlett et al., 2017; West et al., 2020), and adolescence (67 %) (Collishaw et al., 2016). Offspring sex was not associated with mental health outcomes in toddlerhood (0 %) (Lee et al., 2006), but associations emerged in childhood (67 %) (Giallo et al., 2018; Turney, 2011) and adulthood (100 %) (Havinga et al., 2017). Associations between mental health outcomes and two family factors: paternal depression status and partner or family support to mother, also varied by developmental stage. Paternal depression status was associated with mental health outcomes in childhood (67 %) (Gere et al., 2013) but not toddlerhood (0 %) (Malmberg and Flouri, 2011) or adolescence (0 %) (Brennan et al., 2003; Conrad and Hammen, 1993), while partner or family support to mother was associated with mental health outcomes at earlier developmental stages: in toddlerhood (50 %) (Lee et al., 2006) and childhood (100 %) (Giallo et al., 2018), but not adolescence (0 %) (Boyd and Waanders, 2013). Considering individual and parenting factors, self-esteem was not associated with mental health outcomes in childhood (0 %) (Abela et al., 2012; Abela and Skitch, 2007), adolescence (0 %) (Conrad and Hammen, 1993) or young adulthood (20 %) (Chang and Fu, 2020; Pargas et al., 2010), but association emerged in adulthood (100 %) (Lewandowski et al., 2014). Limited evidence was observed for parental acceptance association with mental health outcomes in childhood (50 %)(Owens and Shaw, 2003) but not adolescence (0 %) (Brennan et al., 2003) or young adulthood (0 %) (Pargas et al., 2010), while parental or sibling warmth was not associated with mental health outcomes across adolescence (22 %) (Brennan et al., 2003; Chen, 2013; Collishaw et al., 2016), young adulthood (0 %) (Pargas et al., 2010), and adulthood (0 %) (Lewandowski et al., 2014).

### 3.7 Variation in findings by offspring sex

Only six studies (Andreas et al., 2017; Braithwaite et al., 2020; Casey-Cannon et al., 2006; Harold et al., 2014; Monti and Rudolph, 2017; Owens and Shaw, 2003) examined sex-specific effects of protective factors: three examined interactions between parental depression, protective effects, and sex/gender (Andreas et al., 2017; Harold et al., 2014; Owens and Shaw, 2003), while the other three performed stratified analyses (Andreas et al., 2017) or studied protective effects in females (Harold et al., 2014) or males (Owens and Shaw, 2003) only. In one study (Casey-Cannon et al., 2006), sex did not moderate the association between maternal or paternal depression, non-parent adult support, and depressive symptoms in offspring. The buffering role of lower prenatal depressive symptoms on emotional problems was more beneficial for male offspring of depressed parents (Braithwaite et al., 2020). The buffering role of adaptive responses to stress (high effortful engagement and low involuntary disengagement) on initial levels and trajectories of youth depression varied by sex (Monti and Rudolph, 2017). For females, these coping strategies mitigated the maternal depression associations with initial levels of depression, while for males, it mitigated the associations on the depression trajectories (Monti and Rudolph, 2017). In sex-stratified analyses (Andreas et al., 2017), the buffering role of the positive representation of a mother was more supported in females than males. Maternal caregiving involvement was associated with reduced antisocial, but not depressive behaviour in a female-only cohort (Harold et al., 2014), while maternal acceptance was associated with lower externalising symptoms at age 6, and negative emotionality was associated with the lower rate of change in externalising symptoms between ages 2 and 6 in a male-only cohort (Owens and Shaw, 2003). However, results from the studies examining sub-group effects should be interpreted with caution.

## 4 Discussion

This systematic review observed limited evidence (that comes from 2–5 studies) for a protective role of 10 parenting, individual, social, and demographic factors. The most strongly supported parenting factors were the quality of parent-child relationships, parental positivity towards offspring, co-parent support, low psychological control, parental involvement, and attachment quality. Parent-child relationships, parental positivity, and co-parent support were supported across mental health outcomes, while parental positivity was also supported across developmental stages. Theories of the intergenerational transmission of depression highlight aberrant relationships with the primary caregiver as having a central role in the development of psychopathology due to the association of parental depression with more hostile, antagonistic, and disengaged parenting (Lovejoy et al., 2000). However, this systematic review highlighted that families with a depressed parent can utilise effective parenting strategies and provide vital emotional support to their offspring. Identified parenting behaviours could be a potential avenue for interventions for parents with mild or remitted depression (Lannes et al., 2021), but for more severe cases, targeting parental depression itself might be a more efficient strategy (Cuijpers et al., 2015). Our results also highlight the role of fathers in the maternal depression context. Supportive fathers/co-parents could provide instrumental and emotional support, buffer maternal depression-related effects, and contribute to better family functioning and cohesion (Fisher and Glangeaud-Freudenthal, 2023; Vakrat et al., 2018). Therefore, encouraging fathers to participate in interventions to bolster the support they can provide to their families and look after their own mental health may be beneficial in promoting paternal support.

Two individual-level factors identified as protective were enhanced reward response and stress-coping skills. However, enhanced reward response was associated only with emotional outcomes in childhood. Emerging evidence suggests that activation of the reward system can reduce physiological stress reactivity (Dutcher, 2023), potentially leading to better mental health outcomes. Although it has been shown to predict depression, it could also be a consequence of depression (Potsch and Rief, 2023; Rawal et al., 2013). Further research is needed to clarify the direction and causality of these associations and if the offspring of depressed parents would benefit from interventions aimed at increasing reward sensitivity, such as behavioural activation (Rice et al., 2015). The protective role of stress coping skills was also limited to emotional outcomes in adolescence. Although problem-focused coping strategies are usually considered more beneficial than emotion-focused ones (Michelson et al., 2022), in this systematic review, both strategies were beneficial for emotional outcomes, while emotion-focused strategies were beneficial for behavioural outcomes. Furthermore, stress coping strategies were protective for trajectories of offspring depression in a sex-specific manner: in females, these coping strategies mitigated the maternal depression associations with initial levels of depression, while in males, it mitigated the associations with the depression trajectories (Monti and Rudolph, 2017). However, sex-specific associations of other protective factors were rarely examined, potentially leading to inconsistent findings, warranting further study.

Peer relationship quality and household income were also protective. In adolescence, peer relationship quality was associated with emotional and behavioural outcomes but not general psychopathology/resilience, while associations in young adulthood were not supported. Interpersonal relationships likely reflect both individual skills and beneficial effects: individuals who can form, sustain, and benefit from positive relationships with others are more likely to exhibit resilient outcomes (Collishaw et al., 2007). Moreover, positive encounters with peers could buffer negative interactions experienced at home, teach the child more prosocial ways of interacting with others, and help successfully face stressful transitions (Ng-Knight et al., 2019). Despite an increasing body of evidence suggesting that family income is strongly associated with beneficial outcomes and these effects are likely causal (Ridley et al., 2020), studies often included income as a potential confounder rather than a protective factor. Higher household income was protective for emotional and general psychopathology/resilience, and surprisingly, was examined only in childhood. Further studies are needed to examine the causal role of household income in mental health resilience across development and to understand the mechanisms underlying these potentially causal effects.

As expected, studies greatly varied in definitions of resilience, although most examined factors associated with the absence of psychopathology. Alternative definitions included adaptive functioning in addition to absence of psychopathology or better-than-expected mental health outcomes, considering differing levels of parental depression severity. However, all these definitions have limitations. Resilience defined as the lifetime absence of psychopathology, does not consider varying levels of risk exposures nor an individual’s functioning in other life domains and could inadvertently classify individuals with sub-threshold symptoms whose functioning may be impaired as resilient. Definitions considering good social and academic functioning in addition to absence of psychopathology could be considered too rigid, putting unrealistic expectations on resilient individuals to perform successfully across multiple life domains (Luthar et al., 2000). Better-than-expected outcome (e.g. a residual score approach) usually lacks a holistic framework since it considers only one or several specific domains: resilient individuals can be classified as resilient to one condition (e.g. depression) but face challenges in other outcome domains (e. g. behavioural). Considering the low rates of resilience reported among offspring of depressed parents (Collishaw et al., 2016; Maruyama et al., 2023; Padaigaitė-Gulbinienė et al., 2024), future studies could consider using more inclusive/complementary definitions of resilience and examine protective factors that are associated with recovery or a delay in the onset of mental health problems.

### 4.1 Strengths and limitations

This systematic review fills a gap in the literature by providing a comprehensive systematic overview of the most studied and strongly supported protective factors enhancing mental health resilience in offspring of depressed parents, identifying limitations of the current studies, suggesting future research directions, and providing several important implications for clinicians, such as the importance of supportive parenting practices and the role of co-parents. We also took a culturally sensitive approach and considered how unpublished results and methodological differences may have influenced the strength of associations found. Other strengths of this systematic review include the development and publication of a systematic review protocol according to the PRISMA guidelines, electronic searches in 5 databases, independent and blinded data screening, extraction, and risk of bias assessment performed by two reviewers, and using a culturally sensitive approach to describe study findings. Nevertheless, it also has several limitations. First, most protective factors included in this systematic review were examined only once, hindering the ability to draw firm conclusions. Future studies should continue to build evidence for the role of protective factors by examining the least studied factors (demographic, life-style, school, and biological factors) and those identified as protective in only one developmental stage. Second, in line with the scope of the review, only studies that were hypothesised as protective by study authors or, if authors did not specify the expected direction of association, demonstrated to be protective were included in the data synthesis. Therefore, this review could be prone to selective reporting bias. Third, due to limited resources, this systematic review does not consider un-published studies or studies published in other languages. Fourth, this systematic review might have suffered from the ‘jingle-jangle fallacy’ (van Zyl et al., 2024). Multiple similar constructs (e.g. warmth, positivity, affection) were assessed in the studies without clearly defining them or clarifying their differences, making it hard to merge them into categories. Future studies would benefit from using standardised measures and examining the best way to merge similar constructs using statistical approaches such as factor analysis. Furthermore, although the risk of bias tools developed for observational studies were used, some items were not optimal/relevant for high-risk cohorts and had to be tailored. Lastly, the strength of evidence of this systematic review was evaluated based on the number of studies and the percentage of models that found evidence for association (i.e. ‘statistically significant’ results). This might be problematic because sample sizes will determine study power to detect ‚significant’ effects, and by using this approach, we were not able to take this into account. However, comparing effect sizes between protective factors was impossible since studies varied in study designs and measurements, the types of effect sizes, and choice of statistical estimates (e.g. OR, HR, B, β, r^2^, T ratio, F) reported.

Considering the common limitations of included studies, most studies examined protective effects in toddlerhood, childhood, and adolescence before the peak age of the emergence of common mental health problems (Solmi et al., 2022), potentially misclassifying individuals as resilient, which could result in misleading results. More-over, nearly half of the studies were cross-sectional, which preclude causal conclusions. Future prospective longitudinal studies spanning into adulthood are crucial for understanding temporal relationships and the direction of effects of mechanisms underlying long-term resilience (Narita et al., 2025). Likewise, included studies employed different approaches to assess parental depression (i.e. self-reported depressive symptoms using questionnaires or clinical diagnoses using diagnostic interviews). Self-reported depressive symptom questionnaires do not consider distress, impairment, or duration of the symptoms, and do not require symptoms to be present during the same reporting period. Therefore, it might not capture the same level of severity as clinical diagnostic interviews. Furthermore, most studies examining factors promoting resilience in offspring of depressed parents focused on mothers, with only a small proportion (12 %) explicitly examining protective factors buffering paternal depression effects. Given differences in symptom display, help-seeking behaviours, and other aspects of family dynamics, future studies should explore the role of paternal depression on offspring mental health outcomes and examine factors promoting resilience in the paternal depression context (Piccinelli and Wilkinson, 2000).

This systematic review also demonstrated that most evidence comes from high-income Western countries. Therefore, the results of this systematic review cannot be translated to low- and middle-income countries. Different cultures vary in what is considered normative, especially in culturally sensitive factors such as parenting (Bornstein, 2013). Therefore, the role of certain protective factors might not be universal and vary depending on the cultural context. Given that depression is most widespread among mothers in low- and middle-income countries (Wang et al., 2021), it is crucial to include multinational cohorts from these regions to ensure that research findings apply to most of the population and to inform more effective national and global strategies for enhancing mental health outcomes (Wellcome Trust, 2023). Moreover, inconsistent reporting or not reporting results in sufficient detail (i. e. reported model fit estimates instead of effect sizes or not reporting confidence intervals or exact p values) made data synthesis challenging and unsuitable for meta-analysis. Lastly, included studies are also likely susceptible to publication and selective reporting bias since protocol development and study preregistration are not mandatory for observational studies, and unpublished or partly published results cannot be easily identified.

Future studies could test how and why protective factors are associated with resilience. Studies could consider the cumulative effects of protective factors or examine their interrelatedness and identify the mechanisms underlying these associations. Additionally, given that many associations might be bidirectional and reverse causation in observational studies cannot be ruled out, the direction and causality of the protective factors identified should be tested. For instance, in addition to examining protective associations, Mahedy and colleagues (2018) demonstrated that paternal emotional support is associated with offspring depressive symptoms but not vice versa, while Kendler and colleagues (2020) showed that positive rearing environment in adoptive families is a causal protective factor for depression, if a family does not face adversities such as adoptive parent depression, parental divorce or death. Lastly, given the exponential growth of the scientific literature in the biomedical field (Landhuis, 2016), the importance of systematic evidence synthesis will only increase over the years. To enhance the efficiency of this process, researchers could incorporate a supplementary table in their manuscripts containing essential study characteristics, reducing the time needed for data extraction and striving for consistent reporting practices by following established guidelines like Strengthening the Reporting of Observational Studies in Epidemiology (STROBE) (von Elm et al., 2008) or similar frameworks.

## 5 Conclusion

This systematic review evaluated the evidence on protective factors that enhance mental health resilience in offspring of depressed parents. The review identified household income, positive parent-child relationships, parental positivity towards offspring, co-parent support, low parental psychological control, parental involvement, attachment quality, enhanced reward response, adaptive stress-coping skills, and high-quality peer relationships as key protective factors. Parent-child relationships, co-parent support, and parental positivity towards offspring were supported across mental health outcomes, while the latter was also supported across developmental stages. However, findings for sex-specific protective factors were inconsistent. Most of the protective factors were only studied once, leading to insufficient evidence for definitive conclusions. Therefore, further high-quality studies are necessary to establish and replicate the findings for the protective factors that have been less explored, including demographic, lifestyle, school, and biological factors, especially in young adulthood.

## Supplementary Material

Supplementary material associated with this article can be found, in the online version, at 10.1016/j.jadr.2025.100983.

Supplementary Material

## Figures and Tables

**Table 1:** Summary of studies included in the systematic review.

Study	Country	Type of study	Design	Sample size (N)	% of females	Offspring age (M (SD/CI)) at outcome	FU (years)	Risk exposure (assessment used)	Test of protective factor	Protective factor	Effect size (CI / SE) and p-value (adjusted)	Outcome (assessment used)	Key findings	Risk of bias level
**Toddlerhood (1 to 3 years old at outcome)**														
Lee et al. (2006) ⋄	USA	Population	PC	1216	49 %	36 months old	0–3	Maternal depressive symptoms (CES-D)	Moderator	Sex (male)	β = 0.04 (–0.03, 0.10), *p* = 0.30	Externalising symptoms (CBCL)	Social support buffered maternal depression association with a child’s externalising problems but only at low levels of maternal depression. Maternal depressive symptoms association with offspring internalising symptoms was lower in offspring who received more hours of childcare provided by others.	Low
										Childcare by another caregiver (not the mother)	β = 0.00 (–0.03, 0.02), *p* = 0.71			
										Social support from spouse/partner	β = 0.04 (0.00, 0.08), *p* = 0.04			
										Sex (male)	β = 0.05 (–0.00, 0.10), *p* = 0.07	Internalising symptoms (CBCL)
										Childcare by other caregivers (not mother)	β = –0.02 (–0.04, 0.00), *p* = 0.02	
										Social support from spouse/partner	β = 0.03 (0.00, 0.06), *p* = 0.06	
Feldman & Masalha (2007) ●	Israel	Population	PC	162	48 %	34.0 (4.3) months old	2.5	Maternal depressive symptoms (BDI)	Moderator	Ethnicity	β = –0.59, *p* < 0.05	Internalising and externalising symptoms (CBCL)	Ethnicity moderated maternal depressive symptoms association with a child’s internalising and externalising symptoms. Maternal depressive symptoms had a more negative effect in the Israeli group.	Low
Malmberg & Flouri (2011) ◊	UK	Population	PC	11,286	N/R	36 months old	0–2.25	Maternal depressive symptoms (MI)	Moderator	Father’s depressive symptoms	*B* = –0.02 (0.10), *p* > 0.05	Internalising and externalising symptoms (CBCL)	Both mother-child and father-child relationships buffered the association between the other parent’s depressive symptoms and the child’s emotional problems.	Low
										Father-child relationship	*B* = –0.31 (0.08), *p* < 0.05	Emotional problems (SDQ)		
								Paternal depressive symptoms (MI)		Mother-child relationship	*B* = –0.38 (0.11), *p* < 0.05			
Smith et al. (2013) ◊	USA	Population	CS	125	N/R	18.2 (1.0) months old	N/A	Maternal depressive symptoms (SCL-90-R)	Moderator	Perceived partner childcare	β = –0.21, *p* < 0.05	Internalising symptoms (CBCL)	Perceived partner childcare moderated maternal depression association with internalising but not externalising problems.	Low
											β = –0.01, *p* > 0.05	Externalising symptoms (CBCL)		
Goodlett et al. (2017) ◊	USA	Population	PC	102	N/R	37.2 (33.5) months old	1	Maternal depressive symptoms (BDI)	Moderator	Positive parenting	*B* = –0.03, *p* < 0.05	Internalising symptoms (CBCL)	The risk of child internalising problems in the presence of maternal depressive symptoms was lower if mothers engaged in positive parenting behaviours.	Low
**Childhood (4 to 10 years old)**														
Graham & Easterbrooks (2000) ●	USA	Population	CS	85	46 %	7 to 9 years old	N/A	Maternal depressive symptoms (CES-D)	Moderator	Attachment security	Cumulative *F* = 9.78, *p* < 0.001	Depressive symptoms (DDPCA)	Attachment security and economic risk moderated parental depression’s effect on a child’s depressive symptoms.	Moderate
										Economic risk	Cumulative *F* = 12.49, *p* < 0.001			
Black et al. (2002) ◊	USA	Population	CS	194	50 %	4 to 5 years old	N/A	Maternal depressive symptoms (CES-D)	Moderator	Grandmother living in the household	*B* = –0.10, *p* > 0.05	Internalising symptoms (CBCL)	A grandmother living in the household did not moderate the association between maternal depressive symptoms and the child’s internalising or externalising behaviours, but there was evidence for a 3-way interaction between maltreatment, grandmother, and maternal depression.	Low
											*B* = 0.01, *p* > 0.05	Externalising symptoms (CBCL)		
Owens & Shaw (2003) *	USA	Population	PC	299	0 %	2 to 6 years old	0–0.5	Maternal depressive symptoms (BDI)	Moderator	Maternal acceptance (intercept)	coef = 0.0028 (0.0011), *p* < 0.05	Externalising symptoms (CBCL)	Maternal acceptance buffered maternal depressive symptoms association with externalising symptoms at age 6 (intercept). Offspring negative emotionality but not maternal acceptance buffered the association between maternal depression and offspring externalising symptoms between ages 2 and 6 (slope).	Moderate
										Maternal acceptance (slope)	coef = 0.0006 (0.0003), *p* > 0.05			
										Negative emotionality (offspring; intercept)	coef = 0.0002 (0.0005), *p* > 0.05			
										Negative emotionality (offspring; slope)	coef = 0.0003 (0.0001), *p* < 0.05			
Silk et al. (2006) ◊	USA	Population	CS	78	44 %	4 to 7 years old; 5.0 (1.2)	N/A	Maternal childhood-onset depression (FUISA and SCID-IV) Maternal depressive symptoms (BDI)	Moderator	Positive reward anticipation	*F* = 5.26, *p* < 0.05	Internalising symptoms (CBCL)	Positive reward anticipation moderated the relationship between maternal depression and the child’s internalising symptoms.	Low
											*F* = 4.22, *p* < 0.05			
Abela & Skitch (2007) *	Canada	High risk	PC	140	51 %	6 to 14 years old; 9.8 (2.3) at baseline, outcome 1 year later	1	Parental past or current MDD (SCID-IV)	Main effect	Self-esteem	*B* = 0.37, *p* > 0.05	Depressive symptoms (CDI)	There was no main effect for self-esteem in the context of a 3-way interaction between self-esteem, dysfunctional attitudes, and fluctuations in hassles; however, there was evidence for a 3-way interaction.	Low
Chang et al. (2007a) ◊	USA	Population	PC	6552	50 %	4 to 14 years old; 5.6 (3.1)	10	Maternal depressive symptoms (CES-D)	Moderator	Father’s positive involvement	β = 0.06, *p* = 0.02	Internalising problems (BPI)	The father’s involvement attenuated maternal depressive effects on the child’s internalising and externalising symptoms.	Low
											β = 0.06, *p* = 0.01	Externalising problems (BPI)		
Shannon et al. (2007) *	USA	Population	CS	180	33 %	8 to 12 years old; 9.9 (1.5)	N/A	Maternal melancholic depression (SCID-IV)	Moderator	Electrodermal responding	β = –0.31, *p* > 0.05	Conduct problems (CSI and CBCL)	Biological markers of temperament and emotionality did not moderate the association between maternal melancholia and parent-reported child conduct problems and depression.	Low
										Respiratory sinus arrhythmia	β = 0.21, *p* > 0.05			
										Preejection period	β = –0.04, *p* > 0.05			
										Electrodermal responding	β = 0.02, *p* > 0.05	Depressive symptoms (CSI and CBCL)		
										Respiratory sinus arrhythmia	β = 0.05, *p* > 0.05			
										Preejection period	β = 0.37, *p* > 0.05			
Turney (2011) ◊	USA	Population	PC	2427	48 %	5 years old; 64.2 (3.2) months old	5	Maternal MDD (CIDI-SF)	Moderator	Sex (male)	*B* = –0.53 (0.22), *p* < 0.05	Internalising symptoms (CBCL)	Maternal depression was more detrimental for young boys than for young girls.	Low
											*B* = –0.56 (0.22), *p* < 0.05	Externalising symptoms (CBCL)		
Abela et al. (2012) *	Canada	High risk	PC	140	51 %	6 to 14 years old: 9.8 (2.3) at baseline, outcome 1 year later	1	Parental past or current MDD (SCID-IV)	Main effect	Self-esteem (model 1)	*B* = 0.36, *p* > 0.05	Depressive symptoms (CDI)	Self-esteem was not protective in the offspring of depressed parents.	Low
										Self-esteem (model 2)	*B* = 0.23, *p* > 0.05			
Herba et al. (2013) ◊	Canada	Population	PC	1759	50 %	17 to 60 months old	1	Maternal depressive symptoms (CES-D)	Moderator	Early childcare age vs maternal care	OR = 0.24 (0.09, 0.66), *p* = 0.006	Emotional problems (CBCL, OCHS, and PBQ)	Early and late childcare had a protective influence for children of mothers with elevated depressive symptoms, reducing children’s risks for emotional problems and separation anxiety. Benefits were observed within the context of regulated group-based childcare for emotional problems and applied to early and late entry into childcare for both emotional problems and social withdrawal symptoms.	Low
										Late childcare age vs maternal care	OR = 0.29 (0.11, 0.77), *p* = 0.013			
										Early vs late entry	OR = 0.82 (0.45, 1.50), *p* = 0.52			
										Childcare by relative vs maternal care	OR = 0.53 (0.24, 1.16), *p* = 0.11			
										Group-based childcare vs maternal care	OR = 0.21 (0.09, 0.48), *p* = 0.002			
										Group-based childcare vs relative	OR = 0.40 (0.17, 0.94), *p* = 0.03			
										Increasing intensity of childcare use vs maternal care	OR = 0.65 (0.31, 1.38), *p* = 0.26			
										Full-time childcare use vs maternal care	OR = 0.58 (0.25, 1.33), *p* = 0.20			
										Full-time childcare vs increasing intensity of childcare use	OR = 0.89 (0.41, 1.94), *p* = 0.77			
										Early childcare age vs maternal care	OR = 0.29 (0.09, 0.92), *p* = 0.04	Separation anxiety (CBCL, OCHS, and PBQ)		
										Late childcare age vs maternal care	OR = 0.21 (0.07, 0.65), *p* = 0.007			
										Early vs late entry	OR= 1.39 (0.59, 3.25), *p* = 0.45			
										Childcare by relative vs maternal care	OR = 0.58 (0.21, 1.61), *p =* 0.30			
										Group-based childcare vs relative	OR = 0.21 (0.07, 0.67), *p* = 0.009			
										Group-based childcare vs relative	OR = 0.36 (0.11, 1.24), *p* = 0.11			
										Increasing intensity of childcare use vs maternal care	OR = 0.34 (0.11, 1.04), *p* = 0.06			
										Full-time childcare use vs maternal care	OR = 0.71 (0.26, 1.94), *p* = 0.51			
										Full-time childcare vs increasing intensity of childcare use	OR = 2.1 (0.68, 6.51), *p* = 0.20			
										Early childcare age vs maternal care	OR = 3.37 (0.89, 12.75), *p* = 0.07	Social withdrawal symptoms (CBCL, OCHS, and PBQ)		
										Late childcare age vs maternal care	OR = 2.8 (0.79, 9.96), *p* = 0.11			
										Early vs late entry	OR = 1.21 (0.63, 2.32), *p* = 0.58			
										Childcare by relative vs maternal care	OR = 1.35 (0.58, 3.16), *p* = 0.49			
										Group-based childcare vs maternal care	OR = 0.62 (0.26, 1.49), *p* = 0.29			
										Group-based childcare vs relative	OR = 0.46 (0.18, 1.16), *p* = 0.10			
										Increasing intensity of childcare use vs maternal care	OR = 0.96 (0.42, 2.21), *p* = 0.92			
										Full-time childcare use vs maternal care	OR = 1.18 (0.46, 3.00), *p* = 0.73			
										Full-time childcare vs increasing intensity of childcare use	OR = 1.23 (0.51, 2.94), *p* = 0.64			
Gere et al. (2013) ◊	Norway	Population	CS	190	38 %	7 to 13 years old; 10.4 (1.6)	N/A	Maternal depressive symptoms (HSCL-10)	Moderator	Father’s depressive symptoms	β = 0.29, *p* = 0.04	Depressive symptoms (mother-reported; CBCL)	When fathers reported few depressive symptoms for themselves, no association between mothers and children’s depressive symptoms were observed. The more depressive symptoms in fathers, the stronger the relationship between mothers and children’s symptoms.	Low
											β = 0.45, *p* < 0.01	Depressive symptoms (father-reported; CBCL) Depressive symptoms (child-reported; MFQ)		
											β = 0.06, *p* = 0.79	Depressive symptoms (child-reported; MFQ)		
Goelman et al. (2014) ◊	USA	Population	PC	294	54 %	6.5 years old	0–2	Parental depressive symptoms (CES-D)	Moderator	Childcare quality	*B =* –0.02, *p* < 0.05	Hostile-aggressive behaviour (PBQ) at 4.5 years	Childcare quality moderated the association between parental depressive symptoms and children’s hostile-aggressive behaviours in a way that children in high-quality childcare demonstrated less aggressive symptoms.	Low
											*B* = –0.02, *p* > 0.05	Anxious-fearful behaviour (PBQ) at 4.5 years		
											*B* = -0.01, *p* > 0.05	Hyperactive-distractible behaviour (PBQ) at 4.5 years		
											*B* = 0.00, *p* > 0.05	Internalising problems (HBQ) at 6.5 years		
											*B* = –0.01, *p* > 0.05	Externalising problems (HBQ) at 6.5 years		
Delany-Brumsey et al. (2014) ◊	USA	Population	CS	1305	N/R	5 to 11 years old; 7.9 (1.9)	N/A	Maternal depression probability (CIDI-SF)	Moderator	Social capital	*B* = –1.28, *p* > 0.05	Internalising problems (BPI)	Social capital did not buffer the association between maternal depression and a child’s internalising or externalising symptoms (but they found effects in adolescence).	Low
											*B* = 0.12, *p* > 0.05	Externalising problems (BPI)		
Fox & Borelli (2015) *	USA	High risk	CS	107	53 %	8 to 12 years old; 9.7 (1.5)	N/A	Maternal depressive symptoms (BDI)	Main effect	Attachment security	*B* = –0.012, (–0.074, 0.070), *p* > 0.05	Depressive symptoms (CDI)	Attachment security was not associated with child depressive symptoms but moderated the association between maternal and child depressive symptoms.	Moderate
Davis et al. (2016) ◊	USA	Population	CS	108	39 %	3.5 (0.5) years old	N/A	Maternal depressive symptoms (SCL-90)	Moderator	Child’s respiratory sinus arrhythmia (RSA) fear suppression	*B* = –1.11 (–2.13, –0.08), *p* < 0.05	Internalising and externalising symptoms (CBCL)	RSA suppression in response to the fear and happy but not sad clip moderated the positive association between maternal and child anxious/depressive symptoms, such that higher suppression served a protective-stabilising function and decreased children’s risk for internalising symptoms in the context of higher maternal symptoms.	Low
										*B* = 1.32 (0.20, 2.44), *p* < 0.05				
										*B* = 0.13 (–1.49, 1.74), *p* = 0.88				
Yan (2016) ◊	USA	Population	PC	1364	48 %	6 years old	2	Maternal depressive symptoms (CES-D)	Moderator	Self-assertion	*B =* 0.008 (0.005), *p* > 0.05	Internalising symptoms (CBCL and TRF)	Agentic processes (self-assertion, mastery motivation and effortful control) did not moderate the association between maternal depressive symptoms and the child’s internalising and externalising symptoms.	Low
										Mastery motivation	*B* = 0.013 (0.009), *p* > 0.05			
										Effortful control	*B* = –0.058 (0.059), *p* > 0.05			
										Self-assertion	*B* = 0.002 (0.008), *p* > 0.05	Externalising symptoms (CBCL and TRF)		
										Mastery motivation	*B* = 0.029 (0.016), *p* > 0.05			
										Effortful control	*B* = 0.042 (0.100), *p* > 0.05			
Goodlett et al. (2017) ◊	USA	Population	CS	106	47 %	Kindergarten age	N/A	Maternal depressive symptoms (BDI)	Moderator	Positive word use	B = –0.29, *p* < 0.01	Internalising symptoms (CBCL)	Positive emotion word use moderated the association between parental depressive symptoms and child internalizing problems.	Low
Charrois et al. (2017) ◊	Canada	Population	PC	264	51 %	4 years old	3	Maternal MDD (DIS and SCID)	Moderator	Childcare quality	β = – 0.185, *p* = 0.01	Externalising symptoms (PBQ)	In the context of postnatal maternal depression, high-quality child care was associated with fewer behavioural problems and may thus constitute a protective factor.	Low
											β = – 0.132, *p* = 0.13	Internalising symptoms (PBQ)		
											β = – 0.237, *p* < 0.001	Hyperactivity/inattention (PBQ)		
											β = – 0.176, *p* = 0.02	Opposition (PBQ)		
Andreas et al. (2017) *	Germany	Population	PC	170	54 %	6 to 8 years old; 7.3 (6.2–8.6)	2.13	Parental depressive symptoms (PHQ-9)	Moderator	Children’s positive representation of the maternal figure in girls	*B* = –1.01, *p* < 0.001	Depressive symptoms (CES-D)	Showing higher levels of positive representations of the maternal figure buffered the negative effect of maternal depressive symptoms for girls.	Moderate
										Children’s positive representation of the maternal figure in boys	*B* = –0.30, *p* > 0.05			
Gilbert et al (2017) ◊	USA	Population	CS	81	53 %	7 to 10 years old; 8.9 (1.2)	N/A	Maternal MDD (SCID-IV)	Moderator	Positive rumination	β = 0.3, *p* < 0.05	Depressive symptoms (CDI)	Contrary to the hypothesis, positive rumination was a risk factor and was associated with higher depressive symptoms in children at high risk.	Low
Giallo et al-(2018) *	Australia	High risk	PC	1085	48 %	From pregnancy to age 4	3.5-4	Maternal depressive symptoms (EPDS)	Main effect	Sex (female)	OR = 0.91 (0.52, 0.61), *p* = 0.753	Emotional-behavioural functioning (resilience; SDQ)	Maternal age, tertiary education and higher income were associated with emotional and behavioural resilience.	Low
										Maternal age	OR = 1.12 (1.06, 1.20), *p* < 0.001			
										Maternal tertiary education (during pregnancy)	OR = 3.58 (1.94, 6.60), *p* < 0.001		Support from a partner six months postpartum and maternal involvement in home activities such as reading or talking with their child at four years was also protective.	
										Higher income	OR = 1.9 (1.05, 3.44), *p* = 0.033			
										Time to self at least once per week (at 6 months)	OR = 0.61 (0.27, 1.40), *p* = 0.246			
										Emotional satisfaction in a relationship (at 6 months)	OR = 1.77 (0.96, 3.24), *p* = 0.066			
										High support from partner (at 6 months)	OR = 3.17 (1.26, 8.00), *p* = 0.015			
										High partner contribution in caregiving (at 6 months)	OR = 1.19 (0.52, 2.72), *p* = 0.687			
										>1 child in the household (at 4 years)	OR = 0.73 (0.41, 1.32), *p* = 0.297			
										> 1 adult in the household (at 4 years)	OR = 1.76 (0.83, 3.73), *p* = 0.142			
										Emotional satisfaction in a relationship (at 4 years)	OR = 1.32 (0.75, 2.33), *p* = 0.339			
										No relationship transitions (at 4 years)	OR = 1.44 (0.81, 2.56), *p* = 0.218			
										High partner contribution in caregiving (at 4 years)	OR = 0.42 (0.55, 13.00), *p* = 0.421			
										Time to self at least once per week (at 4 years)	OR = 1.18 (0.66, 2.11), *p* = 0.571			
										Maternal involvement in home activities (at 4 years)	OR = 1.14 (1.03, 1.26), *p* = 0.008			
Vakrat et al. (2018) *	Isreal	Population	CS	1983	49 %	6 years old; 6.3 (1.3)	N/A	Maternal MDD (SCID-IV)	Moderator	Paternal sensitivity	OR = 0.58 (0.36, 0.95), *p* < 0.05	Psychiatric diagnosis (DAWBA)	Sensitive fathering was associated with lower odds of developing a psychiatric disorder, and the protective effect of the father was specific to the depressed group, not to controls.	Low
Braithwaite et al. (2020) *	UK	Population	PC	8354	45 %	3.5 years old	2–5	Maternal depressive symptoms (EPDS)	Moderator	Postnatal depression x prenatal depression x sex	coef = 0.042 (0.015, 0.068), *p* = 0.002	Emotional symptoms (RPTS)	The association between postnatal maternal depressive symptoms and child emotional symptoms was moderated by the level of prenatal depressive symptoms in a sex-dependent manner. In boys, the association between postnatal depression and child emotional symptoms was weaker following lower prenatal depressive symptoms.	Low
										Prenatal depression in boys	coef = 0.030 (0.012, 0.048), *p* = 0.001			
										Prenatal depression in girls	coef = –0.012 (–0.031, 0.007), *p* = 0.221			
Taraban et al. (2020)	USA	Population	PC	166	51 %	4 years old	1	Paternal depressive symptoms (CES-D)	Moderator	Interparental relationship quality	*B* = – 0.02 (0.01), *p* < 0.05	Internalising symptoms (CBCL)	Both interparental relationship quality and child inhibitory control attenuated the association between paternal depressive symptoms and a child’s internalizing symptoms.	Low
										Child inhibitory control	*B* = – 0.25 (0.10), *p* < 0.05			
West et al. (2020) ◊	USA	Population	CS	97	54 %	9 to 12 years old; 10.3 (1.2)	N/A	Maternal depressive symptoms (BDI)	Moderator	Dyadic positivity	β = –1.25, *p* < 0.001	Internalising and externalising symptoms (CBCL)	High levels of positivity, engagement and negative physiological synchrony buffered the association between maternal depressive symptoms and child internalising and externalising symptoms	Low
										Dyadic engagement	β = –1.5, *p* < 0.001			
										Physiological synchrony	β = 0.39, *p* < 0.01			
Carlone & Milan (2021) ◊	USA	Population	PC	1917	N/R	9.3 years old	6	Maternal MDD (CIDI-SF)	Moderator	Attachment quality	*F* = 5.67, *p* < 0.01	Externalising symptoms (mother-reported; CBCL)	Secure attachment buffered maternal depression association with mother-, child-, and teacher-reported externalising symptoms.	Low
										Attachment quality	*F* = 9.72, *p* < 0.01	Externalising symptoms (child-reported; TTYHD)		
										Attachment quality	*F* = 5.17, *p* < 0.05	Externalising symptoms (teacher-reported; CTRS-R:S)		
**Adolescence (11 to 17 years old)**														
Conrad & Hammen (1993) *	USA	Population	PC	96	52 %	8 to 16 years old: 12.5 (2.5) at baseline, outcome 3 years later	3	Parental MDD (SADS-LA)	Moderator	Self-esteem	r^2^ = 0.02, *p* > 0.05	Psychiatric diagnosis (K-SADS)	Children of unipolar women benefitted more from social competence than children not experiencing such risk. Having mothers at home rather than externally employed had a stronger protective effect on the offspring of the mothers with unipolar depression and other chronic medical conditions compared to the well mothers (authors’ conclusion).	Moderate
										Academic performance	r^2^ = 0.00, *p* > 0.05			
										Child social competence	r^2^ = 0.04, *p* = 0.051			
										Child’s positive perception of a mother	r^2^ = 0.01, *p* > 0.05			
										Maternal employment	r^2^ = 0.05, *p* = 0.073			
										Maternal social competence	r^2^ = 0.00, *p* > 0.05			
										Healthy dad	r^2^ = 0.00, *p* > 0.05			
										Children’s friendships	r^2^ = 0.00, *p* > 0.05			
										Adult friend	r^2^ = 0.01, *p* > 0.05			
Brennan et al. (2003) ◊	Australia	Population	CS	816	49 %	15.2 (0.3) years old	N/A	Maternal MDD (SCID-IV)	Moderator	Father diagnosis absent	β = –0.19, *p* = 0.57	Mental health resilience (K-SADS, CBCL, and interviews)	Low levels of parental psychological control, high levels of maternal warmth, and low levels of maternal overinvolvement all interacted with maternal depression to predict resilient outcomes in youth.	Low
										Father firm control	β = 0.02, *p* = 0.68			
										Father psychological control	β = −0.10, *p* = 0.03			
										Father acceptance	β = 0.03, *p* = 0.46			
										Father criticism	β = −0.56, *p* = 0.20			
										Father emotional overinvolvement	β = 0.24, *p* = 0.54			
										Mother warmth	β = 0.02, *p* = 0.02			
										Mother firm control	β = −0.02, *p* = 0.6			
										Mother psychological control	β = − 0.11, *p* = 0.008			
										Mother acceptance	β = 0.07, *p* = 0.06			
										Mother criticism	β = −0.07, *p* = 0.77			
										Mother emotional overinvolvement	β = −0.59, *p* = 0.03			
Casey-Cannon et al. (2006) ◊	USA	Population	PC	290	44 %	12 to 15 years old: 14.0 (1.1) at baseline, outcome 1 year later	1	Maternal depressive symptoms (BDI)	Moderator	Non-parent adult support	β = 0.02, *p* > 0.05	Depressive symptoms (N/R)	Non-parent adult support did not moderate the association between parental depression and adolescent’s depressive symptoms. There were no gender-specific effects either.	Moderate
										Non-parent adult support x sex	β = −0.27, *p* > 0.05			
								Paternal depressive symptoms (BDI)		Non-parent adult support	β = −0.06, *p* > 0.05			
										Non-parent adult support x sex	β = 0.01, *p* > 0.05			
Bohnert & Garber (2007) ◊	USA	Population	CS	198	57 %	11.9 (0.6) years old at baseline, outcome 6 years later	N/A	Maternal MDD (SCID-IV)	Moderator	Involvement in school and community-based activities	*B* = −0.86, *p* > 0.05	Externalising symptoms (CBCL)	The association between maternal depression and adolescent psychopathology was not buffered by adolescent involvement in school and community-based activities.	Low
											*B* = −0.60, *p* > 0.05	Internalising symptoms (CBCL)		
											OR = 1.00, *p* > 0.05	Mood disorders (K-SADS)		
											OR = 1.03, *p* > 0.05	Anxiety disorders (K-SADS)		
											OR = 0.97, *p* > 0.05	Behavioural disorder (K-SADS)		
Chang et al. (2007b) ◊	USA	Population	CS	122	50 %	10 to 12 years old; 11.0 (0.1)	N/A	Maternal depressive symptoms (BDI)	Moderator	Child’s resourcefulness	β = −0.03, *p* > 0.05	Depressive symptoms (CDI)	Resourcefulness did not moderate the association between maternal depression and adolescent’s depressive symptoms.	Low
Cummings et al. (2007) ◊	USA	Population	PC	157	45 %	6 to 12 years old: 11.5 (2.0) at baseline, outcome 2 years later	2	Maternal depressive symptoms (SCL-90)	Moderator	SCLR to inter-adult argument	T ratio = 0.15, *p* > 0.05	Internalising problems (CBCL)	SCLR moderated the association between parental depression and child internalising and externalising symptoms, especially for paternal depression. Higher SCLR predicted greater vulnerability to paternal depression.	Low
										SCLR to star-tracing	T ratio = 0.02, *p* > 0.05			
								Paternal depressive symptoms (SCL-90)		SCLR to inter-adult argument	T ratio = 0.67, *p* < 0.01			
										SCLR to star-tracing	T ratio = 0.19, *p* < 0.05			
								Maternal depressive symptoms (SCL-90)		SCLR to inter-adult argument	T ratio = 0.33, *p* < 0.05	Internalising problems (PIC)		
										SCLR to star-tracing	T ratio = 0.04, *p* > 0.05			
								Paternal depressive symptoms (SCL-90)		SCLR to inter-adult argument	T ratio = 0.49, *p* < 0.05			
										SCLR to star-tracing	T ratio = 0.12, *p* > 0.05			
								Maternal depressive symptoms (SCL-90)		SCLR to inter-adult argument	T ratio = 0.08, *p* > 0.05	Externalising problems (CBCL)		
										SCLR to star-tracing	T ratio = 0.01, *p* > 0.05			
								Paternal depressive symptoms (SCL-90)		SCLR to inter-adult argument	T ratio = 0.40, *p* < 0.05			
										SCLR to star-tracing	T ratio = 0.14, *p* < 0.1			
								Maternal depressive symptoms (SCL-90)		SCLR to inter-adult argument	T ratio = 0.20, *p* > 0.05	Externalising problems (PIC)		
										SCLR to star-tracing	T ratio = 0.04, *p* > 0.05			
								Paternal depressive symptoms (SCL-90)		SCLR to inter-adult argument	T ratio = −0.08, *p* > 0.05			
										SCLR to star-tracing	T ratio = 0.19, *p* < 0.05			
Boyd et al. (2008) ◊	USA	High risk	CS	63	59 %	7 to 14 years old; 11.2 (2.1)	N/A	Current or past maternal MDD, dysthymic or depressive disorder (diagnostic interview - N/R)	Main effect	Social skills	*B* = 1.11, *p* = 0.01	Anxiety symptoms (coping; MASC)	The findings demonstrated partial support for social skills affecting anxiety outcomes in children of depressed mothers who were exposed to community violence.	Low
											*B* = −0.03, *p* = 0.93	Anxiety symptoms (physical symptoms; MASC)		
Milan et al. (2009) ◊	USA	Population	PC	938	N/R	11 years old	8	Maternal depressive symptoms (CES-D)	Moderator	Attachment security	*F* = 5.20, *p* = 0.006	Depressive symptoms (CDI)	Preschool attachment quality moderated the relationship between maternal and adolescent depressive symptoms. Maternal depressive symptoms predicted offspring depressive symptoms only among those children with an insecure attachment.	Low
Woodhouse et al. (2010) ◊	USA	Population	CS	189	62 %	11th graders	N/A	Maternal depressive symptoms (CES-D)	Moderator	Attachment security	*B* = 0.71, *p* = 0.11	Depressive symptoms (CDI)	Adolescent attachment security moderated the association between paternal depression and adolescent depressive symptoms, with secure attachment playing a protective role.	Low
								Paternal depressive symptoms (CES-D)		Attachment security	*B* = 0.5, *p* = 0.08			
Jacobs et al. (2012) *	USA	High risk	CS	78	N/R	12.0 (5.8) years old	N/A	Maternal MDD (SADS-LA)	Main effect	Concordance of religion importance	OR = 0.44 (0.09, 2.13), *p* > 0.05	Anxiety or depression (K-SADS	Concordance of denomination was associated with lower odds of childhood anxiety or depression.	Moderate
										Concordance of religion attendance	OR = 0.43 (0.09, 1.99), *p* > 0.05			
										Concordance of religion denomination	OR = 0.09 (0.02, 0.54), *p* < 0.01			
Hooper et al. (2012) *	USA	Population	CS	51	51 %	12 to 17 years old; 13.8 (1.3)	N/A	Parental depressive symptoms (BDI)	Moderator	Parentification	β = 0.01, *p* = 0.986	Depressive symptoms (BDI)	Parentification was not a moderator of the association between parent depressive symptoms and adolescent depressive symptoms.	Low
Boyd & Waanders (2013) ◊	USA	High risk	CS	77	58 %	8 to 14 years old; 11.1 (2.0)	N/A	Current or past maternal depressive disorder (SCID-IV and BDI)	Main effect	Parenting skills (child-reported)	*B* = −2.14 (1.08), *p* = 0.051	Depressive symptoms (CDI)	Some evidence for protective role of parenting skills and child’s social skills but not maternal kidship support in the context of the two-way interaction between parenting and child’s social skills (author’s conclusion).	Low
										Child social skills (child-reported)	*B* = −0.12 (0.06), *p* = 0.059			
										Maternal kinship support (child-reported)	*B* = −0.46 (1.21), *p* = 0.703			
										Parenting skills (mother-reported)	*B* = 1.66 (1.15), *p* = 0.153			
										Child social skills (mother-reported)	*B* = −0.15 (0.07), *p* = 0.047			
										Maternal kinship support (mother-reported)	*B* = 0.23 (1.08), *p* = 0.832			
Chen (2013) ◊	USA	High risk	CS	126	51 %	12 to 14 years old	N/A	Parental lifetime diagnosis of depression (UM-CIDI)	Main effect	Self-control	*B* = −0.111, *p* > 0.05	Emotional adjustment (DISC-IV)	Optimism had a protective effect on emotional outcomes while self-control, parental monitoring and prosocial friends - on behavioural.	Low
										Optimism	*B* = −0.215, *p* < 0.05			
										Parent-child relationships (warmth)	*B* = −0.099, *p* > 0.05			
										Parent monitoring	*B* = 0.093, *p* > 0.05			
										Prosocial friends	*B* = 0.015, *p* > 0.05			
										Teacher support	*B* = 0.153, *p* 0.05			
										Self-control	*B* = −0.21, *p* < 0.05	Behavioural adjustment (DISC-IV)		
										Optimism	*B* = 0.113, *p* > 0.05			
										Parent-child relationships (warmth)	*B* = − 0.017, *p* > 0.05			
										Parent monitoring	*B* = −0.25, *p* < 0.05			
										Prosocial friends	*B* = − 0.187, *p* > 0.05			
										Teacher support	*B* = 0.111, *p* < 0.05			
Harold et al. (2014) *	UK	High risk	PC	145	100 %	14.0 (1.5) years old	0 to 2.25	Maternal MDD (SCAN)	Main effect	Maternal caregiving involvement (intercept)	coef = −0.006, *p* = 0.787	Depressive symptoms (CAPA)	Girls who had mothers with recurrent depression showed reduced antisocial behaviour when their mothers were highly involved.	Low
										Maternal caregiving involvement (slope)	coef = 0.007, *p* = 0.675			
										Maternal caregiving involvement (intercept)	coef = −0.073, *p* = 0.019	Antisocial behaviour (CAPA)		
										Maternal caregiving involvement (slope)	coef = 0.011, *p* = 0.58			
Delany-Brumsey et al. (2014) ◊	USA	Population	CS	1305	N/R	12 to 17 years old; 14.4 (1.7)	N/A	Probability of maternal depression (CIDI-SF)	Moderator	Social capital	*B* = −4.23, *p* < 0.01	Internalising problems (BPI)	For adolescents who lived in high social capital neighbourhoods, the association between maternal depression and behaviour problems was attenuated (but did not find effects in childhood).	Low
											*B* = −5.08, *p* < 0.05	Externalising problems (BPI)		
Sun et al. (2015) ◊	China	Population	CS	1419	49 %	15.4 (1.8) years old	N/A	Parental depressive symptoms (CES-D)	Moderator	Resilience	β = 0.01, *p* > 0.05	Internalising problems (YSR)	Resilience did not moderate the association between parental depression and the child’s internalising or externalising symptoms.	Low
											β = 0.01, *p* > 0.05	Externalising problems (YSR)		
Davidovich et al. (2016) ◊	UK	High risk	CS	288	60 %	9 to 17 years old; 13.8 (2.0)	N/A	Parental MDD (SCAN)	Main effect	Inhibitory control	β = 0.15, *p* = 0.05	Depressive symptoms (CAPA)	Inhibitory control (more errors) was associated with depressive symptoms in the context of the significant interaction between current parental depression and inhibitory control.	Low
										Mental flexibility	β = 0.02, *p* = 0.75			
										Set-shifting	β = −0.09, *p* = 0.23			
Collishaw et al. (2016) ◊	UK	High risk	PC	262	60 %	9 to 17 years old: 12.3 (2.1) at baseline; outcome 3 years later	3	Parental MDD disorder (SCAN)	Main effect	Parent warmth	OR = 1.19 (0.84, 1.69), *p* = 0.34	Sustained good mental health (CAPA)	Index parent positive expressed emotion, co-parent support, good-quality social relationships, self-efficacy, and frequent exercise were associated with sustained good mental health. Analyses accounting for parent depression severity were consistent, but frequent exercise only predicted better than expected mood-related mental health, not behavioural mental health, whereas index parents’ expression of positive emotions predicted better than expected behavioural mental health, not mood-related mental health.	Low
										Parent positive expressed emotion	OR = 1.91 (1.31, 2.79), *p* = 0.0008			
										Co-parent support	OR = 1.90 (1.38, 2.62), *p* = 0.0001			
										Sibling warmth	OR = 1.14 (0.80, 1.61), *p* = 0.48			
										Parent-reported peer relationship quality	OR = 2.07 (1.35, 3.18), *p* = 0.001			
										Adolescent-reported peer relationship quality	OR = 1.36 (0.96, 1.93), *p* = 0.08			
										Out of school activities	OR = 1.41 (0.74, 2.71), *p* = 0.30			
										Adolescent perceived friendships	OR = 1.30 (0.94, 1.81), *p* = 0.12			
										Self-efficacy	OR = 1.49 (1.05, 2.11), *p* = 0.03			
										Physical exercise	OR = 2.96 (1.26, 6.92), *p* = 0.01			
										Parent warmth	β = −0.06, *p* = 0.33	Mood resilience (CAPA)		
										Parent positive expressed emotion	β = −0.11, *p* = 0.08			
										Co-parent support	β = −0.23, *p* = 0.0001			
										Sibling warmth	β = 0.06, *p* = 0.43			
										Parent-reported peer relationship quality	β = −0.17, *p* = 0.006			
										Adolescent-reported peer relationship quality	β = −0.17, *p* = 0.005			
										Out of school activities	β = −0.15, *p* = 0.02			
										Adolescent perceived friendships	β = −0.13, *p* = 0.03			
										Self-efficacy	β = −0.22, *p* = 0.001			
										Physical exercise	β = −0.22, *p* = 0.0004			
										Parent warmth	β = −0.17, *p* = 0.007	Behavioural resilience (CAPA)		
										Parent positive expressed emotion	β = −0.16, *p* = 0.01			
										Co-parent support	β = −0.14, *p* = 0.03			
										Sibling warmth	β = −0.1, *p* = 0.15			
										Parent-reported peer relationship quality	β = −0.23, *p* = 0.0002			
										Adolescent-reported peer relationship quality	β = −0.16, *p* = 0.01			
										Out of school activities	β = −0.1, *p* = 0.12			
										Adolescent perceived friendships	β = −0.15, *p* = 0.02			
										Self-efficacy	β = −0.25, *p* = 0.0001			
										Physical exercise	β = − 0.001, *p* = 0.99			
Monti & Rudolph (2017) ◊	USA	Population	PC	165	52%	12.4 (1.2) years old ah baseline, outcome 4 years later	4	Maternal MDD (SCID-IV)	Moderator	Effortful engagement x gender	coef = 0.20 (0.08), *p* < 0.05	Depression (K-SADS)	Adaptive responses to stress (high effortful engagement and low involuntary disengagement) buffered the effect of maternal depression on initial levels and trajectories of youth depression, with gender differences emerging. Girls of depressed mothers who showed adaptive responses displayed essentially no initial depressive symptoms, while boys who used adaptive stress response also declined in depression as they progressed through adolescence.	Low
										Effortful engagement in girls	coef = 0.02 (0.05), *p* > 0.05			
										Effortful engagement in boys	coef = – 0.18 (0.07), *p<* 0.05			
										Involuntary disengagement x gender	coef = – 0.33 (0.13), *p* < 0.05			
										Involuntary disengagement in girls	coef = – 0.07 (0.05), *p* > 0.05			
										Involuntary disengagement in boys	coef = 0.26 (0.12), *p* < 0.05			
Mahedy et al. (2018) ◊	UK	High risk	PC	265	N/R	14.8 (2.0) years old	2.25	Parental lifetime MDD (N/R)	Main effect	Paternal emotional support	β = −0.21 (– 0.34, −0.06), *p* < 0.001	Mood resilience (CAPA)	High paternal emotional support was associated with fewer depressive symptoms and reduced likelihood of psychiatric disorder, but not with fewer disruptive behaviours.	Low
											β = −0.13 (– 0.28, 0.01), *p* = 0.07	Behavioural resilience (CAPA)		
											OR = 0.68 (0.56, 0.83), *p* < 0.001	DSM disorder (CAPA)		
Manczak et al. (2018) ◊	USA	Population	PC	194	100 %	12 to 16 years old: 14.5(1.2) at baseline, outcome 1 year later	1	Maternal depressive symptoms (SCL-90)	Moderator	High quality mother-daughter communication	*B* = −0.11, (0.00), *p* = 0.009	Externalising problems (YSR)	Interaction between the quality of communication and maternal depressive symptoms on externalising and internalising symptoms in daughters, such that the risk associated with maternal depressive symptoms was fully buffered for daughters in high quality communication dyads.	Low
											*B* = −0.01, (0.00), *p* = 0.032	Internalising problems (YSR)		
Kujawa et al. (2019) ◊	USA	Population	PC	369	44 %	12.7 (0.4) years old	3	Maternal depression (SCID-IV)	Moderator	Reward positivity	*B* = −0.12, *p* < 0.05	Depressive symptoms (CDI)	Reduced reward positivity and response, as measured by neurophysiology and self-report measures moderate the effects of maternal depression but not paternal effects on depressive symptoms in offspring.	Low
										Reward responsiveness	*B* = −0.38, *p*< 0.05			
								Paternal depression (SCID-IV)		Reward positivity	*B* = 0.03, *p* > 0.05			
										Reward responsiveness	*B* = −0.17, *p* > 0.05			
Vreeland et al. (2019) ◊	USA	Population	CS	117	45 %	9 to 15years old; 12.3 (1.9)	N/A	Maternal depressive symptoms (BDI)	Moderator	Primary control coping	β = −0.27, *p* < 0.001	Internalising problems (CBCL, YSR)	Interaction between primary and secondary control and maternal depression symptoms, with a weaker association between maternal depression symptoms and youth’s internalising and externalising symptoms for those with higher levels of either type of coping.	Low
										Secondary control coping	β = −0.20, *p* < 0.01			
										Primary control coping	β = −0.15, *p* > 0.05	Externalising problems (CBCL, YSR)		
										Secondary control coping	β = −0.26, *p* < 0.01			
**Young adulthood (18 to 25 years old)**														
Pargas et al. (2010) *	Australia	Population	PC	648	52 %	20 years old	5	Maternal depressive disorder (SCID-IV)	Moderator	Maternal acceptance	OR= 1.05 (0.98, 1.130, *p* = 0.20	Mental health resilience (YASR, SCID, K-SADS, LSI)	Low levels of perceived maternal psychological control and high child IQ acted as protective factors in the context of maternal depression.	Low
										Maternal firm control	OR = 0.95 (0.87, 1.03), *p* = 0.23			
										Maternal psychological control	OR = 0.90 (0.83, 0.98), *p* = 0.02			
										Maternal warmth	OR = 1.02 (0.99, 1.030, *p* = 0.09			
										Paternal acceptance	OR = 1.03 (0.97, 1.10), *p* = 0.31			
										Paternal firm control	OR = 1.04 (0.95, 1.13), *p* = 0.38			
										Paternal psychological control	OR = 1.01 (0.93, 1.10), *p* = 0.79			
										IQ	OR = 1.11 (1.03, 1.19), *p* < 0.01			
										Self-esteem	OR = 1.09 (0.98, 1.21), *p* = 0.12			
										Peer social functioning	OR = 1.15 (0.55, 2.39), *p* = 0.72			
Chang & Fu (2020) ◊	Taiwan	Population	PC	2502	49 %	13 to 23 years old	9	Maternal depressive symptoms (SCL-90)	Moderator	Self-esteem (time-stable)	*B* = –0.06, *p* < 0.05	Depressive symptoms (SCL-90-R)	Self-esteem buffered maternal but not paternal time-stable depression effects on a child’s depressive symptoms.	Low
										Self-esteem (time-varying)	*B* = 0.04, *p* > 0.05			
								Paternal depressive symptoms (SCL-90)		Self-esteem (time-stable)	*B* = 0.1, *p* > 0.05			
										Self-esteem (time-varying)	*B* = 0.15, *p* > 0.05			
**Adulthood (older than 25 years old)**														
Kasen et al. (2012) ^†^	USA	High risk	PC	185	60 %	29.5 (6.3) years old at 10 years FU, outcome 10 years later	10	Parental MDD (recruited from outpatient clinics, RDC)	Main effect	Religious attendance	OR = 0.82, (0.47, 1.43), *p* > 0.05	MDD (SADS-LA)	Did not find evidence for the protective effects of religious attendance or importance in the offspring of depressed parents.	Low
										Religious importance	OR = 0.86, (0.58, 1.28), *p* > 0.05			
										Religious attendance	OR = 0.94, (0.58, 1.53), *p* > 0.05	Mood disorder (SADS-LA)		
										Religious importance	OR = 0.99, (0.65, 1.52), *p* > 0.05			
										Religious attendance	OR = 0.94, (0.61, 1.45), *p* > 0.05	Any psychiatric disorder (SADS-LA)		
										Religious importance	OR = 0.92, (0.60, 1.42), *p* > 0.05			
Miller et al. (2012) ^†^	USA	High risk	PC	114	61 %	29.3 (5.5) years old at 10 years FU, outcome 10 years later	10	Parental MDD (recruited from outpatient clinics, RDC)	Main effect	Religion/spirituality highly important	OR = 0.09 (0.01, 0.82), *p* = 0.03	MDD (SADS-LA)	The importance of religion/spirituality, but not religious attendance or denomination, was protective against MDD diagnosis in adulthood in the offspring of depressed parents.	Low
										Frequent attendance at religious/spiritual services	OR = 0.49, (0.16, 1.55), *p* = 0.23			
										Catholic vs Protestant	OR = 1.37, (0.32, 5.88), *p* = 0.68			
Barton et al. (2013) ^†^	USA	High risk	PC	118	61 %	29.5 (6.3) years old at 10 years FU, outcome 10 years later	10	Parental MDD (recruited from outpatient clinics, RDC)	Main effect	Frequent attendance at religious/spiritual services	OR = 0.55 (0.19, 1.61), *p* = 0.276	MDD (SADS-LA)	Social adjustment but not frequent attendance at religious services was protective against MDD diagnosis in adulthood in the offspring of depressed parents.	Low
										High social adjustment	OR = 0.22, (0.08, 0.60), *p* = 0.004			
Lewandowski et al. (2014) ^†^	USA	High risk	PC	115	58 %	16.8 (5.03) years old at baseline, outcome assessed 2, 10, and 20 years later	20	Parental lifetime history of MDD (SADS-LA)	Main effect	Maternal affection	OR = 1.44 (0.94, 2.20), *p* = 0.096	Mental health resilience (absence of psychiatric diagnosis) (K-SADS or SADS-LA)	Offspring self-esteem was associated with resilience regardless of the definition of resilience. Additionally, easier offspring temperament was associated with resilience, defined as an absence of psychopathology, while lower maternal overprotection and higher offspring IQ were associated with resilience, defined as constant high functioning.	Low
										Maternal overprotection	OR = 0.94 (0.59, 1.50), *p* = 0.798			
										Parental marital adjustment	OR = 0.82 (0.55, 1.22), *p* = 0.317			
										Family cohesion	OR = 1.31 (0.79, 2.17), *p* = 0.286			
										Offspring easy temperament	OR= 1.86 (1.08, 3.18), *p* = 0.024			
										Offspring self-esteem	OR = 1.96 (1.19, 3.25), *p* = 0.009			
										Offspring IQ	OR = 1.26 (0.81, 1.96), *p* = 0.312			
										Maternal affection	OR = 1.93 (0.78, 4.74), *p* = 0.154	Mental health resilience (consistent high functioning) (C-GAS or GAS)		
										Maternal overprotection	OR = 0.27 (0.10, 0.73), *p* = 0.010			
										Parental marital adjustment	OR = 1.34 (0.70, 2.55), *p* = 0.367			
										Family cohesion	OR = 0.93 (0.54, 1.60), *p* = 0.803			
										Offspring easy temperament	OR = 1.76 (0.89, 3.50), *p* = 0.104			
										Offspring self-esteem	OR = 3.43 (1.63, 7.21), *p* = 0.001			
										Offspring IQ	OR = 1.64 (1.02, 2.63), *p* = 0.039			
Havinga et al. (2017) ^†^	Netherlands	High risk	PC	523	57 %	23 to 37 years old; 28.5 (3.1)	10	Parental lifetime diagnoses of depressive and anxiety disorder (received treatment and CIDI)	Main effect	Balanced family functioning	HR = 0.72, (0.55, 0.94), *p* = 0.016	Mood and anxiety disorders (CIDI)	Sex (being a female) and balanced family functioning but not IQ were found to be protective in adult offspring of depressed and anxious parents.	Low
										Sex (female)	HR = 2.20, (1.65, 2.95), *p* < 0.001			
										IQ	HR = 1.01, (1.00, 1.02), *p* = 0.118			

* unadjusted;

◊ adjusted for confounders;.

● with other covariates included;.

† adjusted univariable model (although reported both univariable and multivariable); N – number of participants; M – mean; SD – standard deviation; CI – confidence interval; FU – follow up; SE – standard error; USA – United States of America; UK – United Kingdom; PC – prospective cohort; CS – cross-sectional; MDD – major depressive disorder; CES-D – Centre for Epidemiologic Studies Depression Scale; BDI – Beck’s Depression Inventory; MI – Malaise Inventory; SCL-90-R – The Symptom Checklist-90-Revised; FUISA – Follow-Up Interview Schedule for Adults; SCID-IV – The Structured Clinical Interview for DSM–IV Axis I Disorders; CIDI-SF – Composite International Diagnostic Interview short-form; HSCL-10 – The Hopkins Symptom Checklist; DIS – Diagnostic Interview Schedule; PHQ-9 – Patient Health Questionnaire-9; EPDS – Edinburgh Postnatal Depression Scale; SADS-LA – Lifetime Version of the Schedule for Affective Disorders and Schizophrenia; UM-CIDI – University of Michigan Composite International Diagnostic Instrument; SCAN – The Schedule for Clinical Assessment; RDC – The Research Diagnostic Criteria; RSA – respiratory sinus arrhythmia; SCLR - skin conductance level reactivity; IQ – intelligence quotient; CBCL – The Child Behaviour Checklist; SDQ – The Strengths and Difficulties Questionnaire; DDPCA – Dimensions of Depression Profile for Children and Adolescents; CDI – The Children’s Depression Inventory; BPI – Behavioural Problems Index; CSI – Child Symptom Inventory; OCHS – Ontario Child Health Study Scales; PBQ – Preschool Behaviour Questionnaire; MFQ – The Mood and Feelings Questionnaire; TRF – Teacher Report Form; DAWBA – Development and Well-Being Assessment; RPTS – Revised Rutter Scale for Preschool Children; TTYHD – The Things That You Have Done Scale; CTRS-R:S – Conners’ Teacher Rating Scale—Revised Short Form; K-SADS – Kiddie Schedule for Affective Disorders and Schizophrenia; PIC – Personality Inventory for Children; MASC – The Multidimensional Anxiety Scale for Children; CAPA – The Child and Adolescent Psychiatric Assessment; YSR – Youth Self Report; YASR – The Young Adult Self-Report; LSI – Life Stress Interview; GAS – Global Assessment Scale.

**Table 2:** Strength of evidence for each protective factor.

Protective factor	Number of models that found evidence	Number of models that did not find evidence	% of models that found evidence	Developmental stage	Outcome	Test of protective effect
**Demographic characteristics**						
Household income (Giallo et al., 2018; Graham and Easterbrooks, 2000)	2	0	100 %	Childhood (Giallo et al., 2018; Graham and Easterbrooks, 2000)	Emotional-behavioural functioning (resilience) (Giallo et al., 2018) Depressive symptoms (Graham and Easterbrooks, 2000)	Main effect (Giallo et al., 2018) Moderator (Graham and Easterbrooks, 2000)
More than one child in the household (Giallo et al., 2018)	0	1	0 %	Childhood (Giallo et al., 2018)	Emotional-behavioural functioning (resilience) (Giallo et al., 2018)	Main effect (Giallo et al., 2018)
More than one adult in the household (Giallo et al., 2018)	0	1	0 %	Childhood (Giallo et al., 2018)	Emotional-behavioural functioning (resilience) (Giallo et al., 2018)	Main effect (Giallo et al., 2018)
Maternal age (Giallo et al., 2018)	1	0	100 %	Childhood (Giallo et al., 2018)	Emotional-behavioural functioning (resilience) (Giallo et al., 2018)	Main effect (Giallo et al., 2018)
Maternal tertiary education ( Giallo et al., 2018)	1	0	100 %	Childhood (Giallo et al., 2018)	Emotional-behavioural functioning (resilience) (Giallo et al., 2018)	Main effect (Giallo et al., 2018)
Maternal employment (Conrad and Hammen, 1993)	0	1	0 %	Adolescence (Conrad and Hammen, 1993)	Psychiatric diagnosis (Conrad and Hammen, 1993)	Moderator (Conrad and Hammen, 1993)
Sex (Giallo et al., 2018; Havinga et al., 2017; Lee et al., 2006; Turney, 2011)	3	3	50 %	Toddlerhood (Lee et al., 2006) Childhood (Giallo et al., 2018; Turney, 2011) Adulthood (Havinga et al., 2017)	Externalising symptoms (Lee et al., 2006; Turney, 2011) Internalising symptoms (Lee et al., 2006; Turney, 2011) Emotional-behavioural functioning (resilience) (Giallo et al., 2018) Mood and anxiety disorders (Havinga et al., 2017)	Moderator (Lee et al., 2006; Turney, 2011) Main effect (Giallo et al., 2018; Havinga et al., 2017)
Ethnicity (Feldman and Masalha, 2007)	1	0	100 %	Toddlerhood (Feldman and Masalha, 2007)	Internalising and externalising symptoms (Feldman and Masalha, 2007)	Moderator (Feldman and Masalha, 2007)
**Family factors**						
Grandmother living in the household (Black et al., 2002)	0	2	0 %	Childhood (Black et al., 2002)	Internalising symptoms (Black et al., 2002) Externalising symptoms (Black et al., 2002)	Moderator (Black et al., 2002)
Paternal depression status (Brennan et al., 2003; Conrad and Hammen, 1993; Gere et al., 2013; Malmberg and Flouri, 2011)	2	4	33 %	Toddlerhood (Malmberg and Flouri, 2011) Childhood (Gere et al., 2013) Adolescence (Brennan et al., 2003; Conrad and Hammen, 1993)	Internalising and externalising symptoms (Malmberg and Flouri, 2011) Depressive symptoms (Gere et al., 2013) Mental health resilience (Brennan et al., 2003) Psychiatric diagnosis (Conrad and Hammen, 1993)	Moderator (Brennan et al., 2003; Conrad and Hammen, 1993; Gere et al., 2013; Malmberg and Flouri, 2011)
Prenatal depression (Braithwaite et al., 2020)	1	1	50 %	Childhood (Braithwaite et al., 2020)	Emotional symptoms (Braithwaite et al., 2020)	Moderator (Braithwaite et al., 2020)
Family functioning (Havinga et al., 2017; Lewandowski et al., 2014)	1	2	33 %	Adulthood (Havinga et al., 2017; Lewandowski et al., 2014)	Onset of mood and anxiety disorders ( Havinga et al., 2017) Mental health resilience (Lewandowski et al., 2014)	Main effect (Havinga et al., 2017; Lewandowski et al., 2014)
Interparental relationship quality (Giallo et al., 2018; Lewandowski et al., 2014; Taraban et al., 2020)	1	4	20 %	Childhood (Giallo et al., 2018; Taraban et al., 2020) Adulthood (Lewandowski et al., 2014)	Internalising symptoms (Taraban et al., 2020) Emotional-behavioural functioning (resilience) (Giallo et al., 2018) Mental health resilience (Lewandowski et al., 2014)	Main effect (Giallo et al., 2018; Lewandowski et al., 2014) Moderator (Taraban et al., 2020)
No relationship changes (Giallo et al., 2018)	0	1	0 %	Childhood (Giallo et al., 2018)	Emotional-behavioural functioning (resilience) (Giallo et al., 2018)	Main effect (Giallo et al., 2018)
Partner or family support to mother (Boyd and Waanders, 2013; Giallo et al., 2018; Lee et al., 2006)	2	3	40 %	Toddlerhood (Lee et al., 2006) Childhood (Giallo et al., 2018) Adolescence (Boyd and Waanders, 2013)	Externalising symptoms (Lee et al., 2006) Internalising symptoms (Lee et al., 2006) Emotional-behavioural functioning (resilience) (Giallo et al., 2018) Depressive symptoms (Boyd and Waanders, 2013)	Moderator (Lee et al., 2006) Main effect (Boyd and Waanders, 2013; Giallo et al., 2018)
Maternal social competence (Conrad and Hammen, 1993)	0	1	0 %	Adolescence (Conrad and Hammen, 1993)	Psychiatric diagnosis (Conrad and Hammen, 1993)	Moderator (Conrad and Hammen, 1993)
Time to self (mother) at least once per week (Giallo et al., 2018)	0	2	0 %	Childhood (Giallo et al., 2018)	Emotional-behavioural functioning (resilience) (Giallo et al., 2018)	Main effect (Giallo et al., 2018)
Maternal involvement in home activities (Giallo et al., 2018)	1	0	100 %	Childhood (Giallo et al., 2018)	Emotional-behavioural functioning (resilience) (Giallo et al., 2018)	Main effect (Giallo et al., 2018)
Child’s positive perception of a mother (Andreas et al., 2017; Conrad and Hammen, 1993)	1	2	33 %	Childhood (Andreas et al., 2017) Adolescence (Conrad and Hammen, 1993)	Psychiatric diagnosis (Conrad and Hammen, 1993) Depressive symptoms (Andreas et al., 2017)	Moderator (Andreas et al., 2017; Conrad and Hammen, 1993)
**Parenting factors**						
Parenting skills (Boyd and Waanders, 2013)	0	1	0 %	Adolescence (Boyd and Waanders, 2013)	Depressive symptoms (Boyd and Waanders, 2013)	Main effect (Boyd and Waanders, 2013)
Parental monitoring (Chen, 2013)	1	1	50 %	Adolescence (Chen, 2013)	Emotional adjustment (Chen, 2013) Behavioural adjustment (Chen, 2013)	Main effect (Chen, 2013)
Parental sensitivity (Vakrat et al., 2018)	1	0	100 %	Childhood (Vakrat et al., 2018)	Psychiatric diagnosis (Vakrat et al., 2018)	Moderator (Vakrat et al., 2018)
Attachment quality (Carlone and Milan, 2021; Fox and Borelli, 2015; Graham and Easterbrooks, 2000; Milan et al., 2009; Woodhouse et al., 2010)	5	3	63 %	Childhood (Carlone and Milan, 2021; Fox and Borelli, 2015; Graham and Easterbrooks, 2000) Adolescence (Milan et al., 2009; Woodhouse et al., 2010)	Depressive symptoms (Fox and Borelli, 2015; Graham and Easterbrooks, 2000; Milan et al., 2009; Woodhouse et al., 2010) Externalising symptoms (Carlone and Milan, 2021)	Moderator (Carlone and Milan, 2021; Graham and Easterbrooks, 2000; Milan et al., 2009; Woodhouse et al., 2010) Main effect (Fox and Borelli, 2015)
Parent-child relationship (Malmberg and Flouri, 2011; Manczak et al., 2018)	4	0	100 %	Toddlerhood (Malmberg and Flouri, 2011) Adolescence (Manczak et al., 2018)	Emotional problems (Malmberg and Flouri, 2011) Externalising problems (Manczak et al., 2018) Internalising problems (Manczak et al., 2018)	Moderator (Malmberg and Flouri, 2011; Manczak et al., 2018)
Parent-child physiological synchrony (West et al., 2020)	1	0	100 %	Childhood (West et al., 2020)	Internalising and externalising symptoms (West et al., 2020)	Moderator (West et al., 2020)
Parental acceptance (Brennan et al., 2003; Owens and Shaw, 2003; Pargas et al., 2010)	1	5	17 %	Childhood (Owens and Shaw, 2003) Adolescence (Brennan et al., 2003) Young adulthood (Pargas et al., 2010)	Externalising problems (Owens and Shaw, 2003) Mental health resilience (Brennan et al., 2003; Pargas et al., 2010)	Moderator (Brennan et al., 2003; Owens and Shaw, 2003; Pargas et al., 2010)
Parental or sibling warmth (Brennan et al., 2003; Chen, 2013; Collishaw et al., 2016; Lewandowski et al., 2014; Pargas et al., 2010)	2	10	17 %	Adolescence (Brennan et al., 2003; Chen, 2013; Collishaw et al., 2016) Young adulthood (Pargas et al., 2010) Adulthood (Lewandowski et al., 2014)	Mental health resilience (Brennan et al., 2003; Collishaw et al., 2016; Lewandowski et al., 2014; Pargas et al., 2010) Mood resilience (Collishaw et al., 2016) Behavioural resilience (Collishaw et al., 2016) Emotional adjustment (Chen, 2013) Behavioural adjustment (Chen, 2013)	Moderator (Brennan et al., 2003; Pargas et al., 2010) Main effect (Chen, 2013; Collishaw et al., 2016; Lewandowski et al., 2014)
Expressed positive emotion (Collishaw et al., 2016; Goodlett et al., 2017; West et al., 2020)	5	1	83 %	Toddlerhood (Goodlett et al., 2017) Childhood (Goodlett et al., 2017; West et al., 2020) Adolescence (Collishaw et al., 2016)	Internalising symptoms (Goodlett et al., 2017) Mental health resilience (Collishaw et al., 2016) Mood resilience (Collishaw et al., 2016) Behavioural resilience (Collishaw et al., 2016) Emotional and behavioural problems (West et al., 2020)	Moderator (Goodlett et al., 2017; West et al., 2020) Main effect (Collishaw et al., 2016)
Co-parent support (Collishaw et al., 2016; Mahedy et al., 2018)	5	1	83 %	Adolescence (Collishaw et al., 2016; Mahedy et al., 2018)	Mental health resilience (Collishaw et al., 2016) Mood resilience (Collishaw et al., 2016; Mahedy et al., 2018) Behavioural resilience (Collishaw et al., 2016; Mahedy et al., 2018) DSM disorder (Mahedy et al., 2018)	Main effect (Collishaw et al., 2016; Mahedy et al., 2018)
Parental criticism (Brennan et al., 2003)	0	2	0 %	Adolescence (Brennan et al., 2003)	Mental health resilience (Brennan et al., 2003)	Moderator (Brennan et al., 2003)
Parental involvement (Chang et al., 2007; Harold et al., 2014; West et al., 2020)	4	3	57 %	Childhood (Chang et al., 2007; West et al., 2020) Adolescence (Harold et al., 2014)	Internalising problems (Chang et al., 2007) Externalising problems (Chang et al., 2007) Depressive symptoms (Harold et al., 2014) Antisocial behaviour (Harold et al., 2014) Emotional and behavioural problems (West et al., 2020)	Moderator (Chang et al., 2007; West et al., 2020) Main effect (Harold et al., 2014)
Parental overinvolvement (Brennan et al., 2003; Lewandowski et al., 2014)	2	2	50 %	Adolescence (Brennan et al., 2003) Adulthood (Lewandowski et al., 2014)	Mental health resilience (Brennan et al., 2003; Lewandowski et al., 2014)	Moderator (Brennan et al., 2003) Main effect (Lewandowski et al., 2014)
Parental firm control (Brennan et al., 2003; Pargas et al., 2010)	0	4	0 %	Adolescence (Brennan et al., 2003) Young adulthood (Pargas et al., 2010)	Mental health resilience (Brennan et al., 2003; Pargas et al., 2010)	Moderator (Brennan et al., 2003; Pargas et al., 2010)
Parental psychological control (Brennan et al., 2003; Pargas et al., 2010)	3	1	75 %	Adolescence (Brennan et al., 2003) Young adulthood (Pargas et al., 2010)	Mental health resilience (Brennan et al., 2003; Pargas et al., 2010)	Moderator (Brennan et al., 2003; Pargas et al., 2010)
Parentification (Hooper et al., 2012)	0	1	0 %	Adolescence (Hooper et al., 2012)	Depressive symptoms (Hooper et al., 2012)	Moderator (Hooper et al., 2012)
**Childcare aspects**						
Childcare quality (Charrois et al., 2017; Goelman et al., 2014)	4	5	44 %	Childhood (Charrois et al., 2017; Goelman et al., 2014)	Externalising problems (Charrois et al., 2017; Goelman et al., 2014) Internalising problems (Charrois et al., 2017; Goelman et al., 2014)	Moderator (Charrois et al., 2017; Goelman et al., 2014)
					Hyperactivity/inattention/distractable behaviour (Charrois et al., 2017; Goelman et al., 2014)	
					Opposition/hostile-aggressive behaviour (Charrois et al., 2017; Goelman et al., 2014)	
					Anxious-fearful behaviour (Goelman et al., 2014)	
Childcare by partner or relative (Smith et al., 2013; Giallo et al., 2018; Herba et al., 2013; Lee et al., 2006)	2	7	22 %	Toddlerhood (Smith et al., 2013; Lee et al., 2006) Childhood (Giallo et al., 2018; Herba et al., 2013)	Externalising problems (Smith et al., 2013; Lee et al., 2006) Internalising problems (Smith et al., 2013; Herba et al., 2013; Lee et al., 2006) Emotional-behavioural functioning (resilience) (Giallo et al., 2018)	Moderator (Smith et al., 2013; Herba et al., 2013; Lee et al., 2006) Main effect (Giallo et al., 2018)
Early or late childcare entry (Herba et al., 2013)	4	5	44 %	Childhood (Herba et al., 2013)	Internalising symptoms (emotional) (Herba et al., 2013) Internalising symptoms (separation anxiety) (Herba et al., 2013) Internalising symptoms (social withdrawal) (Herba et al., 2013)	Moderator (Herba et al., 2013)
Group-based childcare (Herba et al., 2013)	3	3	50 %	Childhood (Herba et al., 2013)	Internalising symptoms (emotional) (Herba et al., 2013) Internalising symptoms (separation anxiety) (Herba et al., 2013) Internalising symptoms (social withdrawal) (Herba et al., 2013)	Moderator (Herba et al., 2013)
Increasing childcare intensity (Herba et al., 2013)	0	3	0 %	Childhood (Herba et al., 2013)	Internalising symptoms (emotional) (Herba et al., 2013)Internalising symptoms (separation anxiety) (Herba et al., 2013) Internalising symptoms (social withdrawal) (Herba et al., 2013)	Moderator (Herba et al., 2013)
Full-time childcare (Herba et al., 2013)	0	6	0 %	Childhood (Herba et al., 2013)	Internalising symptoms (emotional) (Herba et al., 2013) Internalising symptoms (separation anxiety) (Herba et al., 2013) Internalising symptoms (social withdrawal) (Herba et al., 2013)	Moderator (Herba et al., 2013)
**Individual factors**						
Self-esteem (Abela et al., 2012; Abela and Skitch, 2007; Chang and Fu, 2020; Conrad and Hammen, 1993; Lewandowski et al., 2014; Pargas et al., 2010)	3	8	27 %	Childhood (Abela et al., 2012; Abela and Skitch, 2007) Adolescence (Conrad and Hammen, 1993) Young adulthood (Chang and Fu, 2020; Pargas et al., 2010) Adulthood (Lewandowski et al., 2014)	Depressive symptoms (Abela et al., 2012; Abela and Skitch, 2007; Chang and Fu, 2020) Psychiatric diagnosis (Conrad and Hammen, 1993) Mental health resilience (Lewandowski et al., 2014; Pargas et al., 2010)	Main effect (Abela et al., 2012; Abela and Skitch, 2007; Lewandowski et al., 2014) Moderator (Chang and Fu, 2020; Conrad and Hammen, 1993; Pargas et al., 2010)
Self-efficacy (Collishaw et al., 2016)	3	0	100 %	Adolescence (Collishaw et al., 2016)	Mental health resilience (Collishaw et al., 2016) Mood resilience (Collishaw et al., 2016) Behavioural resilience (Collishaw et al., 2016)	Main effect (Collishaw et al., 2016)
Self-control (Chen, 2013)	1	1	50 %	Adolescence (Chen, 2013)	Emotional adjustment (Chen, 2013) Behavioural adjustment (Chen, 2013)	Main effect (Chen, 2013)
Positive rumination (Gilbert et al., 2017)	1	0	100 %	Childhood (Gilbert et al., 2017)	Depressive symptoms (Gilbert et al., 2017)	Moderator (Gilbert et al., 2017)
Optimism (Chen, 2013)	1	1	50 %	Adolescence (Chen, 2013)	Emotional adjustment (Chen, 2013) Behavioural adjustment (Chen, 2013)	Main effect (Chen, 2013)
Resilience (Sun et al., 2015)	0	2	0 %	Adolescence (Sun et al., 2015)	Internalising problems (Sun et al., 2015) Externalising problems (Sun et al., 2015)	Moderator (Sun et al., 2015)
Resourcefulness (Chang et al., 2007)	0	1	0 %	Adolescence (Chang et al., 2007)	Depressive symptoms (Chang et al., 2007)	Moderator (Chang et al., 2007)
Negative emotionality ( Owens and Shaw, 2003)	1	1	50 %	Childhood (Owens and Shaw, 2003)	Externalising problems (Owens and Shaw, 2003)	Moderator (Owens and Shaw, 2003)
Easy temperament ( Lewandowski et al., 2014)	1	1	50 %	Adulthood (Lewandowski et al., 2014)	Mental health resilience (absence of psychiatric diagnosis) (Lewandowski et al., 2014)	Main effect (Lewandowski et al., 2014)
					Mental health resilience (consistent high functioning) (Lewandowski et al., 2014)	
Biological markers of temperament (Davis et al., 2016; Shannon et al., 2007)	2	7	22 %	Childhood (Davis et al., 2016; Shannon et al., 2007)	Conduct problems (Shannon et al., 2007) Depression (Shannon et al., 2007) Child psychopathology symptoms (Davis et al., 2016)	Moderator (Davis et al., 2016; Shannon et al., 2007)
Coping with stress (Monti and Rudolph, 2017; Vreeland et al., 2019)	5	3	63 %	Adolescence (Monti and Rudolph, 2017; Vreeland et al., 2019)	Internalising problems (Vreeland et al., 2019) Externalising problems (Vreeland et al., 2019) Depression (Monti and Rudolph, 2017)	Moderator (Monti and Rudolph, 2017; Vreeland et al., 2019)
Physiological reactivity ( Cummings et al., 2007)	6	10	38 %	Adolescence ( Cummings et al., 2007)	Internalising problems (Cummings et al., 2007) Externalising problems (Cummings et al., 2007)	Moderator (Cummings et al., 2007)
Effortful control (Yan, 2016)	0	6	0 %	Childhood (Yan, 2016)	Internalising problems (Yan, 2016) Externalising problems (Yan, 2016)	Moderator (Yan, 2016)
Inhibitory control (Davidovich et al., 2016; Taraban et al., 2020)	1	1	50 %	Childhood (Taraban et al., 2020) Adolescence (Davidovich et al., 2016)	Internalising problems (Taraban et al., 2020) Depressive symptoms (Davidovich et al., 2016)	Moderator (Taraban et al., 2020) Main effect (Davidovich et al., 2016)
Mental flexibility (Davidovich et al., 2016)	0	1	0 %	Adolescence ( Davidovich et al., 2016)	Depressive symptoms (Davidovich et al., 2016)	Main effect (Davidovich et al., 2016)
Set-shifting (shifting cost) (Davidovich et al., 2016)	0	1	0 %	Adolescence ( Davidovich et al., 2016)	Depressive symptoms (Davidovich et al., 2016)	Main effect (Davidovich et al., 2016)
Reward response (Kujawa et al., 2019; Silk et al., 2006)	4	2	67 %	Childhood (Silk et al., 2006) Adolescence (Kujawa et al., 2019)	Internalising problems (Silk et al., 2006) Depressive symptoms (Kujawa et al., 2019)	Moderator (Kujawa et al., 2019; Silk et al., 2006)
IQ (Havinga et al., 2017; Lewandowski et al., 2014; Pargas et al., 2010)	2	2	50 %	Young adulthood (Pargas et al., 2010) Adulthood (Havinga et al., 2017; Lewandowski et al., 2014)	Mental health resilience (Lewandowski et al., 2014; Pargas et al., 2010) Mood and anxiety disorder (Havinga et al., 2017)	Moderator (Pargas et al., 2010) Main effect (Havinga et al., 2017; Lewandowski et al., 2014)
**Social factors**						
Social skills (Barton et al., 2013; Boyd et al., 2008; Boyd and Waanders, 2013; Conrad and Hammen, 1993)	3	3	50 %	Adolescence (Boyd et al., 2008; Boyd and Waanders, 2013; Conrad and Hammen, 1993) Adulthood (Barton et al., 2013)	Anxiety symptoms (Boyd et al., 2008) Depressive symptoms (Boyd and Waanders, 2013) Psychiatric diagnosis (Conrad and Hammen, 1993) MDD (Barton et al., 2013)	Main effect (Barton et al., 2013; Boyd et al., 2008; Boyd and Waanders, 2013) Moderator (Conrad and Hammen, 1993)
Peer relationships (Collishaw et al., 2016; Conrad and Hammen, 1993; Pargas et al., 2010)	7	4	64 %	Adolescence (Collishaw et al., 2016; Conrad and Hammen, 1993) Young adulthood (Pargas et al., 2010)	Mental health resilience (Collishaw et al., 2016; Pargas et al., 2010) Mood resilience (Collishaw et al., 2016) Behavioural resilience (Collishaw et al., 2016) Psychiatric diagnosis (Conrad and Hammen, 1993)	Main effect (Collishaw et al., 2016) Moderator (Conrad and Hammen, 1993; Pargas et al., 2010)
Prosocial friends (Chen, 2013)	1	1	50 %	Adolescence (Chen, 2013)	Emotional adjustment (Chen, 2013) Behavioural adjustment (Chen, 2013)	Main effect (Chen, 2013)
Non-parent adult support (Casey-Cannon et al., 2006; Conrad and Hammen, 1993)	0	3	0 %	Adolescence (Casey-Cannon et al., 2006; Conrad and Hammen, 1993)	Psychiatric diagnosis (Conrad and Hammen, 1993) Depressive symptoms (Casey-Cannon et al., 2006)	Moderator (Casey-Cannon et al., 2006; Conrad and Hammen, 1993)
Social capital (Delany-Brumsey et al., 2014)	2	2	50 %	Childhood (Delany-Brumsey et al., 2014) Adolescence (Delany-Brumsey et al., 2014)	Internalising problems (Delany-Brumsey et al., 2014) Externalising problems (Delany-Brumsey et al., 2014)	Moderator (Delany-Brumsey et al., 2014)
**Lifestyle factors**						
Religiosity (Jacobs et al., 2012; Kasen et al., 2012; Miller et al., 2012)	2	5	29 %	Adolescence (Jacobs et al., 2012) Adulthood (Kasen et al., 2012; Miller et al., 2012)	Anxiety and depression (Jacobs et al., 2012) MDD (Kasen et al., 2012; Miller et al., 2012) Mood disorder (Kasen et al., 2012) Any psychiatric disorder (Kasen et al., 2012)	Main effect (Jacobs et al., 2012; Kasen et al., 2012; Miller et al., 2012)
Attendance at religious services (Barton et al., 2013; Jacobs et al., 2012; Kasen et al., 2012; Miller et al., 2012)	0	6	0 %	Adolescence (Jacobs et al., 2012) Adulthood (Barton et al., 2013; Kasen et al., 2012; Miller et al., 2012)	Anxiety or depression (Jacobs et al., 2012) MDD (Barton et al., 2013; Kasen et al., 2012; Miller et al., 2012) Mood disorder (Kasen et al., 2012) Any psychiatric disorder (Kasen et al., 2012)	Main effect (Barton et al., 2013; Jacobs et al., 2012; Kasen et al., 2012; Miller et al., 2012)
Exercise (Collishaw et al., 2016)	2	1	67 %	Adolescence (Collishaw et al., 2016)	Mental health resilience (Collishaw et al., 2016) Mood resilience (Collishaw et al., 2016) Behavioural resilience (Collishaw et al., 2016)	Main effect (Collishaw et al., 2016)
Out-of-school activities (Bohnert and Garber, 2007; Collishaw et al., 2016)	1	7	13 %	Adolescence (Bohnert and Garber, 2007; Collishaw et al., 2016)	Mental health resilience (Collishaw et al., 2016) Mood resilience (Collishaw et al., 2016) Behavioural resilience (Collishaw et al., 2016) Externalising symptoms (Bohnert and Garber, 2007) Internalising symptoms (Bohnert and Garber, 2007) Mood disorders (Bohnert and Garber, 2007) Anxiety disorders (Bohnert and Garber, 2007) Behavioural disorders (Bohnert and Garber, 2007)	Main effect (Collishaw et al., 2016) Moderator (Bohnert and Garber, 2007)
**School factors**						
Teacher support (Chen, 2013)	0	2	0 %	Adolescence (Chen, 2013)	Emotional adjustment (Chen, 2013) Behavioural adjustment (Chen, 2013)	Main effect (Chen, 2013)
Academic performance (Conrad and Hammen, 1993)	0	1	0 %	Adolescence (Conrad and Hammen, 1993)	Psychiatric diagnosis (Conrad and Hammen, 1993)	Moderator (Conrad and Hammen, 1993)

**Note**. DSM - Diagnostic and Statistical Manual of Mental Disorders; MDD - major depressive disorder.
