# Supplementary material for: Factors associated with mental health resilience in the child, adolescent and adult offspring of depressed parents: A systematic literature review
Source: J Affect Disord Rep. Author manuscript; Available in PMC 2026 Mar 24. (PMC7618932; doi:10.1016/j.jadr.2025.100983)
Supplement: Supplementary Material [file EMS212714-supplement-Supplementary_Material.docx]

**Supplementary file**

| **Table S1.** Studies excluded at the full-text screening stage and reasons for exclusion. | | |
| --- | --- | --- |
| **Reason for exclusion** | **Articles** | **Comment** |
| **Full-text screening stage (main exclusion criteria)** | | |
| **Ineligible study design** | Fraser & Pakenham (2008), Kendler et al. (2020), Lewin et al. (2015), Liu et al. (2020), Lyons-Ruth et al. (1990), Rounding et al. (2016), Svob & Weissman (2019), Valdez et al. (2011), Valdez et al. (2013), Yu et al. (2021) | Non-randomised interventions, interventions with no control group, non-empirical studies, quasi-experiments, genetic epidemiology studies and other study designs not specified in the systematic review protocol |
| **Incorrect outcome** | Ballarotto (2016), Beardslee et al. (1992), Beardslee et al. (1987), Beardslee et al. (2021), Cornish et al. (2005), Feng et al. (2008), Fulco et al. (2020), Granat et al. (2017), Gruhn et al. (2019), Guedeney et al. (2013), Handley et al. (2017), Johnco & Rapee (2018), Kasen et al. (2014), Knoche et al. (2007), Kujawa et al. (2015), Lee et al. (2020), Li et al. (2019), Ma et al. (2021), Martinson et al. (2016), Mechling (2015), Morgan et al. (2014), Weikum et al. (2013), Pesonen et al. (2004), Petterson & Albers (2001), Pratt et al. (2015), Sacchi et al. (2018), Salisbury et al. (2007), Sang et al. (2019), Schechter et al. (2017), Zelazny et al. (2019), Zhang et al. (2018) | Studies report proxy outcomes for common mental health problems (e.g. internalising or externalising behaviour, depression, anxiety, and behavioural disorders) such as adaptive functioning at school, emotion regulation, psychosocial functioning, brain area, cognitive ability, and temperament. Studies reporting other developmental disorders or mental health outcomes such as suicidal ideation, ADHD, substance use or eating disorders were also excluded |
| **Incorrect exposure** | Agnafors et al. (2017), Annunziato et al. (2007), Bouvette-Turcot et al. (2017), Burke et al. (2016), Carroll et al. (2021), Fraser & Pakenham (2009), Gladstone & Beardslee (2000), Holz et al. (2018), Ivanova & Israel (2005), Kahn et al. (2004), Klasen et al. (2015), Kotch et al. (2014), Küçük et al. (2020), Kwok & Gu (2020), Yoon et al. (2018), Luthar & Sexton (2020), Lachman et al. (2021), McDonald et al. (2016), Morris et al. (2008), Olives et al. (2013), Plass-Christl et al. (2018), Platt et al. (2020), Punamäki et al. (2013), Radke-Yarrow & Brown (1993), Rae-Grant et al. (1989), Rieder et al. (2019), Seifer et al. (1992), Steele & McKinney (2020), Sticca et al. (2020), Stoeckel et al. (2015), Tiet et al. (1998), Van Der Zanden et al. (2010), Van Loon et al. (2015), Van Santvoort et al. (2014), Wurster et al. (2020) | Studies report another type of parental risk exposure than parental depression, such as chronic illness, general psychopathology, psychological distress, suicidal ideation, and maltreatment. Studies using composite or adversity scores, such as a combination of maternal depression, psychosocial risk, and child’s life events, were also excluded |
| **No protective factors** | Milgrom et al. (2019), Abraham et al. (2020), Allen et al. (2019), Bao et al. (2019), Bayer et al. (2010), Beardslee & Podorefsky (1988), Black et al. (2002a), Childs et al. (2001), Choi & Becher (2019), Davis et al. (2019), Du & Kim (2020), Ettekal et al. (2019), Forehand & Smith (1986), Foulon et al. (2015), Hetherington et al. (2018), Jackson (2003), Jackson et al. (2013), Jaser et al. (2011), Letourneau et al. (2019), Loechner et al. (2020), Miller et al. (1997), Murray et al. (2011), Osborn (1990), Pizeta et al. (2017), Priel et al. (2019), Priel et al. (2020), Ranøyen et al. (2015), Riley et al. (2009), Roman et al. (2016), Savage-McGlynn et al. (2015), Schiff et al. (2021), Silk et al. (2007), Sitnick et al. (2014), Swetlitz et al. (2021), Szkody & McKinney (2019), Tannenbaum & Forehand (1994), Thompson et al. (2017), Tolliver-Lynn et al. (2021), Yan et al. (2016), Yeh et al. (2016) | Studies that did not report predictors in high-risk cohorts or moderators in the general population. Examples would include studies examining risk factors only, mediation or moderated mediation analyses, or three- and four-way interactions only. Studies examining moderation as group differences in exposed and unexposed offspring or stratified SEM models were also excluded |
| **Conference abstract** | Glover et al. (2013), Enoch et al. (2014), Karam et al. (2012), Lichenstein et al. (2015), Milgrom et al. (2015), Stopsack et al. (2012), Talati (2014), Sardana et al. (2020) | Studies that seemed to be conference abstracts only, and I did not have access to the PDFs |
| **Duplicate** | Kersten-Alvarez et al. (2010) | - |
| **Did not report effect sizes** | NICHD Early Child Care Research Network (1999), Buckingham-Howes et al. (2017), Carro et al. (1993), Flouri et al. (2017), Franck & Buehler (2007) | - |
| **RCTs** | Beardslee et al. (2003), Beardslee et al. (1997), Burger et al. (2020),  DePanfilis & Dubowitz (2005), Kazdin et al. (2018), Kersten-Alvarez et al. (2010), McManus et al. (2020) | - |
| **Note.** RCTs – randomised controlled trials; ADHD - attention deficit hyperactivity disorder; SEM - structural equation modelling; PDF - portable document format. | | |

| **Table S2.** Characteristics of included studies (N = 60). | |
| --- | --- |
| **Characteristics** | **N (%)** |
| **Countries** | |
| USA  UK  Canada  Australia  Israel  Germany  Netherlands  Norway  Taiwan  China | 40 (66.7%)  6 (10.0%)  4 (6.7%)  2 (5.0%)  2 (3.3%)  1 (1.7%)  1 (1.7%)  1 (1.7%)  1 (1.7%)  1 (1.7%) |
| **Sample sizes** | |
| 0-100  101-250  251-500  501-1000  1000-5000  5000-10000  >10000  Median | 9 (15.0%)  26 (43.3%)  8 (13.3%)  4 (6.7%)  10 (16.7%)  2 (3.3%)  1 (1.7%)  187 |
| **Offspring sex** | |
| Females only  Males only  Both  Missing/Not reported  Median % females | 2 (3.3%)  1 (1.7%)  49 (81.7%)  8 (13.3%)  51% |
| **Offspring age at outcome (developmental stage)*** | |
| Infancy (up to age 1)  Toddlerhood (1 to 3 years old)  Childhood (4 to 10 years old)  Adolescence (11 to 17 years old)  Young adulthood (18 to 25 years old)  Adulthood (> 25 years old) | 0 (0.0%)  5 (8.2%)  26 (42.6%)  23 (37.7%)  2 (3.3%)  5 (8.2%) |
| **Study design** | |
| Cross-sectional  Prospective cohort | 25 (41.7%)  35 (58.3%) |
| **Study type** | |
| Population cohort  High-risk cohort | 43 (71.7%)  17 (28.3%) |
| **The time lag between the protective factor and outcome assessment (prospective studies)** | |
| > 1 year  1 to 2 years  2 to 5 years  6 to 10 years  >10 years  Median (years) | 1 (2.9%)  15 (42.9%)  10 (28.6%)  8 (22.9%)  1 (2.9%)  3 |
| **Parental depression** | |
| Maternal only  Paternal only  Maternal and paternal (separately)  Parental (combined/either) | 37 (61.7%)  1 (1.7%)  6 (10%)  16 (26.7%) |
| **Note.** N – number of studies; USA – United States of America; UK – United Kingdom; * - one study examined protective effects at two developmental stages and was counted twice. | |

| **Table S3.** Risk of bias assessment results for cohort studies. | | | | | | | | | | | | | | |
| --- | --- | --- | --- | --- | --- | --- | --- | --- | --- | --- | --- | --- | --- | --- |
| **Study** | **Q1** | **Q2** | **Q3** | **Q4** | **Q5** | **Q6** | **Q7** | **Q8** | **Q9** | **Q10** | **Q11** | **Total** | **% of yes** | **Risk of bias** |
| Abela et al. (2012) | ✓ | ✓ | ✓ | ✓ | ✓ | ✓ | ✓ | ✓ | 🗶 | U | ✓ | 9 | 82 | Low |
| Abela & Skitch (2007) | ✓ | ✓ | ✓ | ✓ | ✓ | ✓ | ✓ | ✓ | 🗶 | U | ✓ | 9 | 82 | Low |
| Andreas et al. (2017) | ✓ | ✓ | ✓ | 🗶 | 🗶 | U | ✓ | ✓ | ✓ | ✓ | 🗶 | 7 | 64 | Moderate |
| Barton et al. (2013) | ✓ | ✓ | ✓ | ✓ | ✓ | ✓ | ✓ | ✓ | ✓ | U | ✓ | 10 | 91 | Low |
| Black et al. (2002b) | ✓ | ✓ | ✓ | ✓ | ✓ | N/A | ✓ | 🗶 | 🗶 | U | ✓ | 7 | 70 | Low |
| Bohnert & Garber (2007) | ✓ | ✓ | ✓ | ✓ | ✓ | 🗶 | ✓ | ✓ | ✓ | U | ✓ | 9 | 82 | Low |
| Braithwaite et al. (2020) | ✓ | ✓ | ✓ | ✓ | ✓ | N/A | ✓ | ✓ | ✓ | ✓ | ✓ | 10 | 100 | Low |
| Carlone & Milan (2021) | ✓ | ✓ | ✓ | ✓ | ✓ | ✓ | ✓ | ✓ | ✓ | ✓ | ✓ | 11 | 100 | Low |
| Casey-Cannon et al. (2006) | ✓ | ✓ | ✓ | ✓ | ✓ | U | 🗶 | ✓ | 🗶 | 🗶 | ✓ | 7 | 64 | Moderate |
| Chang et al. (2007) | ✓ | ✓ | ✓ | ✓ | ✓ | N/A | ✓ | ✓ | U | U | ✓ | 8 | 80 | Low |
| Chang & Fu (2020) | ✓ | ✓ | ✓ | ✓ | ✓ | N/A | ✓ | ✓ | ✓ | ✓ | ✓ | 10 | 100 | Low |
| Charrois et al. (2017) | ✓ | ✓ | ✓ | ✓ | ✓ | N/A | ✓ | ✓ | 🗶 | ✓ | ✓ | 9 | 90 | Low |
| Chen (2013) | ✓ | ✓ | ✓ | ✓ | ✓ | U | ✓ | ✓ | ✓ | U | ✓ | 9 | 82 | Low |
| Collishaw et al. (2016) | ✓ | ✓ | ✓ | ✓ | ✓ | 🗶 | ✓ | ✓ | 🗶 | ✓ | ✓ | 9 | 82 | Low |
| Conrad & Hammen (1993) | ✓ | ✓ | ✓ | 🗶 | 🗶 | U | ✓ | ✓ | 🗶 | 🗶 | ✓ | 6 | 55 | Moderate |
| Cummings et al. (2007) | ✓ | ✓ | ✓ | ✓ | ✓ | ✓ | ✓ | ✓ | 🗶 | 🗶 | ✓ | 9 | 82 | Low |
| Feldman & Masalha (2007) | ✓ | ✓ | ✓ | ✓ | 0 | N/A | ✓ | ✓ | ✓ | 🗶 | ✓ | 8 | 80 | Low |
| Giallo et al. (2018) | ✓ | ✓ | ✓ | 🗶 | 🗶 | N/A | ✓ | ✓ | ✓ | ✓ | ✓ | 8 | 80 | Low |
| Goelman et al. (2014) | ✓ | ✓ | ✓ | ✓ | ✓ | U | ✓ | ✓ | ✓ | ✓ | ✓ | 10 | 91 | Low |
| Goodlett et al. (2017) | ✓ | ✓ | ✓ | ✓ | ✓ | ✓ | ✓ | ✓ | ✓ | ✓ | ✓ | 11 | 100 | Low |
| Harold et al. (2014) | ✓ | 🗶 | ✓ | 🗶 | 🗶 | N/A | ✓ | ✓ | ✓ | ✓ | ✓ | 7 | 70 | Low |
| Havinga et al. (2017) | ✓ | ✓ | ✓ | ✓ | ✓ | N/A | ✓ | ✓ | ✓ | 🗶 | ✓ | 9 | 90 | Low |
| Herba et al. (2013) | ✓ | ✓ | ✓ | ✓ | ✓ | N/A | ✓ | ✓ | ✓ | ✓ | ✓ | 10 | 100 | Low |
| Kasen et al. (2012) | ✓ | ✓ | ✓ | ✓ | ✓ | ✓ | ✓ | ✓ | ✓ | 🗶 | ✓ | 10 | 91 | Low |
| Kujawa et al. (2019) | ✓ | ✓ | ✓ | ✓ | ✓ | ✓ | ✓ | ✓ | 🗶 | 🗶 | ✓ | 9 | 82 | Low |
| Lee et al. (2006) | ✓ | ✓ | ✓ | ✓ | ✓ | N/A | ✓ | ✓ | 🗶 | 🗶 | ✓ | 8 | 80 | Low |
| Lewandowski et al. (2014) | ✓ | ✓ | ✓ | ✓ | ✓ | ✓ | ✓ | ✓ | ✓ | 🗶 | ✓ | 10 | 91 | Low |
| Mahedy et al. (2018) | ✓ | ✓ | U | ✓ | ✓ | U | ✓ | ✓ | ✓ | ✓ | ✓ | 9 | 82 | Low |
| Malmberg & Flouri (2011) | ✓ | ✓ | ✓ | ✓ | ✓ | N/A | ✓ | ✓ | 🗶 | ✓ | ✓ | 9 | 90 | Low |
| Manczak et al. (2018) | ✓ | ✓ | ✓ | ✓ | ✓ | U | ✓ | ✓ | ✓ | 🗶 | ✓ | 9 | 82 | Low |
| Milan et al. (2009) | 🗶 | ✓ | ✓ | ✓ | ✓ | ✓ | ✓ | ✓ | ✓ | ✓ | ✓ | 10 | 91 | Low |
| Miller et al. (2012) | ✓ | ✓ | U | ✓ | ✓ | ✓ | ✓ | ✓ | ✓ | 🗶 | ✓ | 9 | 82 | Low |
| Monti & Rudolph (2017) | ✓ | ✓ | ✓ | ✓ | ✓ | N/A | ✓ | ✓ | ✓ | ✓ | ✓ | 10 | 100 | Low |
| Owens & Shaw (2003) | U | ✓ | ✓ | 🗶 | 🗶 | N/A | ✓ | ✓ | ✓ | ✓ | ✓ | 7 | 70 | Moderate |
| Pargas et al. (2010) | ✓ | ✓ | ✓ | ✓ | ✓ | ✓ | ✓ | ✓ | ✓ | 🗶 | ✓ | 10 | 91 | Low |
| Taraban et al. (2020) | ✓ | ✓ | ✓ | ✓ | ✓ | N/A | ✓ | ✓ | ✓ | 🗶 | ✓ | 9 | 90 | Low |
| Turney (2011) | U | ✓ | ✓ | ✓ | ✓ | N/A | ✓ | ✓ | ✓ | ✓ | ✓ | 9 | 90 | Low |
| Yan (2016) | ✓ | ✓ | ✓ | ✓ | ✓ | N/A | ✓ | ✓ | 🗶 | 🗶 | ✓ | 8 | 80 | Low |
| **Note.** Q1 – Were the two groups similar and recruited from the same population?/ If inclusion/ exclusion criteria were reported (for high-risk cohorts)?; Q2 – Were the exposures measured similarly to assign people to both exposed and unexposed groups?/ If clearly described how exposure (i.e., parental depression) was measured; Q3 – Was the exposure measured in a valid and reliable way?; Q4 – Were confounding factors identified?; Q5 – Were strategies to deal with confounding factors stated?; Q6 – Were the groups/ participants free of the outcome at the start of the study (or at the moment of exposure)?; Q7 – Were the outcomes measured in a valid and reliable way? Q8 – Was the follow up time reported and sufficient to be long enough for outcomes to occur?/ If clearly reported the time gap between exposure and outcome?; Q9 – Was follow up complete, and if not, were the reasons to loss to follow up described and explored?; Q10 – Were strategies to address incomplete follow up utilized?; Q11 – Was appropriate statistical analysis used?; N/A – not applicable; U – unclear; ✓ - yes; 🗶 - no. | | | | | | | | | | | | | | |

| **Table S4.** Risk of bias assessment results for cross-sectional studies. | | | | | | | | | | | |
| --- | --- | --- | --- | --- | --- | --- | --- | --- | --- | --- | --- |
| **Study** | **Q1** | **Q2** | **Q3** | **Q4** | **Q5** | **Q6** | **Q7** | **Q8** | **Total** | **% of yes** | **Risk of bias** |
| Boyd & Waanders (2013) | ✓ | ✓ | ✓ | ✓ | ✓ | ✓ | ✓ | ✓ | 8 | 100 | Low |
| Boyd et al. (2008) | ✓ | ✓ | U | ✓ | ✓ | ✓ | ✓ | ✓ | 7 | 88 | Low |
| Brennan et al. (2003) | 🗶 | ✓ | ✓ | ✓ | ✓ | ✓ | ✓ | ✓ | 7 | 88 | Low |
| Chang et al. (2007) | 🗶 | ✓ | ✓ | ✓ | ✓ | ✓ | ✓ | ✓ | 7 | 88 | Low |
| Davidovich et al. (2016) | ✓ | ✓ | ✓ | ✓ | ✓ | ✓ | ✓ | ✓ | 8 | 100 | Low |
| Davis et al. (2016) | ✓ | ✓ | ✓ | ✓ | ✓ | ✓ | ✓ | ✓ | 8 | 100 | Low |
| Delany-Brumsey et al. (2014) | ✓ | ✓ | ✓ | ✓ | ✓ | ✓ | ✓ | ✓ | 8 | 100 | Low |
| Fox & Borelli (2015) | 🗶 | ✓ | ✓ | ✓ | 🗶 | 🗶 | ✓ | ✓ | 5 | 63 | Moderate |
| Gere et al. (2013) | ✓ | ✓ | ✓ | ✓ | ✓ | ✓ | ✓ | ✓ | 8 | 100 | Low |
| Gilbert et al. (2017) | ✓ | ✓ | ✓ | ✓ | ✓ | ✓ | ✓ | ✓ | 8 | 100 | Low |
| Goodlett et al. (2017) | ✓ | ✓ | ✓ | ✓ | ✓ | ✓ | ✓ | ✓ | 8 | 100 | Low |
| Graham & Easterbrooks (2000) | 🗶 | ✓ | ✓ | ✓ | 🗶 | 🗶 | ✓ | ✓ | 5 | 63 | Moderate |
| Hooper et al. (2012) | ✓ | ✓ | ✓ | ✓ | 🗶 | 🗶 | ✓ | ✓ | 6 | 75 | Low |
| Jacobs et al. (2012) | U | 🗶 | ✓ | ✓ | U | ✓ | ✓ | ✓ | 5 | 63 | Moderate |
| Shannon et al. (2007) | ✓ | ✓ | ✓ | ✓ | 🗶 | 🗶 | ✓ | ✓ | 6 | 75 | Low |
| Silk et al. (2006) | ✓ | ✓ | ✓ | 🗶 | ✓ | ✓ | ✓ | ✓ | 7 | 88 | Low |
| Smith et al. (2013) | ✓ | ✓ | ✓ | ✓ | ✓ | ✓ | ✓ | ✓ | 8 | 100 | Low |
| Sun et al. (2015) | 🗶 | ✓ | ✓ | ✓ | ✓ | ✓ | ✓ | ✓ | 7 | 88 | Low |
| Vakrat et al. (2018) | ✓ | ✓ | ✓ | ✓ | 🗶 | 🗶 | ✓ | ✓ | 6 | 75 | Low |
| Vreeland et al. (2019) | ✓ | ✓ | ✓ | ✓ | ✓ | ✓ | ✓ | ✓ | 8 | 100 | Low |
| West et al. (2020) | ✓ | ✓ | ✓ | ✓ | ✓ | ✓ | ✓ | ✓ | 8 | 100 | Low |
| Woodhouse et al. (2010) | 🗶 | ✓ | ✓ | ✓ | ✓ | ✓ | ✓ | ✓ | 7 | 88 | Low |
| **Note.** Q1 – Were the criteria for inclusion in the sample clearly defined?; Q2 – Were the study subjects and the setting described in detail?; Q3 – Was the exposure measured in a valid and reliable way?; Q4 – Were objective, standard criteria used for measurement of the condition?; Q5 – Were confounding factors identified?; Q6 – Were strategies to deal with confounding factors stated?; Q7 – Were the outcomes measured in a valid and reliable way?; Q8 – Was appropriate statistical analysis used?; U – unclear; ✓ - yes; 🗶 - no. | | | | | | | | | | | |

| **Table S5.** Strength of evidence by mental health outcome. | | | | | | | | | |
| --- | --- | --- | --- | --- | --- | --- | --- | --- | --- |
| **Protective factor** | **Mental health outcomes** | | | | | | | | |
|  | **Emotional** | | | **Behavioural** | | | **General psychopathology/ resilience** | | |
|  | **Number of models that found evidence** | **Number of models that did not find evidence** | **% of models that found evidence** | **Number of models that found evidence** | **Number of models that did not find evidence** | **% of models that found evidence** | **Number of models that found evidence** | **Number of models that did not find evidence** | **% of models that found evidence** |
| **Demographic characteristics** | | | | | | | | | |
| Household income | 1 | 0 | 100% ^71^ | - | - | - | 1 | 0 | 100% ^66^ |
| More than one child in the household | - | - | - | - | - | - | 0 | 1 | 0% ^66^ |
| More than one adult in the household | - | - | - | - | - | - | 0 | 1 | 0% ^66^ |
| Maternal age | - | - | - | - | - | - | 1 | 0 | 100% ^66^ |
| Maternal tertiary education | - | - | - | - | - | - | 1 | 0 | 100% ^66^ |
| Maternal employment | - | - | - | - | - | - | 0 | 1 | 0% ^66^ |
| Sex | 2 | 1 | 67% ^77, 105, 180^ | 1 | 1 | 50% ^105, 180^ | 0 | 1 | 0% ^66^ |
| Ethnicity | - | - | - | - | - | - | 1 | 0 | 100% ^55^ |
| **Family factors** | | | | | | | | | |
| Grandmother living in the household | 0 | 1 | 0% ^21^ | 0 | 1 | 0% ^21^ | - | - | - |
| Paternal depression status | 2 | 1 | 67% ^65^ | - | - | - | 0 | 3 | 0% ^27, 43, 117^ |
| Prenatal depression | 1 | 1 | 50% ^26^ | - | - | - | - | - | - |
| Family functioning | 1 | 0 | 100% ^77^ | - | - | - | 0 | 2 | 0% ^107^ |
| Interparental relationship quality | 1 | 0 | 100% ^176^ | - | - | - | 0 | 4 | 0% ^66, 107^ |
| No relationship changes | - | - | - | - | - | - | 0 | 1 | 0% ^66^ |
| Partner or family support to mother | 0 | 3 | 0% ^24, 105^ | 1 | 0 | 100% ^105^ | 1 | 0 | 100% ^66^ |
| Maternal social competence | - | - | - | - | - | - | 0 | 1 | 0% ^43^ |
| Time to self (mother) at least once per week | - | - | - | - | - | - | 0 | 2 | 0% ^66^ |
| Maternal involvement in home activities | - | - | - | - | - | - | 1 | 0 | 100% ^66^ |
| Child’s positive perception of a mother | 1 | 1 | 50% ^8^ | - | - | - | 0 | 1 | 0% ^43^ |
| **Parenting factors** | | | | | | | | | |
| Parenting skills | 0 | 1 | 0% ^24^ | - | - | - | - | - | - |
| Parental monitoring | 0 | 1 | 0% ^39^ | 1 | 0 | 100% ^39^ | - | - | - |
| Parental sensitivity | - | - | - | - | - | - | 1 | 0 | 100% ^181^ |
| Attachment quality | 2 | 3 | 40% ^71, 60, 124, 189^ | 3 | 0 | 100% ^31^ | - | - | - |
| Parent-child relationship | 3 | 0 | 100% ^117, 118^ | 1 | 0 | 100% ^118^ | - | - | - |
| Parent-child physiological synchrony | - | - | - | - | - | - | 1 | 0 | 100% ^188^ |
| Parental acceptance | - | - | - | 1 | 1 | 50% ^135^ | 0 | 4 | 0% ^27, 136^ |
| Parental or sibling warmth | 0 | 3 | 0% ^39, 42^ | 1 | 2 | 33% ^39, 42^ | 1 | 5 | 20% ^27, 42, 136, 107^ |
| Expressed positive emotion | 2 | 1 | 67% ^42, 70^ | 1 | 0 | 100% ^42^ | 2 | 0 | 100% ^42, 188^ |
| Co-parent support | 2 | 0 | 100% ^42, 116^ | 1 | 1 | 50% ^42, 116^ | 2 | 0 | 100% ^42, 116^ |
| Parental criticism | - | - | - | - | - | - | 0 | 2 | 0% ^27^ |
| Parental involvement | 1 | 2 | 33% ^36, 76^ | 2 | 1 | 67% ^36, 76^ | 1 | 0 | 100% ^188^ |
| Parental overinvolvement | - | - | - | - | - | - | 2 | 2 | 50% ^27, 107^ |
| Parental firm control | - | - | - | - | - | - | 0 | 4 | 0% ^27, 136^ |
| Parental psychological control | - | - | - | - | - | - | 3 | 1 | 75% ^27, 136^ |
| Parentification | 0 | 1 | 0% ^81^ | - | - | - | - | - | - |
| **Childcare aspects** | | | | | | | | | |
| Childcare quality | 0 | 3 | 0% ^38, 69^ | 4 | 2 | 67% ^38, 69^ | - | - | - |
| Childcare by partner or relative | 2 | 3 | 40% ^78, 105, 165^ | 0 | 2 | 0% ^105, 165^ | 0 | 2 | 0% ^66^ |
| Early or late childcare entry | 4 | 5 | 44% ^78^ | - | - | - | - | - | - |
| Group-based childcare | 3 | 3 | 50% ^78^ | - | - | - | - | - | - |
| Increasing childcare intensity | 0 | 3 | 0% ^78^ | - | - | - | - | - | - |
| Full-time childcare | 0 | 6 | 0% ^78^ | - | - | - | - | - | - |
| **Individual factors** | | | | | | | | | |
| Self-esteem | 1 | 6 | 14% ^4, 3, 37^ | - | - | - | 2 | 2 | 50% ^43, 107, 136^ |
| Self-efficacy | 1 | 0 | 100% ^42^ | 1 | 0 | 100% ^42^ | 1 | 0 | 100% ^42^ |
| Self-control | 0 | 1 | 0% ^39^ | 1 | 0 | 100% ^39^ | - | - | - |
| Positive rumination | 1 | 0 | 100% ^67^ | - | - | - | - | - | - |
| Optimism | 1 | 0 | 100% ^39^ | 0 | 1 | 0% ^39^ | - | - | - |
| Resilience | 0 | 1 | 0% ^170^ | 0 | 1 | 0% ^170^ | - | - | - |
| Resourcefulness | 0 | 1 | 0% ^35^ | - | - | - | - | - | - |
| Negative emotionality | - | - | - | 1 | 1 | 50% ^135^ | - | - | - |
| Easy temperament | - | - | - | - | - | - | 1 | 1 | 50% ^107^ |
| Biological markers of temperament | 0 | 3 | 0% ^161^ | 0 | 3 | 0% ^161^ | 2 | 1 | 67% ^49^ |
| Coping with stress | 4 | 2 | 67% ^187, 128^ | 1 | 1 | 50% ^187^ | - | - | - |
| Physiological reactivity | 4 | 4 | 50% ^46^ | 2 | 6 | 25% ^46^ | - | - | - |
| Effortful control | 0 | 3 | 0% ^191^ | 0 | 3 | 0% ^191^ | - | - | - |
| Inhibitory control | 1 | 1 | 50% ^176, 47^ | - | - | - | - | - | - |
| Mental flexibility | 0 | 1 | 0% ^47^ | - | - | - | - | - | - |
| Set-shifting (shifting cost) | 0 | 1 | 0% ^47^ | - | - | - | - | - | - |
| Reward response | 4 | 2 | 67% ^100, 162^ | - | - | - | - | - | - |
| IQ | 0 | 1 | 0% ^77^ | - | - | - | 2 | 1 | 67% ^107, 136^ |
| **Social factors** | | | | | | | | | |
| Social skills | 3 | 2 | 60% ^12, 24, 25^ | - | - | - | 0 | 1 | 0% ^43^ |
| Peer relationships | 3 | 0 | 100% ^42^ | 3 | 0 | 100% ^42^ | 1 | 4 | 20% ^42, 43, 136^ |
| Prosocial friends | 0 | 1 | 0% ^39^ | 1 | 0 | 100% ^39^ | - | - | - |
| Non-parent adult support | 0 | 2 | 0% ^34^ | - | - | - | 0 | 1 | 0% ^43^ |
| Social capital | 1 | 1 | 50% ^50^ | 1 | 1 | 50% ^50^ | - | - | - |
| **Lifestyle factors** | | | | | | | | | |
| Religiosity | 2 | 4 | 20% ^85, 91, 127^ | - | - | - | 0 | 1 | 0% ^91^ |
| Attendance at religious services | 0 | 5 | 0% ^12, 85, 91, 127^ | - | - | - | 0 | 1 | 0% ^91^ |
| Exercise | 1 | 0 | 100% ^42^ | 0 | 1 | 0% ^42^ | 1 | 0 | 100% ^42^ |
| Out-of-school activities | 1 | 3 | 25% ^22, 42^ | 0 | 3 | 0% ^22, 42^ | 0 | 1 | 0% ^42^ |
| **School factors** | | | | | | | | | |
| Teacher support | 0 | 1 | 0% ^39^ | 0 | 1 | 0% ^39^ | - | - | - |
| Academic performance | - | - | - | - | - | - | 0 | 1 | 0% ^43^ |
| **Note.** IQ – intelligence quotient; 3 – Abela et al. (2012); 4 – Abela et al. (2007); 8 – Andreas et al (2017); 12 - Barton et al. (2013); 21 – Black et al (2002b); 22 - Bohnert et al. (2007); 24 - Boyd and Waanders (2013); 25 - Boyd et al. (2008); 26 – Braithwaite et al (2020); 27 - Brennan et al. (2003); 31 – Carlone and Milan (2021); 34 - Casey-Cannon et al. (2006); 35 - Chang et al. (2007a); 36 - Chang et al. (2007b); 37 - Chang et al. (2020); 38 – Charrois et al. (2017); 39 - Chen et al. (2013); 42 - Collishaw et al. (2016); 43 - Conrad et al. (1993); 46 - Cummings et al. (2007); 47 - Davidovich et al. (2016); 49 – Davis et al (2016); 50 - Delany-Brumsey et al. (2014); 55 - Feldman and Masalha (2007); 66 – Giallo et al. (2018); 60 – Fox and Borelli (2015); 65 – Gere et al (2013); 67 – Gilbert et al (2017); 69 – Goelman et al (2014); 70 - Goodlett et al. (2017); 71 – Graham and Easterbrooks (2000); 76 - Harold et al. (2014); 77 - Havinga et al. (2017); 78 – Herba et al (2013); 81 - Hooper et al. (2012); 85 - Jacobs et al. (2012); 91 - Kasen et al. (2012); 100 - Kujawa et al. (2019); 105 - Lee et al. (2006); 107 - Lewandowski et al. (2014); 116 - Mahedy et al. (2018); 117 - Malmberg and Flouri (2011); 118 - Manczak et al. (2018); 124 - Milan et al. (2009); 127 - Miller et al. (2013); 128 - Monti et al. (2017); 135 – Owens and Shaw (2003); 136 - Pargas et al. (2010); 161 – Shannon et al. (2007); 162 – Silk et al. (2006); 165 - Smith et al. (2013); 170 - Sun et al. (2015); 176 – Taraban et al (2020); 180 – Turney (2011); 181 – Vakrat et al. (2018); 187 - Vreeland et al. (2019); 188 – West et al. (2020); 189 - Woodhouse et al. (2010); 191 – Yan (2016). | | | | | | | | | |

| **Table S6.** Strength of evidence by developmental stage. | | | | | | | | | | | | | | | |
| --- | --- | --- | --- | --- | --- | --- | --- | --- | --- | --- | --- | --- | --- | --- | --- |
| **Protective factor** | **Developmental stage** | | | | | | | | | | | | | | |
|  | **Toddlerhood** | | | **Childhood** | | | **Adolescence** | | | **Young adulthood** | | | **Adulthood** | | |
|  | **E** | **NE** | **%** | **E** | **NE** | **%** | **E** | **NE** | **%** | **E** | **NE** | **%** | **E** | **NE** | **%** |
| **Demographic characteristics** | | | | | | | | | | | | | | | |
| Household income | - | - | - | 2 | 0 | 100% ^66, 71^ | - | - | - | - | - | - | - | - | - |
| More than one child in the household | - | - | - | 0 | 1 | 0% ^66^ | - | - | - | - | - | - | - | - | - |
| More than one adult in the household | - | - | - | 0 | 1 | 0% ^66^ | - | - | - | - | - | - | - | - | - |
| Maternal age | - | - | - | 1 | 0 | 100% ^66^ | - | - | - | - | - | - | - | - | - |
| Maternal tertiary education | - | - | - | 1 | 0 | 100% ^66^ | - | - | - | - | - | - | - | - | - |
| Maternal employment | - | - | - | - | - | - | 0 | 1 | 0% ^43^ | - | - | - | - | - | - |
| Sex | 0 | 2 | 0% ^105^ | 2 | 1 | 67% ^66, 180^ | - | - | - | - | - | - | 1 | 0 | 100% ^77^ |
| Ethnicity | 1 | 0 | 100% ^55^ |  |  |  | - | - | - | - | - | - | - | - | - |
| **Family factors** | | | | | | | | | | | | | | | |
| Grandmother living in the household | - | - | - | 0 | 2 | 0% ^21^ | - | - | - | - | - | - | - | - | - |
| Paternal depression status | 0 | 1 | 0% ^117^ | 2 | 1 | 67% ^65^ | 0 | 2 | 0% ^27, 43^ | - | - | - | - | - | - |
| Prenatal depression | - | - | - | 1 | 1 | 50% ^26^ | - | - | - | - | - | - | - | - | - |
| Family functioning | - | - | - | - | - | - | - | - | - | - | - | - | 1 | 2 | 33% ^77, 107^ |
| Interparental relationship quality | - | - | - | 1 | 2 | 33% ^66, 176^ | - | - | - | - | - | - | 0 | 2 | 0% ^107^ |
| No relationship changes | - | - | - | 0 | 1 | 0% ^66^ | - | - | - | - | - | - | - | - | - |
| Partner or family support to mother | 1 | 1 | 50% ^105^ | 1 | 0 | 100% ^66^ | 0 | 2 | 0% ^24^ | - | - | - | - | - | - |
| Maternal social competence | - | - | - | - | - | - | 0 | 1 | 0% ^43^ | - | - | - | - | - | - |
| Time to self (mother) at least once per week | - | - | - | 0 | 2 | 0% ^66^ | - | - | - | - | - | - | - | - | - |
| Maternal involvement in home activities | - | - | - | 1 | 0 | 100% ^66^ | - | - | - | - | - | - | - | - | - |
| Child’s positive perception of a mother | - | - | - | 1 | 1 | 50% ^8^ | 0 | 1 | 0% ^43^ | - | - | - | - | - | - |
| **Parenting factors** | | | | | | | | | | | | | | | |
| Parenting skills | - | - | - | - | - | - | 0 | 1 | 0% ^24^ | - | - | - | - | - | - |
| Parental monitoring | - | - | - | - | - | - | 1 | 1 | 50% ^39^ | - | - | - | - | - | - |
| Parental sensitivity | - | - | - | 1 | 0 | 100% ^181^ | - | - | - | - | - | - | - | - | - |
| Attachment quality | - | - | - | 4 | 1 | 80% ^31, 60, 71^ | 1 | 2 | 33% ^124, 189^ | - | - | - | - | - | - |
| Parent-child relationship | 2 | 0 | 100% ^117^ | - | - | - | 2 | 0 | 100% ^118^ | - | - | - | - | - | - |
| Parent-child physiological synchrony | - | - | - | 1 | 0 | 100% ^188^ | - | - | - | - | - | - | - | - | - |
| Parental acceptance | - | - | - | 1 | 1 | 50% ^135^ | 0 | 2 | 0% ^27^ | 0 | 2 | 0% ^136^ | - | - | - |
| Parental or sibling warmth | - | - | - | - | - | - | 2 | 7 | 22% ^27, 42, 39^ | 0 | 1 | 0% ^136^ | 0 | 2 | 0% ^107^ |
| Expressed positive emotion | 1 | 0 | 100% ^70^ | 2 | 0 | 100% ^70, 188^ | 2 | 1 | 67% ^42^ | - | - | - | - | - | - |
| Co-parent support | - | - | - | - | - | - | 5 | 1 | 83% ^42, 116^ | - | - | - | - | - | - |
| Parental criticism | - | - | - | - | - | - | 0 | 2 | 0% ^27^ | - | - | - | - | - | - |
| Parental involvement | - | - | - | 3 | 0 | 100% ^36, 188^ | 1 | 3 | 25% ^76^ | - | - | - | - | - | - |
| Parental overinvolvement | - | - | - | - | - | - | 1 | 1 | 50% ^27^ | - | - | - | 1 | 1 | 50% ^107^ |
| Parental firm control | - | - | - | - | - | - | 0 | 2 | 0% ^27^ | 0 | 2 | 0% ^136^ | - | - | - |
| Parental psychological control | - | - | - | - | - | - | 2 | 0 | 100% ^27^ | 1 | 1 | 50% ^136^ | - | - | - |
| Parentification | - | - | - | - | - | - | 0 | 1 | 0% ^81^ | - | - | - | - | - | - |
| **Childcare aspects** | | | | | | | | | | | | | | | |
| Childcare quality | - | - | - | 4 | 5 | 44% ^38, 69^ | - | - | - | - | - | - | - | - | - |
| Childcare by partner or relative | 2 | 2 | 50% ^105, 165^ | 0 | 5 | 0% ^66, 78^ | - | - | - | - | - | - | - | - | - |
| Early or late childcare entry | - | - | - | 4 | 5 | 44% ^78^ | - | - | - | - | - | - | - | - | - |
| Group-based childcare | - | - | - | 3 | 3 | 50% ^78^ | - | - | - | - | - | - | - | - | - |
| Increasing childcare intensity | - | - | - | 0 | 3 | 0% ^78^ | - | - | - | - | - | - | - | - | - |
| Full-time childcare | - | - | - | 0 | 6 | 0% ^78^ | - | - | - | - | - | - | - | - | - |
| **Individual factors** | | | | | | | | | | | | | | | |
| Self-esteem | - | - | - | 0 | 3 | 0% ^3, 4^ | 0 | 1 | 0% ^43^ | 1 | 4 | 20% ^37, 136^ | 2 | 0 | 100% ^107^ |
| Self-efficacy | - | - | - | - | - | - | 3 | 0 | 100% ^42^ | - | - | - | - | - | - |
| Self-control | - | - | - | - | - | - | 1 | 1 | 50% ^39^ | - | - | - | - | - | - |
| Positive rumination | - | - | - | 1 | 0 | 100% ^67^ | - | - | - | - | - | - | - | - | - |
| Optimism | - | - | - | - | - | - | 1 | 1 | 50% ^39^ | - | - | - | - | - | - |
| Resilience | - | - | - | - | - | - | 0 | 2 | 0% ^170^ | - | - | - | - | - | - |
| Resourcefulness | - | - | - | - | - | - | 0 | 1 | 0% ^35^ | - | - | - | - | - | - |
| Negative emotionality | - | - | - | 1 | 1 | 50% ^135^ | - | - | - | - | - | - | - | - | - |
| Easy temperament | - | - | - | - | - | - | - | - | - | - | - | - | 1 | 1 | 50% ^107^ |
| Biological markers of temperament | - | - | - | 2 | 7 | 22% ^49, 161^ | - | - | - | - | - | - | - | - | - |
| Coping with stress | - | - | - | - | - | - | 5 | 3 | 63% ^128, 187^ | - | - | - | - | - | - |
| Physiological reactivity | - | - | - | - | - | - | 6 | 10 | 38% ^46^ | - | - | - | - | - | - |
| Effortful control | - | - | - | 0 | 6 | 0% ^191^ | - | - | - | - | - | - | - | - | - |
| Inhibitory control | - | - | - | 1 | 0 | 100% ^176^ | 0 | 1 | 0% ^47^ | - | - | - | - | - | - |
| Mental flexibility | - | - | - | - | - | - | 0 | 1 | 0% ^47^ | - | - | - | - | - | - |
| Set-shifting (shifting cost) | - | - | - | - | - | - | 0 | 1 | 0% ^47^ | - | - | - | - | - | - |
| Reward response | - | - | - | 2 | 0 | 100% ^162^ | 2 | 2 | 50% ^100^ | - | - | - | - | - | - |
| IQ | - | - | - | - | - | - | - | - | - | 1 | 0 | 100% ^136^ | 1 | 2 | 33% ^77, 107^ |
| **Social factors** | | | | | | | | | | | | | | | |
| Social skills | - | - | - | - | - | - | 2 | 3 | 40% ^24, 25, 43^ | - | - | - | 1 | 0 | 100% ^12^ |
| Peer relationships | - | - | - | - | - | - | 7 | 3 | 70% ^42, 43^ | 0 | 1 | 0% ^136^ | - | - | - |
| Prosocial friends | - | - | - | - | - | - | 1 | 1 | 50% ^39^ | - | - | - | - | - | - |
| Non-parent adult support | - | - | - | - | - | - | 0 | 3 | 0% ^34, 43^ | - | - | - | - | - | - |
| Social capital | - | - | - | 0 | 2 | 0% ^50^ | 2 | 0 | 100% ^50^ | - | - | - | - | - | - |
| **Lifestyle factors** | | | | | | | | | | | | | | | |
| Religiosity | - | - | - | - | - | - | 1 | 1 | 50% ^85^ | - | - | - | 1 | 4 | 20% ^91, 127^ |
| Attendance at religious services | - | - | - | - | - | - | 0 | 1 | 0% ^85^ | - | - | - | 0 | 5 | 0% ^12, 91, 127^ |
| Exercise | - | - | - | - | - | - | 2 | 1 | 67% ^42^ | - | - | - | - | - | - |
| Out-of-school activities | - | - | - | - | - | - | 1 | 7 | 13% ^22, 42^ | - | - | - | - | - | - |
| **School factors** | | | | | | | | | | | | | | | |
| Teacher support | - | - | - | - | - | - | 0 | 2 | 0% ^39^ | - | - | - | - | - | - |
| Academic performance | - | - | - | - | - | - | 0 | 1 | 0% ^43^ | - | - | - | - | - | - |
| **Note.** E – Number of models that found evidence; NE - number of models that did not find evidence for association; % - percentage of models that found evidence; IQ – intelligence quotient; 3 – Abela et al. (2012); 4 – Abela et al. (2007); 8 – Andreas et al (2017); 12 - Barton et al. (2013); 21 – Black et al (2002b); 22 - Bohnert et al. (2007); 24 - Boyd and Waanders (2013); 25 - Boyd et al. (2008); 26 – Braithwaite et al (2020); 27 - Brennan et al. (2003); 31 – Carlone and Milan (2021); 34 - Casey-Cannon et al. (2006); 35 - Chang et al. (2007a); 36 - Chang et al. (2007b); 37 - Chang et al. (2020); 38 – Charrois et al. (2017); 39 - Chen et al. (2013); 42 - Collishaw et al. (2016); 43 - Conrad et al. (1993); 46 - Cummings et al. (2007); 47 - Davidovich et al. (2016); 49 – Davis et al (2016); 50 - Delany-Brumsey et al. (2014); 55 - Feldman and Masalha (2007); 66 – Giallo et al. (2018); 60 – Fox and Borelli (2015); 65 – Gere et al (2013); 67 – Gilbert et al (2017); 69 – Goelman et al (2014); 70 - Goodlett et al. (2017); 71 – Graham and Easterbrooks (2000); 76 - Harold et al. (2014); 77 - Havinga et al. (2017); 78 – Herba et al (2013); 81 - Hooper et al. (2012); 85 - Jacobs et al. (2012); 91 - Kasen et al. (2012); 100 - Kujawa et al. (2019); 105 - Lee et al. (2006); 107 - Lewandowski et al. (2014); 116 - Mahedy et al. (2018); 117 - Malmberg and Flouri (2011); 118 - Manczak et al. (2018); 124 - Milan et al. (2009); 127 - Miller et al. (2013); 128 - Monti et al. (2017); 135 – Owens and Shaw (2003); 136 - Pargas et al. (2010); 161 – Shannon et al. (2007); 162 – Silk et al. (2006); 165 - Smith et al. (2013); 170 - Sun et al. (2015); 176 – Taraban et al (2020); 180 – Turney (2011); 181 – Vakrat et al. (2018); 187 - Vreeland et al. (2019); 188 – West et al. (2020); 189 - Woodhouse et al. (2010); 191 – Yan (2016). | | | | | | | | | | | | | | | |

**References**

Abela, J. R. Z., & Skitch, S. A. (2007). Dysfunctional attitudes, self-esteem, and hassles: Cognitive vulnerability to depression in children of affectively ill parents. *Behaviour Research and Therapy*, 45(6), 1127–1140. https://doi.org/10.1016/j.brat.2006.09.011

Abela, J. R. Z., Fishman, M. B., Cohen, J. R., & Young, J. F. (2012). Personality Predispositions to Depression in Children of Affectively-Ill Parents: The Buffering Role of Self-Esteem. *Journal of Clinical Child and Adolescent Psychology*, 41(4), 391–401. https://doi.org/10.1080/15374416.2012.654463

Abraham, E., Posner, J., Wickramaratne, P. J., Aw, N., van Dijk, M. T., Cha, J., Weissman, M. M., & Talati, A. (2020). Concordance in parent and offspring cortico-basal ganglia white matter connectivity varies by parental history of major depressive disorder and early parental care. *Social Cognitive and Affective Neuroscience*, 15(8), 889–903. https://doi.org/10.1093/scan/nsaa118

Agnafors, S., Svedin, C. G., Oreland, L., Bladh, M., Comasco, E., & Sydsjö, G. (2017). A Biopsychosocial Approach to Risk and Resilience on Behavior in Children Followed from Birth to Age 12. *Child Psychiatry and Human Development*, 48(4), 584–596. https://doi.org/10.1007/s10578-016-0684-x

Allen, T. A., Oshri, A., Rogosch, F. A., Toth, S. L., & Cicchetti, D. (2019). Offspring Personality Mediates the Association between Maternal Depression and Childhood Psychopathology. *Journal of Abnormal Child Psychology*, 47(2), 345–357. https://doi.org/10.1007/s10802-018-0453-3

Andreas, A., Otto, Y., Stadelmann, S., Schlesier-Michel, A., von Klitzing, K., & Klein, A. M. (2017). Gender Specificity of Children’s Narrative Representations in Predicting Depressive Symptoms at Early School Age. *Journal of Child and Family Studies*, 26(1), 148–160. https://doi.org/10.1007/s10826-016-0533-3

Annunziato, R. A., Rakotomihamina, V., & Rubacka, J. (2007). Examining the Effects of Maternal Chronic Illness on Child Well-Being in Single Parent Families. *Journal of Developmental & Behavioral Pediatrics*, 28(5), 386-391.

Ballarotto, G. (2016). Parent-Child Interactions in Families with Mothers who had Experienced Early Relational Traumas. *THE EUROPEAN PROCEEDINGS OF SOCIAL & BEHAVIOURAL SCIENCES*, 13, 285-294. https://doi.org/10.15405/epsbs.2016.07.02.28

Bao, J., Gudmunson, C. G., Greder, K., & Smith, S. R. (2019). The Impact of Family Rituals and Maternal Depressive Symptoms on Child Externalizing Behaviors: An Urban–Rural Comparison. *Child and Youth Care Forum*, 48(6), 935–953. https://doi.org/10.1007/s10566-019-09512-w

Barton, Y. A., Miller, L., Wickramaratne, P., Gameroff, M. J., & Weissman, M. M. (2013). Religious attendance and social adjustment as protective against depression: A 10-year prospective study. *Journal of Affective Disorders*, 146(1), 53–57. https://doi.org/10.1016/j.jad.2012.08.037

Bayer, J. K., Hastings, P. D., Sanson, A. V., Ukoumunne, O. C., & Rubin, K. H. (2010). Predicting Mid-Childhood Internalising Symptoms: A Longitudinal Community Study. *International Journal of Mental Health Promotion*, 12(1), 5–17. https://doi.org/10.1080/14623730.2010.9721802

Beardslee, W. R., & Podorefsky, D. (1988). Resilient Adolescents Whose Parents Have Serious Affective and Other Psychiatric Disorders: Importance of Self-Understanding and Relationships. *The American Journal of Psychiatry*, 145(1), 63-69.

Beardslee, W. R., G Gladstone, T. R., Wright, E. J., & Cooper, A. B. (2003). A Family-Based Approach to the Prevention of Depressive Symptoms in Children at Risk: Evidence of Parental and Child Change. *Pediatrics*, 112(2), e119-e131. http://www.pediatrics.org/cgi/content/full/112/2/

Beardslee, W. R., Hoke, L., Wheelock, I., Rothberg, P. C., Van De Velde, P., & Swatling, S. W. (1992). Initial Findings on Preventive Intervention for Families With Parental Affective Disorders. *The American Journal of Psychiatry,* 149(10), 1335-1340*.*

Beardslee, W. R., Schultz, L. H., & Selman, R. L. (1987). Level of Social-Cognitive Development, Adaptive Functioning, and DSM-III Diagnoses in Adolescent Offspring of Parents With Affective Disorders: Implications of the Development of the Capacity for Mutuality. *Developmental Psychology*, 23(6), 807–815.

Beardslee, W. R., Versage, E. M., Wright, E. J., Salt, P., Rothberg, P. C., Drezner, K., & Gladstone, T. R. G. (2021). Examination of preventive interventions for families with depression: Evidence of change. *Development and Psychopathology*, 9(1), 109-130. https://www.cambridge.org/core.

Beardslee, W. R., Wright, E. J., Salt, P., Drezner, K., Gladstone, T. R. G., Versage, E. M., & Rothberg, P. C. (1997). Examination of children’s responses to two preventive intervention strategies over time. *Journal of the American Academy of Child and Adolescent Psychiatry*, 36(2), 196–204. https://doi.org/10.1097/00004583-199702000-00010

Black, M. M., Papas, M. A., Hussey, J. M., Dubowitz, H., Kotch, J. B., & Starr, R. H. (2002a). Behavior Problems Among Preschool Children Born to Adolescent Mothers: Effects of Maternal Depression and Perceptions of Partner Relationships. *Journal of Clinical Child and Adolescent Psychology*, 31(1), 16-26.

Black, M. M., Papas, M. A., Hussey, J. M., Hunter, W., Dubowitz, H., Kotch, J. B., English, D., & Schneider, M. (2002b). Behavior and Development of Preschool Children Born to Adolescent Mothers: Risk and 3-Generation Households. *Pediatrics*, 109(4), 573-580.

Bohnert, A. M., & Garber, J. (2007). Prospective relations between organized activity participation and psychopathology during adolescence. *Journal of Abnormal Child Psychology*, 35(6), 1021–1033. https://doi.org/10.1007/s10802-007-9152-1

Bouvette-Turcot, A. A., Bernier, A., & Leblanc, É. (2017). Maternal Psychosocial Maladjustment and Child Internalizing Symptoms: Investigating the Modulating Role of Maternal Sensitivity. *Journal of Abnormal Child Psychology*, 45(1), 157–170. https://doi.org/10.1007/s10802-016-0154-8

Boyd, R. C., & Waanders, C. (2013). Protective Factors for Depression Among African American Children of Predominantly Low-Income Mothers with Depression. *Journal of Child and Family Studies*, 22(1), 85–95. https://doi.org/10.1007/s10826-012-9588-y

Boyd, R. C., Wooden, T. D., Munro, M. A., Liu, T., & Have, T. Ten. (2008). The Impact of Community Violence Exposure on Anxiety in Children of Mothers with Depression. *Journal of Child & Adolescent Trauma*, 1(4), 287–300. https://doi.org/10.1080/19361520802505669

Braithwaite, E. C., Pickles, A., Wright, N., Sharp, H., & Hill, J. (2020). Sex differences in foetal origins of child emotional symptoms: a test of evolutionary hypotheses in a large, general population cohort. *Journal of Child Psychology and Psychiatry and Allied Disciplines*, 61(11), 1194–1202. https://doi.org/10.1111/jcpp.13229

Brennan, P. A., Le Brocque, R., & Hammen, C. (2003). Maternal depression, parent-child relationships, and resilient outcomes in adolescence. *J Am Acad Child Adolesc Psychiatry*, 42(12), 1469–1477. https://doi.org/10.1097/00004583-200312000-00014

Buckingham-Howes, S., Wang, Y., Oberlander, S. E., & Black, M. M. (2017). Early maternal depressive symptom trajectories: Associations with 7-year maternal depressive symptoms and child behavior. *Journal of Family Psychology*, 31(4), 387–397. https://doi.org/10.1037/fam0000242

Burger, H., Verbeek, T., Aris-Meijer, J. L., Beijers, C., Mol, B. W., Hollon, S. D., Ormel, J., Van Pampus, M. G., & Bockting, C. L. H. (2020). Effects of psychological treatment of mental health problems in pregnant women to protect their offspring: Randomised controlled trial. *British Journal of Psychiatry*, 216(4), 182–188. https://doi.org/10.1192/bjp.2019.260

Burke, T. A., Connolly, S. L., Hamilton, J. L., Stange, J. P., Abramson, L. Y., & Alloy, L. B. (2016). Cognitive Risk and Protective Factors for Suicidal Ideation: A Two Year Longitudinal Study in Adolescence. *Journal of Abnormal Child Psychology*, 44(6), 1145–1160. https://doi.org/10.1007/s10802-015-0104-x

Carlone, C., & Milan, S. (2021). Maternal Depression and Child Externalizing Behaviors: The Role of Attachment Across Development in Low-income Families. *Research on Child and Adolescent Psychopathology*, 49(5), 603–614. https://doi.org/10.1007/s10802-020-00747-z

Carro, M. G., Grant, K. E., Gotlib, I. H., & Compas, B. E. (1993). Postpartum depression and child development: An investigation of mothers and fathers as sources of risk and resilience. *Development and Psychopathology*, 5(4), 567-579.

Carroll, H., Rondon, M. B., Sanchez, S. E., Fricchione, G. L., Williams, M. A., & Gelaye, B. (2021). Resilience mediates the relationship between household dysfunction in childhood and postpartum depression in adolescent mothers in Peru. *Comprehensive Psychiatry*, 104. https://doi.org/10.1016/j.comppsych.2020.152215

Casey-Cannon, S., Pasch, L. A., Tschann, J. M., & Flores, E. (2006). Nonparent adult social support and depressive symptoms among Mexican American and European American adolescents. *Journal of Early Adolescence*, 26(3), 318–343. https://doi.org/10.1177/0272431606288592

Chang, H. J., Zauszniewski, J. A., Heinzer, M. M., Musil, C. M., & Tsai, W. C. (2007). Adaptive functioning and depressive symptoms in school-aged children. *Journal of Advanced Nursing*, 60(5), 502–512. https://doi.org/10.1111/j.1365-2648.2007.04440.x

Chang, J. J., Halpern, C. T., & Kaufman, J. S. (2007). Maternal Depressive Symptoms, Father’s Involvement, and the Trajectories of Child Problem Behaviors in a US National Sample. *Arch Pediatr Adolesc Med*, 161(7).

Chang, L. Y., & Fu, M. (2020). Disentangling the effects of intergenerational transmission of depression from adolescence to adulthood: the protective role of self-esteem. *European Child and Adolescent Psychiatry*, 29(5), 679–689. https://doi.org/10.1007/s00787-019-01390-w

Charrois, J., Côté, S. M., Japel, C., Séguin, J. R., Paquin, S., Tremblay, R. E., & Herba, C. M. (2017). Child-care quality moderates the association between maternal depression and children’s behavioural outcome. *Journal of Child Psychology and Psychiatry and Allied Disciplines*, 58(11), 1210–1218. https://doi.org/10.1111/jcpp.12764

Chen, H. J. (2013). Robust protective factors for African American youths who have a parent with depression. *Social Work Research*, 37(2), 121–134. https://doi.org/10.1093/swr/svs026

Childs, H. F., Schneider, H. G., & Dula, C. S. (2001). Adolescent adjustment: Maternal depression and social competence. *International Journal of Adolescence and Youth*, 9(2–3), 175–184. https://doi.org/10.1080/02673843.2001.9747875

Choi, J. K., & Becher, E. H. (2019). Supportive Coparenting, Parenting Stress, Harsh Parenting, and Child Behavior Problems in Nonmarital Families. *Family Process*, 58(2), 404–417. https://doi.org/10.1111/famp.12373

Collishaw, S., Hammerton, G., Mahedy, L., Sellers, R., Owen, M. J., Craddock, N., Thapar, A. K., Harold, G. T., Rice, F., & Thapar, A. (2016). Mental health resilience in the adolescent offspring of parents with depression: A prospective longitudinal study. *The Lancet Psychiatry*, 3(1), 49–57. https://doi.org/10.1016/S2215-0366(15)00358-2

Conrad, M., & Hammen, C. (1993). Protective and resource factors in high-and low-risk children: A comparison of children with unipolar, bipolar, medically ill, and normal mothers. *Development and Psychopathology*, 5(4), 593-607.

Cornish, A. M., McMahon, C. A., Ungerer, J. A., Barnett, B., Kowalenko, N., & Tennant, C. (2005). Postnatal depression and infant cognitive and motor development in the second postnatal year: The impact of depression chronicity and infant gender. *Infant Behavior and Development*, 28(4), 407–417. https://doi.org/10.1016/j.infbeh.2005.03.004

Cummings, E. M., El-Sheikh, M., Kouros, C. D., & Keller, P. S. (2007). Children’s skin conductance reactivity as a mechanism of risk in the context of parental depressive symptoms. *Journal of Child Psychology and Psychiatry and Allied Disciplines*, 48(5), 436–445. https://doi.org/10.1111/j.1469-7610.2006.01713.x

Davidovich, S., Collishaw, S., Thapar, A. K., Harold, G., Thapar, A., & Rice, F. (2016). Do better executive functions buffer the effect of current parental depression on adolescent depressive symptoms? *Journal of Affective Disorders*, 199, 54–64. https://doi.org/10.1016/j.jad.2016.03.049

Davis, M., Goodman, S. H., Lavner, J. A., Maier, M. S., Stowe, Z. N., Newport, D. J., & Knight, B. (2019). Patterns of positivity: Positive affect trajectories among infants of mothers with a history of depression. *Infancy*, 24(6), 911–932. https://doi.org/10.1111/infa.12314

Davis, M., Suveg, C., Whitehead, M., Jones, A., & Shaffer, A. (2016). Preschoolers’ psychophysiological responses to mood induction tasks moderate the intergenerational transmission of internalizing problems. *Biological Psychology*, 117, 159–169. https://doi.org/10.1016/j.biopsycho.2016.03.015

Delany-Brumsey, A., Mays, V. M., & Cochran, S. D. (2014). Does Neighborhood Social Capital Buffer the Effects of Maternal Depression on Adolescent Behavior Problems? *American Journal of Community Psychology*, 53(3–4), 275–285. https://doi.org/10.1007/s10464-014-9640-8

DePanfilis, D., & Dubowitz, H. (2005). Family connections: A program for preventing child neglect. *Child Maltreatment*, 10(2), 108–123. https://doi.org/10.1177/1077559505275252

Du, X., & Kim, Y. K. (2020). Family functioning and adolescent behavior problems: A moderated mediation model of caregiver depression and neighborhood collective efficacy. *Children and Youth Services Review*, 116. https://doi.org/10.1016/j.childyouth.2020.105270

Enoch, M.-A., Kitzman, H., Smith, J., Anson, E., Hodgkinson, C., Goldman, D., & Olds, D. (2014). A Longitudinal Study in Mothers and Firstborn Children of Genetic and Environmental Influences on Externalizing and Internalizing Disorders across Development. *NEUROPSYCHOPHARMACOLOGY*, 39, S401–S402.

Erin N. Smith, Josefina M. Grau, Petra A. Duran, & Patricia Castellanos. (2013). Maternal Depressive Symptoms and Child Behavior Problems Among Latina Adolescent Mothers: The Buffering Effect of Mother-Reported Partner Child Care Involvement. *Merrill-Palmer Quarterly*, 59(3), 304. https://doi.org/10.13110/merrpalmquar1982.59.3.0304

Ettekal, I., Eiden, R. D., Nickerson, A. B., Molnar, D. S., & Schuetze, P. (2019). Developmental cascades to children’s conduct problems: The role of prenatal substance use, socioeconomic adversity, maternal depression and sensitivity, and children’s conscience. *Development and Psychopathology*, 32(1), 85–103. https://doi.org/10.1017/S095457941800144X

Feldman, R., & Masalha, S. (2007). The role of culture in moderating the links between early ecological risk and young children’s adaptation. *Development and Psychopathology*, 19, 1–21. https://doi.org/10.10170S0954579407070010

Feng, X., Shaw, D. S., Kovacs, M., Lane, T., O’Rourke, F. E., & Alarcon, J. H. (2008). Emotion regulation in preschoolers: The roles of behavioral inhibition, maternal affective behavior, and maternal depression. *Journal of Child Psychology and Psychiatry and Allied Disciplines*, 49(2), 132–141. https://doi.org/10.1111/j.1469-7610.2007.01828.x

Flouri, E., Ruddy, A., & Midouhas, E. (2017). Maternal depression and trajectories of child internalizing and externalizing problems: The roles of child decision making and working memory. *Psychological Medicine*, 47(6), 1138–1148. https://doi.org/10.1017/S0033291716003226

Forehand, R., & Smith, K. A. (1986). Who depresses whom? A look at the relationship of adolescent mood to maternal and paternal mood. *Child Study Journal*.

Foulon, S., Pingault, J. B., Larroque, B., Melchior, M., Falissard, B., & Côté, S. M. (2015). Developmental predictors of inattention-hyperactivity from pregnancy to early childhood. *PLoS ONE*, 10(5). https://doi.org/10.1371/journal.pone.0125996

Fox, M. K., & Borelli, J. L. (2015). Attachment Moderates the Association Between Mother and Child Depressive Symptoms. *Psi Chi Journal of Psychological Research*, 20(1), 29–36. https://doi.org/10.24839/2164-8204.jn20.1.29

Franck, K. L., & Buehler, C. (2007). A Family Process Model of Marital Hostility, Parental Depressive Affect, and Early Adolescent Problem Behavior: The Roles of Triangulation and Parental Warmth. *Journal of Family Psychology*, 21(4), 614–625. https://doi.org/10.1037/0893-3200.21.4.614

Fraser, E., & Pakenham, K. I. (2008). Evaluation of a resilience-based intervention for children of parents with mental illness. *Australian & New Zealand Journal of Psychiatry*, 42(12), 1041-1050.

Fraser, E., & Pakenham, K. I. (2009). Resilience in children of parents with mental illness: Relations between mental health literacy, social connectedness and coping, and both adjustment and caregiving. *Psychology, Health and Medicine*, 14(5), 573–584. https://doi.org/10.1080/13548500903193820

Fulco, C. J., Bears Augustyn, M., & Henry, K. L. (2020). Maternal Depressive Symptoms and Adolescent Health Risk Problems: The Role of School Engagement. *Journal of Youth and Adolescence*, 49(1), 102–118. https://doi.org/10.1007/s10964-019-01046-7

Gere, M. K., Hagen, K. A., Villabø, M. A., Arnberg, K., Neumer, S. P., & Torgersen, S. (2013). Fathers’ mental health as a protective factor in the relationship between maternal and child depressive symptoms. *Depression and Anxiety*, 30(1), 31–38. https://doi.org/10.1002/da.22010

Giallo, R., Gartland, D., Woolhouse, H., Mensah, F., Westrupp, E., Nicholson, J., & Brown, S. (2018). Emotional–behavioral resilience among children of first-time mothers with and without depression across the early childhood period. *International Journal of Behavioral Development*, 42(2), 214–224. https://doi.org/10.1177/0165025416687413

Gilbert, K., Luking, K., Pagliaccio, D., Luby, J., & Barch, D. (2017). Dampening, Positive Rumination, and Positive Life Events: Associations with Depressive Symptoms in Children at Risk for Depression. *Cognitive Therapy and Research*, 41(1), 31–42. https://doi.org/10.1007/s10608-016-9798-5

Gladstone, T. R. G., & Beardslee, W. R. (2000). The prevention of depression in at-risk adolescents: Current and future directions. *Journal of Cognitive Psychotherapy*, 14(1), 9-23.

Glover, J. H., O’Hara, M., Omay, O., Laure, A., Sutter-Dallay, K. W., Cazas, A. C., Dugnat, M., Glatigny, E., Isserlis, C., & Lagarde, F. (2013). The Marcé International Society International Biennial General Scientific Meeting.

Goelman, H., Zdaniuk, B., Boyce, W. T., Armstrong, J. M., & Essex, M. J. (2014). Maternal mental health, child care quality, and children’s behavior. *Journal of Applied Developmental Psychology*, 35(4), 347–356. https://doi.org/10.1016/j.appdev.2014.05.003

Goodlett, B. D., Trentacosta, C. J., McLear, C., Crespo, L., Wheeler, R., Williams, A., Chaudhry, K., & Smith-Darden, J. (2017). Maternal depressive symptoms and at-risk young children’s internalizing problems: The moderating role of mothers’ positivity. *Merrill-Palmer Quarterly*, 63(1), 77–104. https://doi.org/10.13110/merrpalmquar1982.63.1.0077

Graham, C. A., & Easterbrooks, M. A. (2000). School-aged children’s vulnerability to depressive symptomatology: The role of attachment security, maternal depressive symptomatology, and economic risk. *Development and Psychopathology*, 12(2), 201-213.

Granat, A., Gadassi, R., Gilboa-Schechtman, E., & Feldman, R. (2017). Maternal depression and anxiety, social synchrony, and infant regulation of negative and positive emotions. *Emotion*, 17(1), 11–27. https://doi.org/10.1037/emo0000204

Gruhn, M. A., Bettis, A. H., Murphy, L. K., Dunbar, J. P., Reising, M. M., Forehand, R., & Compas, B. E. (2019). Coping and Observed Emotions in Children of Parents with a History of Depression. *Journal of Child and Family Studies*, 28(6), 1581–1594. https://doi.org/10.1007/s10826-019-01390-z

Guedeney, A., Wendland, J., Dugravier, R., Saïas, T., Tubach, F., Welniarz, B., Guedeney, N., Greacen, T., Tereno, S., & Pasquet, B. (2013). Impact of a randomized home-visiting trial on infant social withdrawal in the CAPEDP prevention study. *Infant Mental Health Journal*, 34(6), 594–601. https://doi.org/10.1002/imhj.21413

Handley, E. D., Michl-Petzing, L. C., Rogosch, F. A., Cicchetti, D., & Toth, S. L. (2017). Developmental cascade effects of interpersonal psychotherapy for depressed mothers: Longitudinal associations with toddler attachment, temperament, and maternal parenting efficacy. *Development and Psychopathology*, 29(2), 601–615. https://doi.org/10.1017/S0954579417000219

Harold, G. T., Leve, L. D., Kim, H. K., Mahedy, L., Gaysina, D., Thapar, A., & Collishaw, S. (2014). Maternal caregiving and girls’ depressive symptom and antisocial behavior trajectories: An examination among high-risk youth. *Development and Psychopathology*, 26, 1461–1475. https://doi.org/10.1017/S095457941400114X

Havinga, P. J., Boschloo, L., Bloemen, A. J. P., Nauta, M. H., De Vries, S. O., Penninx, B. W. J. H., Schoevers, R. A., & Hartman, C. A. (2017). Doomed for disorder? High incidence of mood and anxiety disorders in offspring of depressed and anxious patients: A prospective cohort study. *Journal of Clinical Psychiatry*, 78(1), e8–e17. https://doi.org/10.4088/JCP.15m09936

Herba, C. M., Tremblay, R. E., Boivin, M., Liu, X., Mongeau, C., Séguin, J. R., & Coté, S. M. (2013). Maternal depressive symptoms and children’s emotional problems can early child care help children of depressed mothers? *JAMA Psychiatry*, 70(8), 830–838. https://doi.org/10.1001/jamapsychiatry.2013.1361

Hetherington, E., Mcdonald, S., Racine, N., & Tough, S. (2018). Risk and Protective Factors for Externalizing Behavior at 3 Years: Results from the All Our Families Pregnancy Cohort. *Journal of Developmental & Behavioral Pediatrics*, 39(7), 547-554.

Holz, N. E., Boecker-Schlier, R., Jennen-Steinmetz, C., Hohm, E., Buchmann, A. F., Blomeyer, D., Baumeister, S., Plichta, M. M., Esser, G., Schmidt, M., Meyer-Lindenberg, A., Banaschewski, T., Brandeis, D., & Laucht, M. (2018). Early maternal care may counteract familial liability for psychopathology in the reward circuitry. *Social Cognitive and Affective Neuroscience*, 13(11), 1191–1201. https://doi.org/10.1093/scan/nsy087

Hooper, L. M., Tomek, S. E., Doehler, K., & Jankowski, P. J. (2012). Patterns of Self-Reported Alcohol Use, Depressive Symptoms, and Body Mass Index in a Family Sample: The Buffering Effects of Parentification. *The Family Journal*, 20(2), 164–178. https://doi.org/10.1177/1066480711435320

Ivanova, M. Y., & Israel, A. C. (2005). Family Stability as a Protective Factor Against the Influences of Pessimistic Attributional Style on Depression. *Cognitive Therapy and Research*, 29(2), 243–251. https://doi.org/10.1007/s10608-005-3167-0

Jackson, A. P. (2003). The effects of family and neighborhood characteristics on the behavioral and cognitive development of poor black children: A longitudinal study. *American Journal of Community Psychology*, 32(1–2), 175–186. https://doi.org/10.1023/A:1025615427939

Jackson, A. P., Preston, K. S. J., & Thomas, C. A. (2013). Single mothers, nonresident fathers, and preschoolers’ socioemotional development: Social support, psychological well-being, and parenting quality. *Journal of Social Service Research*, 39(1), 129–140. https://doi.org/10.1080/01488376.2012.723241

Jacobs, M., Miller, L., Wickramaratne, P., Gameroff, M., & Weissman, M. M. (2012). Family religion and psychopathology in children of depressed mothers: Ten-year follow-up. *Journal of Affective Disorders*, 136(3), 320–327. https://doi.org/10.1016/j.jad.2011.11.030

Jaser, S. S., Champion, J. E., Dharamsi, K. R., Riesing, M. M., & Compas, B. E. (2011). Coping and Positive Affect in Adolescents of Mothers With and Without a History of Depression. *Journal of Child and Family Studies*, 20(3), 353–360. https://doi.org/10.1007/s10826-010-9399-y

Johnco, C., & Rapee, R. M. (2018). Depression literacy and stigma influence how parents perceive and respond to adolescent depressive symptoms. *Journal of Affective Disorders*, 241, 599-607.

Kahn, R. S., Brandt, D., & Whitaker, R. C. (2004). Combined Effect of Mothers’ and Fathers’ Mental Health Symptoms on Children’s Behavioral and Emotional Well-being. *Arch Pediatr Adolesc Med*, 158, 721–729.

Karam, F., Berard, A., Sheehy, O., Huneau, M.-C., Briggs, G., Chambers, C., Einarson, A., Jonhson, D., Kao, K., & Koren, G. (2012). Impact of Maternal Attachment on Infant Development at 1-Year of Age: Results from the OTIS Antidepressants in Pregnancy Study. *PHARMACOEPIDEMIOLOGY AND DRUG SAFETY*, 21, 151–152.

Kasen, S., Wickramaratne, P., & Gameroff, M. J. (2014). Religiosity and longitudinal change in psychosocial functioning in adult offspring of depressed parents at high risk for major depression. *Depression and Anxiety*, 31(1), 63–71. https://doi.org/10.1002/da.22131

Kasen, S., Wickramaratne, P., Gameroff, M. J., & Weissman, M. M. (2012). Religiosity and resilience in persons at high risk for major depression. *Psychological Medicine*, 42(3), 509–519. https://doi.org/10.1017/S0033291711001516

Kazdin, A. E., Glick, A., Pope, J., Kaptchuk, T. J., Lecza, B., Carrubba, E., McWhinney, E., & Hamilton, N. (2018). Parent management training for conduct problems in children: Enhancing treatment to improve therapeutic change. *International Journal of Clinical and Health Psychology*, 18(2), 91–101. https://doi.org/10.1016/j.ijchp.2017.12.002

Kendler, K. S., Ohlsson, H., Sundquist, J., & Sundquist, K. (2020). The Rearing Environment and Risk for Major Depression: A Swedish National High-Risk Home-Reared and Adopted-Away Co-Sibling Control Study. *American Journal of Psychiatry*, 177(5), 447–453. https://doi.org/10.1176/appi.ajp.2019.19090911

Kersten-Alvarez, L. E., Hosman, C. M. H., Riksen-Walraven, J. M., Van Doesum, K. T. M., & Hoefnagels, C. (2010). Long-term effects of a home-visiting intervention for depressed mothers and their infants. *Journal of Child Psychology and Psychiatry and Allied Disciplines*, 51(10), 1160–1170. https://doi.org/10.1111/j.1469-7610.2010.02268.x

Klasen, F., Otto, C., Kriston, L., Patalay, P., Schlack, R., & Ravens-Sieberer, U. (2015). Risk and protective factors for the development of depressive symptoms in children and adolescents: results of the longitudinal BELLA study. *European Child and Adolescent Psychiatry*, 24(6), 695–703. https://doi.org/10.1007/s00787-014-0637-5

Knoche, L. L., Givens, J. E., & Sheridan, S. M. (2007). Risk and protective factors for children of adolescents: Maternal depression and parental sense of competence. *Journal of Child and Family Studies*, 16(5), 684–695. https://doi.org/10.1007/s10826-006-9116-z

Kotch, J. B., Smith, J., Margolis, B., Black, M. M., English, D., Thompson, R., Lee, L. C., Taneja, G., & Bangdiwala, S. I. (2014). Does social capital protect against the adverse behavioural outcomes of child neglect? *Child Abuse Review*, 23(4), 246–261. https://doi.org/10.1002/car.2345

Küçük, L., Bilgin, H., Çömez Ikican, T., Kaçar, S., Kadak, M. T., Demirel, Ö. F., Çoban, V., Kutlu, F. Y., Aksoy Poyraz, C., Buzlu, S., & Duran, A. (2020). Evaluation of the Effectiveness of Psychoeducation Given to Children with Parental Psychiatric Disorders. *Issues in Mental Health Nursing*, 41(11), 985–994. https://doi.org/10.1080/01612840.2020.1756010

Kujawa, A., Hajcak, G., & Klein, D. N. (2019). Reduced reward responsiveness moderates the effect of maternal depression on depressive symptoms in offspring: evidence across levels of analysis. *Journal of Child Psychology and Psychiatry and Allied Disciplines*, 60(1), 82–90. https://doi.org/10.1111/jcpp.12944

Kujawa, A., Proudfit, G. H., Laptook, R., & Klein, D. N. (2015). Early parenting moderates the association between parental depression and neural reactivity to rewards and losses in offspring. *Clinical Psychological Science*, 3(4), 503–515. https://doi.org/10.1177/2167702614542464

Kwok, S. Y. C. L., & Gu, M. (2020). Parental Suicidal Ideation and Child Depressive Symptoms: The Roles of Optimism and Gratitude. *Journal of Social Service Research*, 46(4), 586–595. https://doi.org/10.1080/01488376.2019.1612819

Lachman, A., Niehaus, D. J. H., Jordaan, E. R., Leppanen, J., Puura, K., & Bruwer, B. (2021). Shared Pleasure in early mother–infant interactions: a study in a high-risk South African sample. *Early Child Development and Care*, 191(2), 230–241. https://doi.org/10.1080/03004430.2019.1613651

Lee, A. Y., Kim, S. O., Gim, G. M., Kim, D. S., & Park, S. A. (2020). Care farming program for family health: A pilot study with mothers and children. *International Journal of Environmental Research and Public Health*, 17(1). https://doi.org/10.3390/ijerph17010027

Lee, L. C., Halpern, C. T., Hertz-Picciotto, I., Martin, S. L., & Suchindran, C. M. (2006). Child care and social support modify the association between maternal depressive symptoms and early childhood behaviour problems: A US national study. *Journal of Epidemiology and Community Health*, 60(4), 305–310. https://doi.org/10.1136/jech.2005.040956

Letourneau, N., Leung, B., Ntanda, H., Dewey, D., Deane, A. J., & Giesbrecht, G. F. (2019). Maternal and paternal perinatal depressive symptoms associate with 2-and 3-year-old children’s behaviour: Findings from the APrON longitudinal study. *BMC Pediatrics*, 19(1). https://doi.org/10.1186/s12887-019-1775-1

Lewandowski, R. E., Verdeli, H., Wickramaratne, P., Warner, V., Mancini, A., & Weissman, M. (2014). Predictors of Positive Outcomes in Offspring of Depressed Parents and Non-depressed Parents Across 20 Years. *Journal of Child and Family Studies*, 23(5), 800–811. https://doi.org/10.1007/s10826-013-9732-3

Lewin, A., Mitchell, S. J., Waters, D., Hodgkinson, S., Southammakosane, C., & Gilmore, J. (2015). The Protective Effects of Father Involvement for Infants of Teen Mothers with Depressive Symptoms. *Maternal and Child Health Journal*, 19(5), 1016–1023. https://doi.org/10.1007/s10995-014-1600-2

Li, X., Weissman, M., Talati, A., Svob, C., Wickramaratne, P., Posner, J., & Xu, D. (2019). A diffusion tensor imaging study of brain microstructural changes related to religion and spirituality in families at high risk for depression. *Brain and Behavior*, 9(2). https://doi.org/10.1002/brb3.1209

Lichenstein, S. D., Shaw, D. S., Musselman, S., & Forbes, E. E. (2015). Dorsal ACC Response to Negative Feedback as a Potential Marker of Resilience to Depression among High-Risk Young Men. *BIOLOGICAL PSYCHIATRY*, 77(9), 387S-387S.

Liu, P., Vandermeer, M. R. J., Joanisse, M. F., Barch, D. M., Dozois, D. J. A., & Hayden, E. P. (2020). Neural Activity During Self-referential Processing in Children at Risk for Depression. *Biological Psychiatry: Cognitive Neuroscience and Neuroimaging*, 5(4), 429–437. https://doi.org/10.1016/j.bpsc.2019.12.012

Loechner, J., Sfärlea, A., Starman, K., Oort, F., Thomsen, L. A., Schulte-Körne, G., & Platt, B. (2020). Risk of Depression in the Offspring of Parents with Depression: The Role of Emotion Regulation, Cognitive Style, Parenting and Life Events. *Child Psychiatry and Human Development*, 51(2), 294–309. https://doi.org/10.1007/s10578-019-00930-4

Luthar, S. S., & Sexton, C. C. (2020). Maternal drug abuse versus maternal depression: Vulnerability and resilience among school-age and adolescent offspring. *Development and Psychopathology*, 19(1), 205-225.

Lyons-Ruth, K., Connell, D. B., Grunebaum, H. U., & Botein, S. (1990). Infants at Social Risk: Maternal Depression and Family Support Services as Mediators of Infant Development and Security of Attachment. *Child development*, 61(1), 85-98.

Ma, S. S., Zhu, D. M., Yin, W. J., Hao, J. H., Huang, K., Tao, F. B., ... & Zhu, P. (2021). The role of neonatal vitamin D in the association of prenatal depression with toddlers ADHD symptoms: A birth cohort study. *Journal of Affective Disorders*, 281, 390-396.

Mahedy, L., Harold, G. T., Maughan, B., Gardner, F., Araya, R., Bevan Jones, R., Hammerton, G., Sellers, R., Thapar, A., & Collishaw, S. (2018). Resilience in high-risk adolescents of mothers with recurrent depressive disorder: The contribution of fathers. *Journal of Adolescence*, 65, 207–218. https://doi.org/10.1016/j.adolescence.2018.03.016

Malmberg, L. E., & Flouri, E. (2011). The comparison and interdependence of maternal and paternal influences on young children’s behavior and resilience. *Journal of Clinical Child and Adolescent Psychology*, 40(3), 434–444. https://doi.org/10.1080/15374416.2011.563469

Manczak, E. M., Donenberg, G. R., & Emerson, E. (2018). Can Mother–Daughter Communication Buffer Adolescent Risk for Mental Health Problems Associated With Maternal Depressive Symptoms? *Journal of Clinical Child and Adolescent Psychology*, 47(sup1), S509–S519. https://doi.org/10.1080/15374416.2018.1443458

Martinson, L. E., Esposito-Smythers, C., & Blalock, D. V. (2016). The effects of parental mental health and social-emotional coping on adolescent eating disorder attitudes and behaviors. *Journal of Adolescence*, 52, 154–161. https://doi.org/10.1016/j.adolescence.2016.08.007

McDonald, S. W., Kehler, H. L., & Tough, S. C. (2016). Protective factors for child development at age 2 in the presence of poor maternal mental health: results from the All Our Babies (AOB) pregnancy cohort. *BMJ open*, 6(11), e012096.

McManus, B. M., Blanchard, Y., Murphy, N. J., & Nugent, J. K. (2020). The effects of the Newborn Behavioral Observations (NBO) system in early intervention: A multisite randomized controlled trial. *Infant Mental Health Journal*, 41(6), 757–769. https://doi.org/10.1002/imhj.21882

Mechling, B. M. (2015). A cross-sectional survey of the effect on emerging adults living with a depressed parent. *Journal of Psychiatric and Mental Health Nursing*, 22(8), 570–578. https://doi.org/10.1111/jpm.12244

Milan, S., Snow, S., & Belay, S. (2009). Depressive Symptoms in Mothers and Children: Preschool Attachment as a Moderator of Risk. *Developmental Psychology*, 45(4), 1019–1033. https://doi.org/10.1037/a0016164

Milgrom, J., Hirshler, Y., Reece, J., Charlene, C. H., & Gemmill Alan, A. W. (2019). Social support—a protective factor for depressed perinatal women? *International Journal of Environmental Research and Public Health*, 16(8). https://doi.org/10.3390/ijerph16081426

Milgrom, J., Holt, C., Schembri, C., & Gemmill, A. (2015). Pilot results on child outcomes of antenatal depression treatment. *Archives of Women’s Mental Health*, 18 (2), 372. https://doi.org/http://dx.doi.org/10.1007/s00737-014-0488-6

Miller, L., Warner, V., Wickramaratne, P., & Weissman, M. (1997). Religiosity and depression: Ten-year follow-up of depressed mothers and offspring. *Journal of the American Academy of Child and Adolescent Psychiatry*, 36(10), 1416–1425. https://doi.org/10.1097/00004583-199710000-00024

Miller, L., Wickramaratne, P., Gameroff, M. J., Sage, M., Tenke, C. E., & Weissman, M. M. (2012). Religiosity and Major Depression in Adults at High Risk: A Ten-Year Prospective Study. *American Journal of Psychiatry*, 169(1), 89–94. https://doi.org/10.1176/appi.ajp.2011.10121823

Monti, J. D., & Rudolph, K. D. (2017). Maternal depression and trajectories of adolescent depression: The role of stress responses in youth risk and resilience. *Development and Psychopathology*, 29(4), 1413–1429. https://doi.org/10.1017/S0954579417000359

Morgan, J. K., Shaw, D. S., & Forbes, E. E. (2014). Maternal depression and warmth during childhood predict age 20 neural response to reward. *Journal of the American Academy of Child and Adolescent Psychiatry*, 53(1). https://doi.org/10.1016/j.jaac.2013.10.003

Morris, M. C., Ciesla, J. A., & Garber, J. (2008). A Prospective Study of the Cognitive-Stress Model of Depressive Symptoms in Adolescents. *Journal of Abnormal Psychology*, 117(4), 719–734. https://doi.org/10.1037/a0013741

Murray, L., Arteche, A., Fearon, P., Halligan, S., Goodyer, I., & Cooper, P. (2011). Maternal postnatal depression and the development of depression in offspring Up to 16 years of age. *Journal of the American Academy of Child and Adolescent Psychiatry*, 50(5), 460–470. https://doi.org/10.1016/j.jaac.2011.02.001

NICHD Early Child Care Research Network. (1999). Chronicity of maternal depressive symptoms, maternal sensitivity, and child functioning at 36 months. *Developmental Psychology*, 35(5), 1297–1310. https://doi.org/10.1037/0012-1649.35.5.1297

Olives, E. V., Forero, C. G., Maydeu-Olivares, A., Almansa, J., Palacio Vieira, J. A., Valderas, J. M., Ferrer, M., Rajmil, L., & Alonso, J. (2013). Environmental risk and protective factors of adolescents’ and youths’ mental health: Differences between parents’ appraisal and self-reports. *Quality of Life Research*, 22(3), 613–622. https://doi.org/10.1007/s11136-012-0167-x

Osborn, A. F. (1990). Resilient children: A longitudinal study of high achieving socially disadvantaged children. *Early Child Development and Care*, 62(1), 23–47. https://doi.org/10.1080/0300443900620103

Owens, E. B., & Shaw, D. S. (2003). Predicting Growth Curves of Externalizing Behavior Across the Preschool Years. *Journal of abnormal child psychology*, 31, 575-590.

Pargas, R. C. M., Brennan, P. A., Hammen, C., & Le Brocque, R. (2010). Resilience to Maternal Depression in Young Adulthood. *Developmental Psychology*, 46(4), 805–814. https://doi.org/10.1037/a0019817

Pesonen, A. K., Räikkönen, K., Strandberg, T., Keltikangas-Järvinen, L., & Järvenpää, A. L. (2004). Insecure adult attachment style and depressive symptoms: Implications for parental perceptions of infant temperament. *Infant Mental Health Journal*, 25(2), 99–116. https://doi.org/10.1002/imhj.10092

Petterson, S. M., & Albers, A. B. (2001). Effects of Poverty and Maternal Depression on Early Child Development. *Child Development*, 72(6), 1794–1813. https://doi.org/10.1111/1467-8624.00379

Pizeta, F. A., Silva, A. P. C., & Loureiro, S. R. (2017). Resilience to Cumulative Stressors: A Prediction Study of Schoolchildren Living With Maternal Depression. *Perspectives in Psychiatric Care*, 53(4), 321–328. https://doi.org/10.1111/ppc.12183

Plass-Christl, A., Otto, C., Klasen, F., Wiegand-Grefe, S., Barkmann, C., Hölling, H., Schulte-Markwort, M., & Ravens-Sieberer, U. (2018). Trajectories of mental health problems in children of parents with mental health problems: results of the BELLA study. *European Child and Adolescent Psychiatry*, 27(7), 867–876. https://doi.org/10.1007/s00787-017-1084-x

Platt, R., Weiss-Laxer, N. S., Creedon, T. B., Roman, M. J. S., Cardemil, E. V., & Cook, B. (2020). Association between maternal and child mental health among US Latinos: variation by nativity, ethnic subgroup, and time in the USA. *Archives of Women’s Mental Health*, 23(3), 421–428. https://doi.org/10.1007/s00737-019-00982-4

Pratt, M., Apter-Levi, Y., Vakart, A., Feldman, M., Fishman, R., Feldman, T., Zagoory-Sharon, O., & Feldman, R. (2015). Maternal depression and child oxytocin response; Moderation by maternal oxytocin and relational behavior. *Depression and Anxiety*, 32(9), 635–646. https://doi.org/10.1002/da.22392

Priel, A., Djalovski, A., Zagoory-Sharon, O., & Feldman, R. (2019). Maternal depression impacts child psychopathology across the first decade of life: Oxytocin and synchrony as markers of resilience. *Journal of Child Psychology and Psychiatry and Allied Disciplines*, 60(1), 30–42. https://doi.org/10.1111/jcpp.12880

Priel, A., Zeev-Wolf, M., Djalovski, A., & Feldman, R. (2020). Maternal depression impairs child emotion understanding and executive functions: The role of dysregulated maternal care across the first decade of life. *Emotion*, 20(6), 1042–1058. https://doi.org/10.1037/emo0000614

Punamäki, R. L., Paavonen, J., Toikka, S., & Solantaus, T. (2013). Effectiveness of preventive family intervention in improving cognitive attributions among children of depressed parents: A randomized study. *Journal of Family Psychology*, 27(4), 683–690. https://doi.org/10.1037/a0033466

Radke-Yarrow, M., & Brown, E. (1993). Resilience and vulnerability in children of multiple-risk families. *Development and Psychopathology*, 5(4), 581-592.

Rae-Grant, N., Thomas, B. H., Offord, D. R., & Boyle, M. H. (1989). Risk, Protective Factors, and the Prevalence of Behavioral and Emotional Disorders in Children and Adolescents. *Journal of the American Academy of Child and Adolescent Psychiatry*, 28(2), 262–268. https://doi.org/10.1097/00004583-198903000-00019

Ranøyen, I., Stenseng, F., Klöckner, C. A., Wallander, J., & Jozefiak, T. (2015). Familial aggregation of anxiety and depression in the community: The role of adolescents’ self-esteem and physical activity level (the hunt study). *BMC Public Health*, 15(1). https://doi.org/10.1186/s12889-015-1431-0

Rieder, A. D., Roth, S. L., Musyimi, C., Ndetei, D., Sassi, R. B., Mutiso, V., Hall, G. B., & Gonzalez, A. (2019). Impact of maternal adverse childhood experiences on child socioemotional function in rural Kenya: Mediating role of maternal mental health. *Developmental Science*, 22(5). https://doi.org/10.1111/desc.12833

Riley, A., Coiro, M. J., Broitman, M., Colantuoni, E., Hurley, K., Bandeen-Roche, K., & Miranda, J. (2009). Mental Health of Children of Low-Income Depressed Mothers: Influences of Parenting, Family Environment, and Raters. *Psychiatric Services*, 60(3). https://doi.org/10.1176/appi.ps.60.3.329

Roman, G. D., Ensor, R., & Hughes, C. (2016). Does executive function mediate the path from mothers’ depressive symptoms to young children’s problem behaviors? *Journal of Experimental Child Psychology*, 142, 158–170. https://doi.org/10.1016/j.jecp.2015.09.022

Rounding, K., Jacobson, J. A., & Hart, K. E. (2016). Religiosity as a moderator of causal uncertainty’s mediational role in the parental-offspring dysphoria relationship. *Psychology of Religion and Spirituality*, 8(1), 1–12. https://doi.org/10.1037/a0039690

Sacchi, C., De Carli, P., Vieno, A., Piallini, G., Zoia, S., & Simonelli, A. (2018). Does infant negative emotionality moderate the effect of maternal depression on motor development? *Early Human Development*, 119, 56–61. https://doi.org/10.1016/j.earlhumdev.2018.03.006

Salisbury, A. L., Lester, B. M., Seifer, R., LaGasse, L., Bauer, C. R., Shankaran, S., Bada, H., Wright, L., Liu, J., & Poole, K. (2007). Prenatal cocaine use and maternal depression: Effects on infant neurobehavior. *Neurotoxicology and Teratology*, 29(3), 331–340. https://doi.org/10.1016/j.ntt.2006.12.001

Sang, J., Cederbaum, J. A., Ko, A. C., & Hurlburt, M. S. (2019). Maternal Depressive Symptoms, Adolescent Daughters’ Substance Use, and Father Residence in Minority Families. *Substance Use and Misuse*, 54(11), 1774–1786. https://doi.org/10.1080/10826084.2019.1610446

Sardana, S., Renaud, A., Jean-Pierre, A., Cheng, B., & Verdeli, H. (2020). It takes a village to save a life in Northern Uganda: Recommendations on suicide risk mitigation in depressed mothers within a clinical trial. *Behaviour Research and Therapy*, 130, 103645. https://doi.org/http://dx.doi.org/10.1016/j.brat.2020.103645

Savage-McGlynn, E., Redshaw, M., Heron, J., Stein, A., Quigley, M. A., Evans, J., Ramchandani, P., & Gray, R. (2015). Mechanisms of resilience in children of mothers who self-report with depressive symptoms in the first postnatal year. *PLoS ONE*, 10(11). https://doi.org/10.1371/journal.pone.0142898

Schechter, J. C., Brennan, P. A., Smith, A. K., Stowe, Z. N., Newport, D. J., & Johnson, K. C. (2017). Maternal Prenatal Psychological Distress and Preschool Cognitive Functioning: the Protective Role of Positive Parental Engagement. *Journal of Abnormal Child Psychology*, 45(2), 249–260. https://doi.org/10.1007/s10802-016-0161-9

Schiff, M., Pat-Horenczyk, R., Ziv, Y., & Brom, D. (2021). Multiple Traumas, Maternal Depression, Mother–Child Relationship, Social Support, and Young Children’s Behavioral Problems. *Journal of Interpersonal Violence*, 36(1–2), 892–914. https://doi.org/10.1177/0886260517725738

Seifer, R., Sameroff, A. J., Baldwin, C. P., & Baldwin, A. (1992). Child and Family Factors that Ameliorate Risk between 4 and 13 Years of Age. *Journal of the American Academy of Child and Adolescent Psychiatry*, 31(5), 893–903. https://doi.org/10.1097/00004583-199209000-00018

Shannon, K. E., Beauchaine, T. P., Brenner, S. L., Neuhaus, E., & Gatzke-Kopp, L. (2007). Familial and temperamental predictors of resilience in children at risk for conduct disorder and depression. *Development and Psychopathology*, 19(3), 701–727. https://doi.org/10.1017/S0954579407000351

Silk, J. S., Shaw, D. S., Forbes, E. E., Lane, T. L., & Kovacs, M. (2006). Maternal depression and child internalizing: The moderating role of child emotion regulation. *Journal of Clinical Child and Adolescent Psychology*, 35(1), 116–126. https://doi.org/10.1207/s15374424jccp3501_10

Silk, J. S., Vanderbilt-Adriance, E., Shaw, D. S., Forbes, E. E., Whalen, D. J., Ryan, N. D., & Dahl, R. E. (2007). Resilience among children and adolescents at risk for depression: Mediation and moderation across social and neurobiological contexts. *Development and Psychopathology*, 19(3), 841–865. https://doi.org/10.1017/S0954579407000417

Sitnick, S. L., Shaw, D. S., & Hyde, L. W. (2014). Precursors of adolescent substance use from early childhood and early adolescence: Testing a developmental cascade model. *Development and Psychopathology*, 26(1), 125–140. https://doi.org/10.1017/S0954579413000539

Steele, E. H., & McKinney, C. (2020). Relationships Among Emerging Adult Psychological Problems, Maltreatment, and Parental Psychopathology: Moderation by Parent–Child Relationship Quality. *Family Process*, 59(1), 257–272. https://doi.org/10.1111/famp.12407

Sticca, F., Wustmann Seiler, C., & Gasser-Haas, O. (2020). Familial Risk Factors and Emotional Problems in Early Childhood: The Promotive and Protective Role of Children’s Self-Efficacy and Self-Concept. *Frontiers in Psychology*, 11. https://doi.org/10.3389/fpsyg.2020.547368

Stoeckel, M., Weissbrod, C., & Ahrens, A. (2015). The Adolescent Response to Parental Illness: The Influence of Dispositional Gratitude. *Journal of Child and Family Studies*, 24(5), 1501–1509. https://doi.org/10.1007/s10826-014-9955-y

Stopsack, M., Ulrich, I., Aldinger, M., Reinelt, E., & Barnow, S. (2012). Children of mentally ill parents: Familial transmission pathways. *Adolescent Psychiatry*, 2 (1), 82.

Sun, W., Li, D., Zhang, W., Bao, Z., & Wang, Y. (2015). Family material hardship and Chinese adolescents’ problem behaviors: A moderated mediation analysis. *PLoS ONE*, 10(5). https://doi.org/10.1371/journal.pone.0128024

Svob, C., & Weissman, M. M. (2019). The role of religiosity in families at high-risk for depression. *Ethics, Medicine and Public Health*, 9, 1-6.

Swetlitz, C., Lynch, S. F., Propper, C. B., Coffman, J. L., & Wagner, N. J. (2021). Examining Maternal Elaborative Reminiscing as a Protective Factor in the Intergenerational Transmission of Psychopathology. *Research on Child and Adolescent Psychopathology*, 49(8), 989–999. https://doi.org/10.1007/s10802-021-00790-4

Szkody, E., & McKinney, C. (2019). Stress-Buffering Effects of Social Support on Depressive Problems: Perceived vs. Received Support and Moderation by Parental Depression. *Journal of Child and Family Studies*, 28(8), 2209–2219. https://doi.org/10.1007/s10826-019-01437-1

Talati, A. (2014). Variation in serotonin transporter promoter linked polymorphisms (5HTTLPR) associated with familial risk for major depressive disorder. *Biological Psychiatry*, 75(9), 344S. https://doi.org/http://dx.doi.org/10.1016/j.biopsych.2014.03.016

Tannenbaum, L., & Forehand, R. (1994). Maternal depressive mood: the role of the father in preventing adolescent problem behaviors. *Behaviour Research and Therapy*, 32(3), 321–325.

Taraban, L., Feldman, J. S., Wilson, M. N., Dishion, T. J., & Shaw, D. S. (2020). Sad Dads and Troubled Tots: Protective Factors Related to the Stability of Paternal Depression and Early Childhood Internalizing Problems. *Journal of Abnormal Child Psychology*, 48(7), 935–949. https://doi.org/10.1007/s10802-020-00649-0

Thompson, R. J., Mata, J., Gershon, A., & Gotlib, I. H. (2017). Adaptive coping mediates the relation between mothers’ and daughters’ depressive symptoms: A moderated mediation study. *Journal of Social and Clinical Psychology*, 36(3), 171-195.

Tiet, Q. Q., Bird, H. R., Davies, M., Hoven, C., Cohen, P., Jensen, P. S., & Goodman, S. (1998). Adverse life events and resilience. *Journal of the American Academy of Child and Adolescent Psychiatry*, 37(11), 1191–1200. https://doi.org/10.1097/00004583-199811000-00020

Tolliver-Lynn, M. N., Marris, A. M., Sullivan, M. A., & Armans, M. (2021). The role of the parent–child relationship in fostering resilience in American Indian/Alaskan Native children. *Journal of Community Psychology*, 49(2), 419–431. https://doi.org/10.1002/jcop.22468

Turney, K. (2011). Chronic and proximate depression among mothers: Implications for child well-being. *Journal of Marriage and Family*, 73(1), 149–163. https://doi.org/10.1111/j.1741-3737.2010.00795.x

Vakrat, A., Apter-Levy, Y., & Feldman, R. (2018). Sensitive Fathering Buffers the Effects of Chronic Maternal Depression on Child Psychopathology. *Child Psychiatry and Human Development*, 49(5), 779–785. https://doi.org/10.1007/s10578-018-0795-7

Valdez, C. R., Mills, C. L., Barrueco, S., Leis, J., & Riley, A. W. (2011). A pilot study of a family-focused intervention for children and families affected by maternal depression. *Journal of Family Therapy*, 33(1), 3–19. https://doi.org/10.1111/j.1467-6427.2010.00529.x

Valdez, C. R., Padilla, B., Moore, S. M., & Magaña, S. (2013). Feasibility, acceptability, and preliminary outcomes of the fortalezas familiares intervention for latino families facing maternal depression. *Family Process*, 52(3), 394–410. https://doi.org/10.1111/famp.12033

Van Der Zanden, R. A. P., Speetjens, P. A. M., Arntz, K. S. E., & Onrust, S. A. (2010). Online group course for parents with mental illness: Development and pilot study. *Journal of Medical Internet Research*, 12(5). https://doi.org/10.2196/jmir.1394

Van Loon, L. M. A., Van De Ven, M. O. M., Van Doesum, K. T. M., Hosman, C. M. H., & Witteman, C. L. M. (2015). Factors Promoting Mental Health of Adolescents Who Have a Parent with Mental Illness: A Longitudinal Study. *Child and Youth Care Forum*, 44(6), 777–799. https://doi.org/10.1007/s10566-015-9304-3

Van Santvoort, F., Hosman, C. M. H., Van Doesum, K. T. M., & Janssens, J. M. A. M. (2014). Effectiveness of preventive support groups for children of mentally ill or addicted parents: A randomized controlled trial. *European Child and Adolescent Psychiatry*, 23(6), 473–484. https://doi.org/10.1007/s00787-013-0476-9

Vreeland, A., Bettis, A. H., Reising, M. M., Dunbar, J. P., Watson, K. H., Gruhn, M. A., & Compas, B. E. (2019). Coping and Stress Reactivity as Moderators of Maternal Depressive Symptoms and Youth’s Internalizing and Externalizing Symptoms. *Journal of Youth and Adolescence*, 48(8), 1580–1591. https://doi.org/10.1007/s10964-019-01033-y

Weikum, W. M., Brain, U., Chau, C. M. Y., Grunau, R. E., Boyce, W. T., Diamond, A., & Oberlander, T. F. (2013). Prenatal serotonin reuptake inhibitor (SRI) antidepressant exposure and serotonin transporter promoter genotype (SLC6A4) influence executive functions at 6 years of age. *Frontiers in Cellular Neuroscience*, OCT. https://doi.org/10.3389/fncel.2013.00180

West, K. B., Oshri, A., Mitaro, E., Caughy, M., & Suveg, C. (2020). Maternal Depression and Preadolescent Symptoms: An Examination of Dyad-Level Moderators in an Economically Impoverished Sample. *Journal of Family Psychology*. https://doi.org/10.1037/fam0000610

Woodhouse, S. S., Ramos-Marcuse, F., Ehrlich, K. B., Warner, S., & Cassidy, J. (2010). The role of adolescent attachment in moderating and mediating the links between parent and adolescent psychological symptoms. *Journal of Clinical Child and Adolescent Psychology*, 39(1), 51–63. https://doi.org/10.1080/15374410903401096

Wurster, H. E., Sarche, M., Trucksess, C., Morse, B., & Biringen, Z. (2020). Parents’ adverse childhood experiences and parent-child emotional availability in an American Indian community: Relations with young children’s social-emotional development. *Development and Psychopathology*, 32(2), 425–436. https://doi.org/10.1017/S095457941900018X

Yan, N. (2016). Children’s resilience in the presence of mothers’ depressive symptoms: Examining regulatory processes related to active agency. *Children and Youth Services Review*, 61, 90–100. https://doi.org/10.1016/j.childyouth.2015.12.008

Yan, N., Zhou, N., & Ansari, A. (2016). Maternal Depression and Children’s Cognitive and Socio-Emotional Development at First Grade: The Moderating Role of Classroom Emotional Climate. *Journal of Child and Family Studies*, 25(4), 1247–1256. https://doi.org/10.1007/s10826-015-0301-9

Yeh, Z. T., Huang, Y. hsuan, & Liu, S. I. (2016). Maternal Depression and Adolescent Emotions: The Role of Family Functioning. *Journal of Child and Family Studies*, 25(7), 2189–2200. https://doi.org/10.1007/s10826-016-0399-4

Yoon, S., Pei, F., Wang, X., Yoon, D., Lee, G., Shockley McCarthy, K., & Schoppe-Sullivan, S. J. (2018). Vulnerability or resilience to early substance use among adolescents at risk: The roles of maltreatment and father involvement. *Child Abuse & Neglect*, 86, 206–216. https://doi.org/10.1016/j.chiabu.2018.09.020

Yu, N. X., Kam-fung Liu, I., & Bu, H. (2021). Enhancing resilience in cross-boundary families: A parent–child parallel group intervention. *Journal of Social Work*, 21(4), 651–675. https://doi.org/10.1177/1468017320919103

Zelazny, J., Melhem, N., Porta, G., Biernesser, C., Keilp, J. G., Mann, J. J., Oquendo, M. A., Stanley, B., & Brent, D. A. (2019). Childhood maltreatment, neuropsychological function and suicidal behavior. *Journal of Child Psychology and Psychiatry and Allied Disciplines*, 60(10), 1085–1093. https://doi.org/10.1111/jcpp.13096

Zhang, W., Finik, J., Dana, K., Glover, V., Ham, J., & Nomura, Y. (2018). Prenatal Depression and Infant Temperament: The Moderating Role of Placental Gene Expression. *Infancy*, 23(2), 211–231. https://doi.org/10.1111/infa.12215
